# Long RNA Profiles of Human Brain Extracellular Vesicles Provide New Insights into the Pathogenesis of Alzheimer’s Disease

**DOI:** 10.14336/AD.2022.0607

**Published:** 2023-02-01

**Authors:** Dan Luo, Haotian Liu, Hanyou Liu, Wei Wu, Hanyang Zhu, Wei Ge, Chao Ma

**Affiliations:** ^1^Institute of Basic Medical Sciences, Department of Human Anatomy, Histology and Embryology, Neuroscience Center, Institute of Basic Medical Sciences, Chinese Academy of Medical Sciences, Beijing, China.; ^2^National Key Laboratory of Medical Molecular Biology & Department of Immunology, Institute of Basic Medical Sciences, Chinese Academy of Medical Sciences, Beijing, China.

**Keywords:** Alzheimer’s disease;, circRNA, lncRNA, mRNA, WGCNA

## Abstract

Alzheimer’s disease (AD) is the most common neurodegenerative disorder. Extracellular vesicles (EVs), carriers of nucleic acids, lipids, and proteins, are known to play significant roles in neurodegenerative pathogenesis. Studies have shown that EVs from AD human brain tissue contain toxic proteins that may lead to neuron cell damage and loss. However, the potential contribution of EV long RNAs (exLR) to AD pathobiology is less well known, and their biochemical functions and molecular properties remain obscure. Here, EVs were isolated from the frontal cortex of normal control (NC; N = 10) and AD (N = 8) brain tissue donors. We performed exLR profiling on the isolated EVs followed by pathway analysis and weighted gene co-expression network analysis (WGCNA). A total of 1012 mRNAs, 320 long non-coding RNAs (lncRNAs), and 119 circular RNAs (circRNAs) were found to be differentially expressed (DE) in AD-EVs compared with NC-EVs. Functional analysis of the DEmRNAs revealed that metal ion transport, calcium signaling, and various neuronal processes were enriched. To investigate the possible functions of the identified DElncRNAs and DEcircRNAs, competing endogenous RNA (ceRNA) networks were constructed and subjected to WGCNA, in which two gene modules were identified to be significantly correlated with AD. Moreover, we discovered that NC-EVs were more effective than AD-EVs in promoting cytokine expression, phagocytosis, and induction of calcium signaling in microglia. Our study provides an in-depth characterization of brain tissue exLR and identifies several RNAs that correlate with the pathogenesis of AD.

Alzheimer’s disease (AD) is the most common neurodegenerative disorder and is characterized by memory loss and cognitive dysfunction. The major pathological lesions of AD include extracellular Aβ plaques and intracellular neurofibrillary tangles [1]. In addition, neuronal loss, synaptic dysfunctions, and inflammatory responses are associated with AD [2, 3]. Although great efforts have been made to prevent the development of AD, no effective treatment exists. A better understanding of the molecular mechanisms underlying AD pathology could reveal new therapeutic strategies.

Extracellular vesicles (EVs), which are naturally released from most cells (including neurons, microglia, and astrocytes) into the extracellular space, contain multiple functional components in their cargo such as nucleic acids, proteins, and lipids [4, 5]. By transferring these functional components, EVs play a key role in cell-to-cell communication, signal transduction, and the spread of pathological proteins and nucleic acids. Recent studies have demonstrated that toxic proteins, like amyloid β-peptide 1-42 (Aβ1-42) and phosphorylated tau protein (p-tau) are secreted in the brain through EVs, leading to neuronal cell damage and loss [6, 7]. These findings suggest that brain-derived EVs may be a key pathological contributor to AD. However, there is also evidence that EVs might play a beneficial role in the clearance of Aβ in the AD brain [8]. One can conclude that EVs are heavily involved in the pathogenesis of AD. Numerous studies have investigated the role of EVs in AD using cell cultures and transgenic animal models. Although these studies have generated important data, the physiological and pathological roles of EVs in human brain tissue remain largely unexplored.

Noncoding RNAs, including microRNAs (miRNAs), long non-coding RNAs (lncRNAs), and circular RNAs (circRNAs), regulate gene expression in a wide array of human diseases, and thus may serve as biomarkers or potential therapeutic targets. In particular, accumulating evidence strongly suggests that noncoding RNAs participate in AD-related pathophysiology [9]. Few studies have reported the interplay of different RNA species in human brain tissue-derived EVs and how their expression profiles change in AD. In this study, we performed long RNA profiling to characterize the expression profiles of mRNAs, lncRNAs, and circRNAs in AD and normal control (NC) human brain tissue-derived EVs. It has previously been reported that lncRNAs and circRNAs may function as miRNA sponges [10, 11], meaning that they compete with each other through miRNA response elements (MREs) and constitute competing endogenous RNA (ceRNA) networks. Based on long RNA profiling in combination with miRNA prediction, we established a set of lncRNA-miRNA-mRNA and circRNA-miRNA-mRNA ceRNA regulatory networks, which may help to explain the molecular mechanisms underlying the involvement of brain tissue-derived EVs in AD.

Weighted gene coexpression network analysis (WGCNA) is a data exploratory tool for finding candidate biomarkers and therapeutic targets by identifying co-expression modules that are correlated with clinical traits [12]. We applied WGCNA to the RNA expression profiles of mRNAs, lncRNAs, and circRNAs generated from AD and NC human brain tissue-derived EVs to identify key co-expression modules. Through WGCNA analysis, we identified gene modules closely related to AD. Since microglia play an important role in the formation of plaques, we investigated the effects of brain tissue-derived EVs on the function of microglia. Interestingly, we unearthed the fact that NC-EVs can promote microglial phagocytosis and activation more strongly than AD-EVs. These results may provide novel insights for the development of improved assessment strategies and therapeutic tools for AD.

## MATERIALS AND METHODS

### Reagents and antibodies

HBSS (Hanks’ Balanced Salt Solution, 14175-095), 0.4% trypan blue stain (Cat#: 15250-061), and HEPES (*N*-2-hydroxyethylpiperazine-*N*-2-ethane sulfonic acid (Cat#: 15630-080) were purchased from Gibco (USA). Collagenase/dispase (Cat#: 10269638001), protease inhibitor cocktail (Cat#: 5056489001), fluorescent latex beads (Cat#: L1030), PKH26 Red Fluorescent Cell Linker Mini Kit (Cat#: MINI26-1KT), BSA (bovine serum albumin, SRE0096), and Immobilon Western Chemiluminescent HRP Substrate (Cat#: WBKLS0500) were purchased from Merck (Germany). PrimeScript RT Master Mix (Cat#: RR036) and Green Premix Ex *Taq* (Cat#: RR820) were purchased from TaKaRa (Japan). TRIzol reagent (Cat#: 15596018) and Fura 2, AM (Cat#: F1221) were purchased from ThermoFisher Scientific (USA). A RNeasy Micro Kit (Cat#: 74004) was obtained from Qiagen (USA). Carboxyfluorescein (FAM)-Aβ was synthesized by GL Biochem Ltd (China). Anti-CD9 (Cat#: ab223052, 1:1000 for western blotting and anti-CD63 (Cat#: ab8219, 1:500) were obtained from Abcam (UK). Anti-VDAC (Cat#: 4661, 1:1000), anti-Calnexin (Cat#: 2679, 1:1000), and anti-Bip (Cat#: 3177, 1:500) were obtained from Cell Signaling Technology (USA). Secondary antibodies were purchased from Cell Signaling Technology, as follows: anti-rabbit IgG (Cat#: 7074), and anti-mouse IgG, horseradish peroxidase (HRP)-linked antibody (Cat#: 7076).

### Tissue collection

The human brain tissues in this study were provided by the National Human Brain Bank for Development and Function, Chinese Academy of Medical Sciences and Peking Union Medical College, Beijing, China, from neuropathologically classified AD patients, and age and gender-matched controls (Tables 1 and 2). All donors had given informed consent to use the donated brain tissue for medical research, which, in accordance with the international standard human brain banking procedure, was collected from 2012 to 2021 [13]. The ‘ABC’ dementia score defined by the National Institute on Aging-Alzheimer’s Association guidelines was used for the neuropathological assessment of each sample [14].

**Table 1:** Clinical and demographic characteristics of the brain tissue donors.

Sample ID	Gender	Age at death	PMI	A	B	C	ABC score	Braak stage	ECog	ApoE
NC 1	M	80	7.5	0	2	0	N	III	NA	NA
NC 2	M	80	3	0	1	0	N	II	NA	ApoE3
NC 3	M	86	5	0	1	0	N	II	1.5	ApoE2
NC 4	M	87	3.5	0	1	0	N	II	1	ApoE3
NC 5	F	87	4	0	0	0	N	0	1	ApoE2
NC 6	F	81	7	0	0	0	N	0	1	ApoE3
NC 7	F	85	2.7	0	2	0	N	III	1	ApoE3
NC 8	M	78	21.5	0	1	0	N	II	1	NA
NC 9	M	70	7.5	0	1	0	N	II	1	ApoE3
NC 10	F	87	6	0	1	0	N	II	1	ApoE3
AD 1	F	86	6.33	3	2	3	H	IV	1.4	ApoE4
AD 2	M	87	11	3	3	2	H	V	2	ApoE2
AD 3	F	79	11	3	2	2	H	IV	1.1	ApoE3
AD 4	F	80	13	2	2	2	H	III	2.9	ApoE3
AD 5	M	80	4.5	0	1	0	I	II	4	ApoE3
AD 6	F	89	5	3	3	3	H	VI	1	ApoE3
AD 7	M	87	8.8	2	3	3	I	VI	4	ApoE3
AD 8	M	78	13.5	2	3	2	I	VI	3.9	NA

PMI: Post-mortem interval; A: Thal phase for Aβ amyloid plaques; B: Braak stage for neurofibrillary tangles; C: Consortium to Establish a Registry for Alzheimer’s Disease neuritic plaque frequency; ECog: The Everyday Cognition; ApoE: Apolipoprotein E.

### Purification of EVs from human brain samples

Unfixed frozen frontal cortical tissue (1 g) from deceased AD or NC donors was processed for EV extraction. The frozen brain tissue was chopped on ice using a scalpel to generate 5 mm^3^ sections. The sections were cut into pieces and transferred to 10 mL HBSS containing 100 μL collagenase/dispase, and then incubated in a shaking bed at 37°C for 30 min. After incubation, the samples were returned to ice and a protease inhibitor cocktail was added. The homogenized samples were filtered with a 40-µm mesh filter and centrifuged at 300 × *g* for 5 min before the pellets were collected (named ‘T1’). The supernatants from the 300 × *g* centrifugation run was transferred to new 15-mL tubes and then centrifuged at 2,000 × *g* for 10 min, after which the pellets were collected (named ‘T2’). The supernatants from the 2,000 × *g* run was transferred again to new 15-mL tubes and then centrifuged at 10,000 × *g* for 10 min. The pellets were collected (named ‘T3’). The supernatants from this spin were concentrated to 500 µL by ultrafiltration. The 500-µL samples were loaded on the top of a qEV original/70 nm size exclusion chromatography (SEC) column (IZON, USA), and fractions were eluted with phosphate-buffered saline (PBS) according to the manufacturer’s instructions. EVs were contained within fractions 7 and 8, 500 µl each, and were pooled in one tube.

### Identification of human brain tissue EVs

In accordance with the Minimal Information for Studies of Extracellular Vesicles 2018 (Cat#: MISEV2018) [15] guidelines, we used WB, transmission electron microscopy (TEM), and nanoparticle tracking analysis (NTA) to identify isolated brain tissue-derived EVs. For the WB assay, isolated EVs were homogenized in RIPA lysis buffer on ice for 30 min (well mixed every 10 min). EV proteins were sedimented by centrifugation, and the protein concentration was determined using a bicinchoninic acid assay. Proteins (20 μg) were separated using SDS-PAGE and transferred to polyvinylidene difluoride membranes. The membranes were first incubated with primary antibodies (anti-CD9, anti-CD63, anti-VDAC, anti-Calnexin, and anti-Bip) at 4 °C overnight, followed by a second incubation with HRP-conjugated secondary antibody for 1 h at room temperature. The results were visualized using an ECL detection reagent. For the TEM assay, EV samples were loaded onto a copper mesh and stained negatively with uranyl oxalate. The morphology of EVs was observed (TEM-1400plus, Hitachi). For the NTA assay, EVs were diluted with PBS and analyzed using a NanoSight NS300 instrument (Zetaview).

**Table 2:** Demographics of all brain donors

Category	NC	AD	*P*-value
Age	82.1 ± 5.5	83.3 ± 4.4	0.6
Gender (M/F)	6/4	4/4	0.3
PMI	6.8 ± 5.5	9.1 ± 3.5	0.3

Mean ± standard deviation; M, male; F, female; a T-test was used to detect differences in age and PMI, while a χ^2^-test was used for gender difference between the groups.

### RNA-sequencing workflow

The instructions of the RNeasy Micro Kit were carefully followed when extracting total RNA from isolated EVs. The RNA concentration and purity were measured using a Qubit 3.0 fluorimeter (Thermo Scientific) and an Agilent 2100 Bioanalyzer. RNA sequencing was performed using the Illumina HiSeq 3000 platform. Sequencing libraries were prepared using the Total RNA-Seq Library Prep Kit from Illumina. The reads were first mapped to the latest University of California, Santa Cruz (UCSC) genome browser transcript set using Bowtie2 version 2.1.0 and the gene expression level was estimated using RSEM v1.2.15. For lncRNA expression analysis, we used the transcript set from Lncipedia (www.lncipedia.org). TMM (trimmed mean of M-values) was used to normalize gene expression. Differentially expressed genes were identified using the R package edgeR. For circRNA expression analysis, the reads were mapped to the human genome using STAR, and DCC was used to annotate and approximate circRNA expression levels. TMM was used for normalization. Genes showing altered expression (>1.5-fold increase or < 0.67-fold decrease with *P*-value < 0.05) were considered to be differentially expressed. The data discussed in this publication have been deposited in NCBI’s Gene Expression Omnibus [16] and are accessible through GEO Series accession number GSE197505.

### EV labeling

A PKH26 Red Fluorescent Cell Linker Mini Kit was used for EV labeling. We dissolved 20 μg of EVs in 250 μL Diluent C, and 1 μL of PKH26 Cell Linker in another 250 μL of Diluent C. The two solutions were mixed and incubated at 37 °C for 10 min. BSA (500 μL, 1%) was added to terminate the reaction. Ultracentrifugation was then performed (200,000 × *g* at 4 °C for 90 min). The precipitate was obtained and resuspended in 100 μL PBS for further experiments.

### Microglial phagocytosis assay

BV2 microglia and primary microglia were incubated with EVs for 24 h. Following the removal of EVs, microglia were incubated with fluorescent latex beads (0.02%, v/v) for 2 h at 37 °C. To remove the latex beads, the cells were thoroughly washed with ice-cold PBS and then subjected to flow cytometry analysis using a CytoFLEX analyzer (Beckman). For Aβ phagocytosis assessment, the cells were incubated with FAM-Aβ (200 nM) for 2 h. Surface-bound FAM-Aβ was quenched by incubation with 0.4% trypan blue in PBS (pH 4.4) for 1 min before confocal microscopy analysis.

### Calcium measurements

Experiments were performed in HEPES buffer at room temperature. Ca^2+^ was monitored using the calcium-sensitive fluorescent indicator Fura-2 AM. BV2 cells were loaded with Fura-2, AM (2 μM) in the dark at 37 °C. After loading for 30 min, cells were washed twice with HEPES to remove extracellular dye. Ratiometric calcium imaging was performed using an upright Olympus BX-51WI microscope equipped with a ratiometric imaging system (INDEC Bio-systems, CA, USA). The calcium signals at 340 and 380 nm excitation were recorded at 2 s intervals. The ratio of 340 nm/380 nm fluorescence intensity within a specified region was used as a relative measure of intracellular calcium concentration.

### Quantitative real time-PCR (qRT-PCR)

Total RNA was extracted from EVs or cells using TRIzol reagent. cDNA was synthesized using PrimeScript RT Master Mix. qRT-PCR assays were performed with Green Premix Ex *Taq*. *GAPDH* was used as the reference gene for mRNA and lncRNA, while *U6* was used as the reference gene for circRNA. Relative gene expression was quantified using the 2^-ΔΔCt^ method [17] and each reaction was performed in triplicate. Primer sequences are listed in Supplementary Table 1.

### Bioinformatics

Gene Ontology (GO), the Kyoto Encyclopedia of Genes and Genomes (KEGG), and gene set enrichment analysis (GSEA) pathway analyses were conducted using the R package ‘clusterProfiler’ [18]. Significantly enriched GO terms were visualized using the R package ‘GOplot’ [19]. Network analysis of differentially expressed mRNAs was performed using the NetworkAnalyst platform [20]. The R package ‘WGCNA’ was used to conduct gene coexpression network analyses and to find clinical trait-related modules and hub genes. The non-default WGCNA parameters were: networkType = unsigned, corType = Pearson, Power = 9, minModuleSize = 30.

### Statistical analyses

Statistical analyses were performed using GraphPad Prism 7 software. The Shapiro-Wilk test was performed to test the normality of the data. *P* < 0.05 was considered statistically significant. In the microglial phagocytosis beads assay, a non-parametric test was used; in the microglial Aβ phagocytosis assay and calcium measurement assay, one-way analysis of variance was used; and in RT-PCR assays, a T-test was used.

## RESULTS

### Characterization of EVs isolated from post-mortem frontal cortex

**Figure 1.**
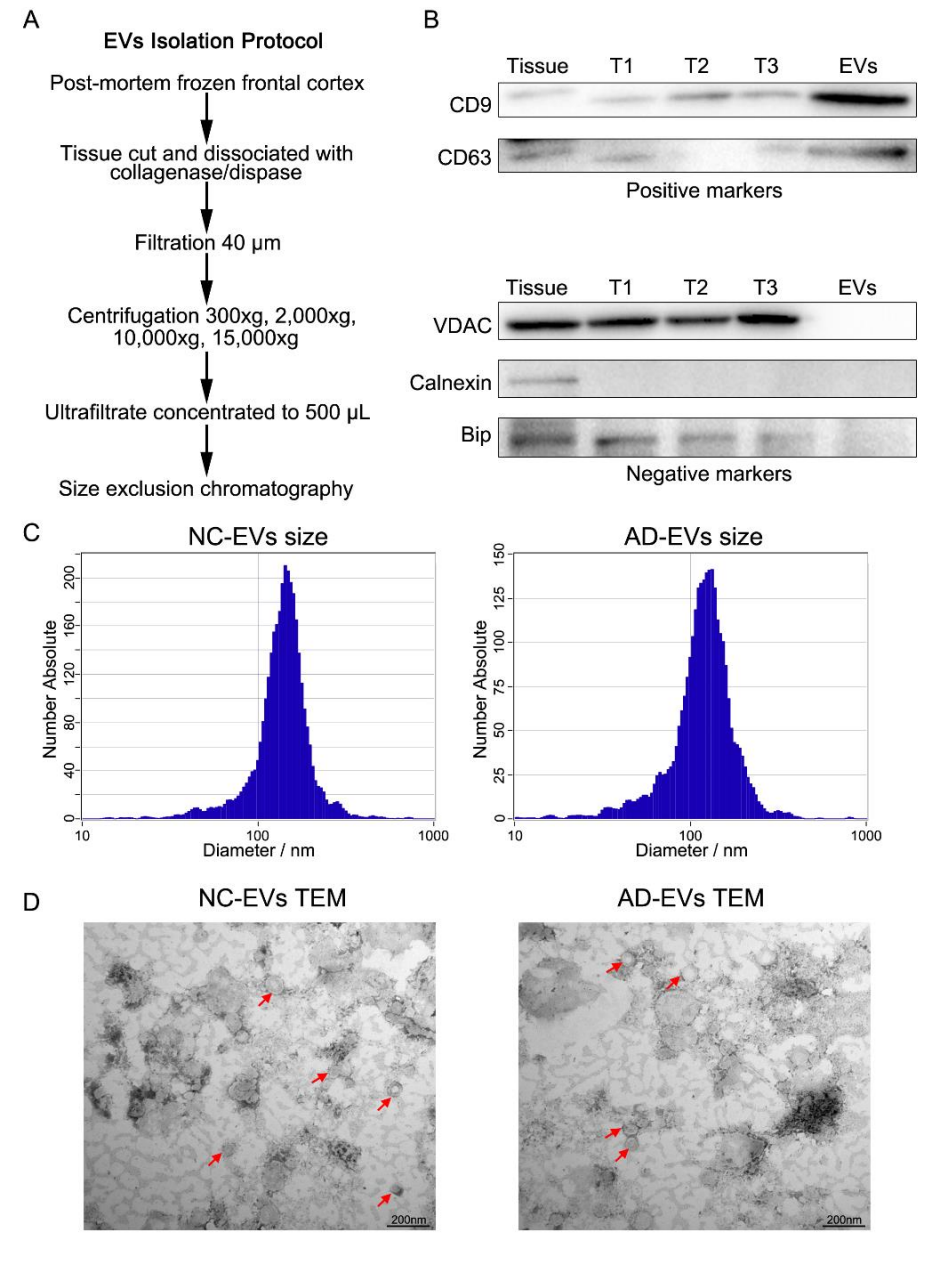
Characterization of extracellular vesicles (EVs) isolated from post-mortem human frontal cortex samples. (A) Schematic of the EV isolation protocol from the frontal cortex. (B) Western blot analysis of isolated EVs. (C) Size distribution of EVs as determined using nanoparticle tracking analysis. (D) EV morphology was visualized using transmission electron microscopy.

EVs were isolated from frontal cortex tissue originating from NC (N = 10) and AD (N = 8) individuals. We used collagenase and dispase to break down frontal cortex tissue. Following digestion, the tissue was subjected to a series of centrifugation steps to remove cell debris and microvesicles. The supernatant of samples was collected and concentrated (Fig. 1A). The final supernatant was applied to 70-nm SEC columns. Characterization of EVs isolated from AD and NC brain tissues is described in Supplementary Table 2. The purity of EVs was validated based on the enrichment of EV markers including CD9 and CD63, while mitochondrial proteins (using VDAC as a marker), endoplasmic reticulum (Calnexin), and Golgi (BiP) were not detectable (Fig. 1B). NTA was used to determine the distribution of EV size (Fig. 1C). EVs were imaged using TEM (Fig. 1D), the results of which indicated that the purity of our isolated EVs conformed to the MISEV2018 criteria.

**Figure 2.**
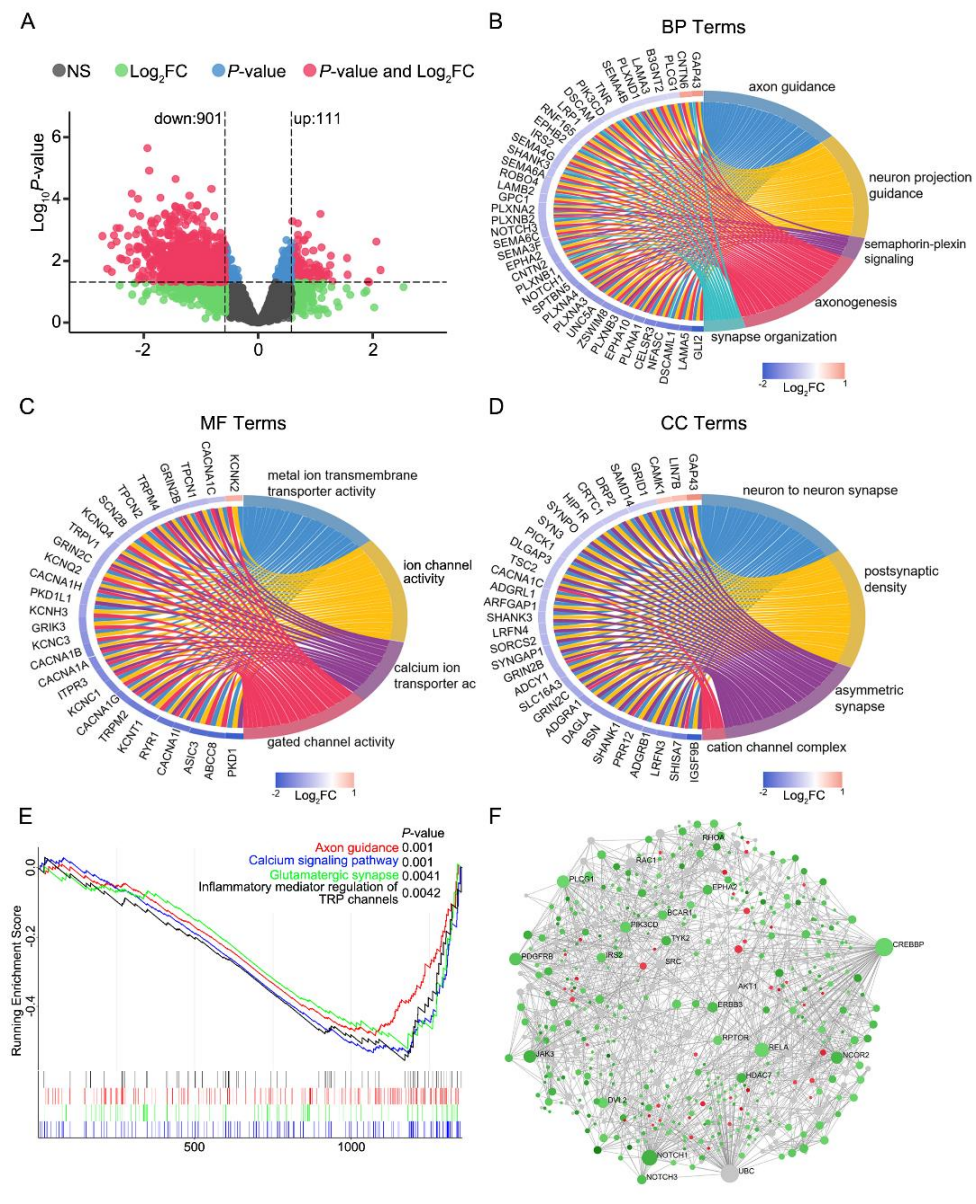
Normal control (NC) and Alzheimer’s disease (AD) EVs exhibit distinct mRNA profiles. (A) Volcano plot of differentially expressed mRNAs (DEmRNAs) in AD-EVs compared with NC-EVs. (B) Enriched biological processes identified by Gene Ontology (GO) analysis of DEmRNAs. (C) Enriched GO molecular functions. (D) Enriched GO cellular components. (E) Gene set enrichment analysis plot of signaling pathway enrichment in AD-EVs compared with NC-EVs. (F) Minimum network of DEmRNAs in AD-EVs. Red nodes represent upregulated mRNAs in AD-NVs compared with NC-EVs, green nodes represent downregulated mRNAs, and the saturation of the color denotes the log_2_ fold-change.

### NC and AD EVs exhibit distinct mRNA profiles

We performed long RNA profiling and mapping to several classes of RNA species. First, we determined differentially expressed mRNA (DEmRNA) profiles in EVs of AD subjects compared with NCs—111 upregulated and 901 downregulated DEmRNAs were identified (Fig. 2A). All of the DEmRNAs are shown in Supplementary Table 3. To investigate the functions and characteristics of the DEmRNAs, we performed GO enrichment analysis. The biological process (GO-BP) results indicated that the DEmRNAs were enriched in axon guidance, axonogenesis, and synapse organization. Most of the DEmRNAs in these GO-BP terms were downregulated (Fig. 2B). The molecular function (GO-MF) results indicated that the DEmRNAs were enriched in calcium and metal ion transmembrane transporter activity and ion and gated channel activity, which were generally downregulated (Fig. 2C). The cellular component (GO-CC) function results indicated that DEmRNAs were enriched in the neuron-to-neuron synapse, postsynaptic density, and asymmetric synapse terms, which were generally downregulated (Fig. 2D). The abovementioned results indicate the inactivation of axon guidance, axonogenesis, ion transporter activity, gated channel activity, and synapses in AD-EVs (Supplementary Table 4). Signaling pathway analysis demonstrated that DEmRNAs were mainly involved in calcium signaling (Supplementary Fig. 1A).

To further elucidate the up- and downregulated biological pathways in AD-EVs compared with NC-EVs, we performed GSEA. We observed the downregulation of axon guidance, glutamatergic synapse, inflammatory mediator regulation of transient receptor potential (TRP) channels, and calcium signaling pathways in AD-EVs (Fig. 2E). Mapping the DEmRNAs to the calcium signaling pathway uncovered the fact that most of the mRNAs were indeed downregulated (Supplementary Fig. 1B). The potential role of these pathways in brain tissue-derived EVs needs further investigation.

Furthermore, we matched the DEmRNAs to the specific cell expression profiles reported by Zhang *et al*. [21]. The results revealed that many DEmRNAs were enriched in astrocytes, neurons, oligodendrocytes, microglia, and endothelia, with the numbers of mRNAs being 142, 99, 47, 90, and 70 respectively (Supplementary Fig. 1C). Most of the DEmRNAs in the five types of cells were downregulated in AD-EVs relative to NC-EVs. These results suggest that neurons, glial cells, and vascular cells may be in a state of dysfunction in the AD human brain.

We performed a network-based analysis to identify key hub genes involved in the AD-EVs. A total of 764 nodes and 1838 edges were included in the interaction network. As shown in Fig. 2F, 22 mRNAs were identified as key hubs, with all the hub genes being downregulated in AD-EVs compared with NC-EVs. Among these, CREBBP has been identified as a key signaling and regulatory molecule associated with transcriptional changes in AD [22]. RELA is one of the most significant message factors in the NF-κB signaling pathway, which has been reported in neuroinflammation [23]. The level of Notch1 was significantly decreased in the plasma and cerebrospinal fluid of AD patients compared with normal [24]. Most of the other genes are previously unreported, and further studies are needed to prove our findings.

### Differentially expressed lncRNAs and functional enrichment analysis of lncRNA-related target genes

**Figure 3.**
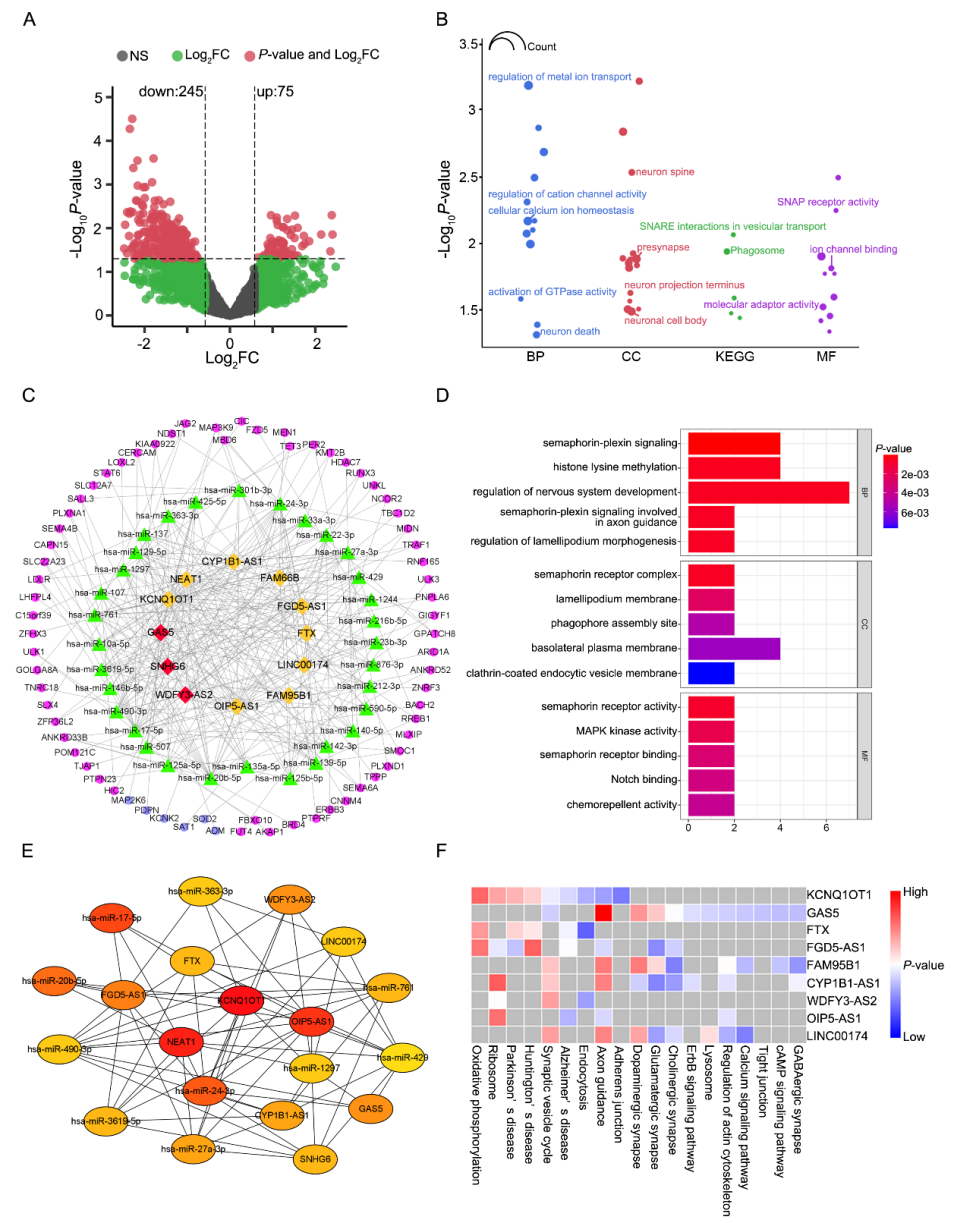
Differentially expressed long non-coding RNAs (lncRNAs) and functional enrichment analysis of lncRNA-related target genes. (A) Volcano plot of differentially expressed lncRNAs in AD-EVs compared with NC-EVs. (B) GO enrichment analysis of the *cis* target genes of lncRNAs. The size of the dots represents the number of genes in each term. (C) Competing endogenous RNA (CeRNA) networks were constructed based on lncRNA-miRNA and miRNA-mRNA interactions. The pink and purple nodes denote downregulated and upregulated RNAs, respectively. The red and yellow nodes represent upregulated and downregulated lncRNAs, respectively. (D) Functional enrichment analysis of lncRNA-associated ceRNA network genes. (E) Hub genes identified in the ceRNA network. (F) Top Kyoto Encyclopedia of Genes and Genomes (KEGG) pathways significantly correlated with the expression of hub lncRNAs.

Three hundred and twenty significantly dysregulated lncRNA transcripts (including 245 downregulated and 75 upregulated transcripts) were identified in the AD-EVs compared with NC-EVs (Fig. 3A, Supplementary Table 5). Because expression of lncRNAs often correlates with the expression of the parent or neighboring genes due to shared regulatory elements and the *cis*-regulatory role of lncRNA, we performed detailed annotations of the functions and characteristics of the *cis* target genes of the lncRNAs (Fig. 3B, Supplementary Table 6). GO-BP results showed that the *cis* target genes of the DElncRNAs were significantly enriched in metal ion transport, calcium ion homeostasis, GTPase activity, and neuron death. The enriched CC terms in GO analysis were neuron spine, presynapse, and neuronal cell body. The enrichment factors in GO-MF analysis were ion channel binding and SNAP receptor activity. It is noteworthy that these enrichment results share some common characteristics with those of the DEmRNAs, for example, the significant enrichment in the metal ion and neuron-related terms. In KEGG pathway enrichment analysis, the SNARE interactions in vesicular transport and phagosomes have been reported to be crucial pathways involved in AD pathogenesis [25, 26].

According to ceRNA theory, lncRNAs could act as miRNA sponges in AD development. We combined mRNA and lncRNA data from our transcriptomic sequencing to construct the ceRNA regulatory networks. Mircode database was used to predict lncRNA-miRNA pairs. MiRDB, miRTarBase, and TargetScan were used to predict miRNA-mRNA pairs, which showed 69 overlapping genes with the DEmRNAs. As shown in Figure 3C, the lncRNA-miRNA-mRNA ceRNA network consisted of 12 DElncRNAs (nine downregulated and three upregulated), 32 miRNAs, and 69 DEmRNAs (including six downregulated). GO analyses were performed on the genes included in the network identified here, and several GO terms were found to be significantly enriched. Several AD-associated terms were discovered, including semaphoring-plexin signaling, nervous system development, lamellipodium morphogenesis, phagophore assembly, and MAPK kinase activity (Fig. 3D). It is noteworthy that the ‘phagosome’ term was found in the KEGG analysis of the *cis* targets gene of the DElncRNAs and ‘phagophore assembly’ was found in the ceRNA network-related enrichment results, suggesting that DElncRNA-associated target genes might participate in the regulation of phagocytosis. Using the Cytoscape plug-in cytohubb, a hub-network was extracted from the lncRNA-miRNA-mRNA ceRNA network (Fig. 3E). The results demonstrated that OIP5-AS1, KCNQ1OT1, and lncRNA-NEAT1 were potential hub genes. These three hub lncRNAs have all been reported to play important functions in microglia-mediated inflammation [27-29]. Furthermore, we used the R2 KEGG Pathway Finder to perform a pathway enrichment analysis on lncRNAs from the ceRNA network. The pathways were then ranked by the sum of the negative log_10_ *P*-values of each lncRNA for each pathway (Fig. 3F). Interestingly, the pathways involved in the synaptic vesicle cycle, axon guidance, dopaminergic synapse, and Alzheimer’s disease were enriched within the lncRNA-correlated genes.

**Figure 4.**
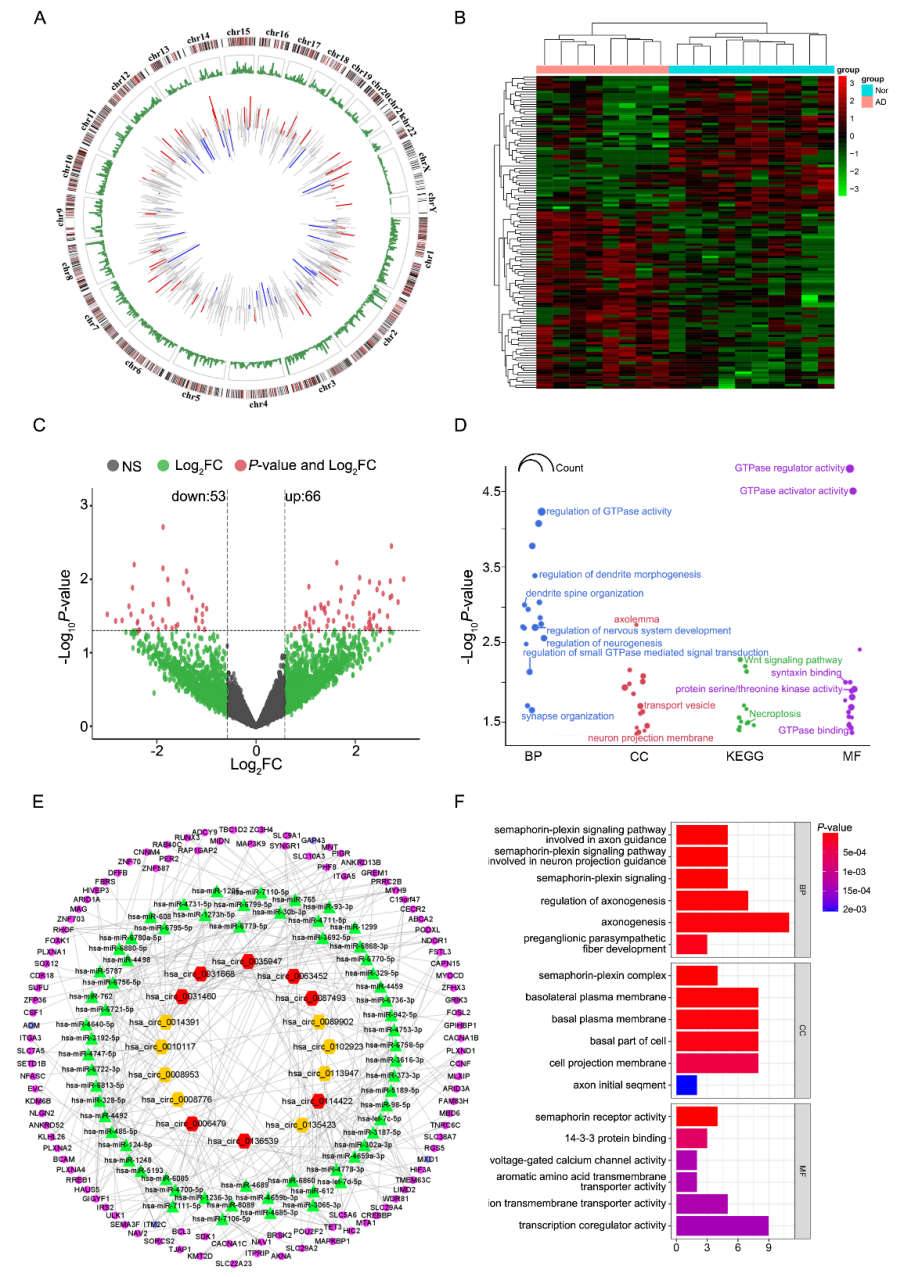
Differentially expressed circular RNAs (circRNAs) and functional enrichment analysis of circRNA-related target genes. (A) Circos plot of expression patterns and chromosomal positions of circRNAs. The length of the line indicates the relative size of the fold-change. Red lines indicate upregulated circRNAs, and blue lines indicate downregulated circRNAs. Green lines represent the position of circRNAs on chromosomes. (B) Heat map of differentially expressed circRNAs. (C) Volcano plot of differentially expressed circRNAs in AD-EVs compared with NC-EVs. (D) GO and KEGG pathway analysis of the parent genes of DEcircRNAs. (E) CeRNA networks were constructed based on circRNA-miRNA-mRNA interactions. The pink and purple nodes denote the downregulated and upregulated RNAs, respectively. The red and yellow nodes represent the upregulated and downregulated circRNAs, respectively. (F) GO enrichment of circRNA-associated ceRNA network genes.

### Differentially expressed circRNAs and functional enrichment analysis of circRNA-related target genes

All differentially expressed circRNAs were chosen to visualize their chromosomal locations and expression patterns (Fig. 4A). CircRNA expression patterns in AD- and NC-EVs were classified by unsupervised hierarchical clustering (Fig. 4B). The volcano plot manifested the statistically significant difference in expressed circRNAs between AD and NC EVs (Fig. 4C)—53 circRNAs were downregulated and 66 were upregulated (Supplementary Table 7). Information on circRNAs and how they function and regulate physiological and pathological processes remains scarce. The circRNAs are closely related to the function of the parent genes. Consequently, we performed GO and KEGG pathway analysis for the parent genes of DEcircRNAs (Fig. 4D, Supplementary Table 8). The most significantly enriched GO terms included regulation of GTPase activity, dendrite morphogenesis, nervous system development, and transport vesicle. The parent genes of DEcircRNAs were mainly involved in necroptosis and the wnt signaling pathway. miRanda software was used to predict circRNA-miRNA pairs and miRDB, miRTarBase, and TargetScan were used to predict miRNA-mRNA pairs. Only the miRNA-mRNA interactions that existed in all three databases were selected. Following that, the mRNAs that were DEmRNAs were included in the ceRNA network (Fig. 4E). The circRNA-associated-ceRNA network executes functions that are embodied in related mRNA genes. In GO analyses of these genes, some AD-associated terms were found, such as semaphoring-plexin signaling, which is involved in axon guidance and neuron projection guidance, axonogenesis, and voltage-gated calcium channel activity (Fig. 4F).

### Construction of critical co-expression modules by WGCNA

**Figure 5.**
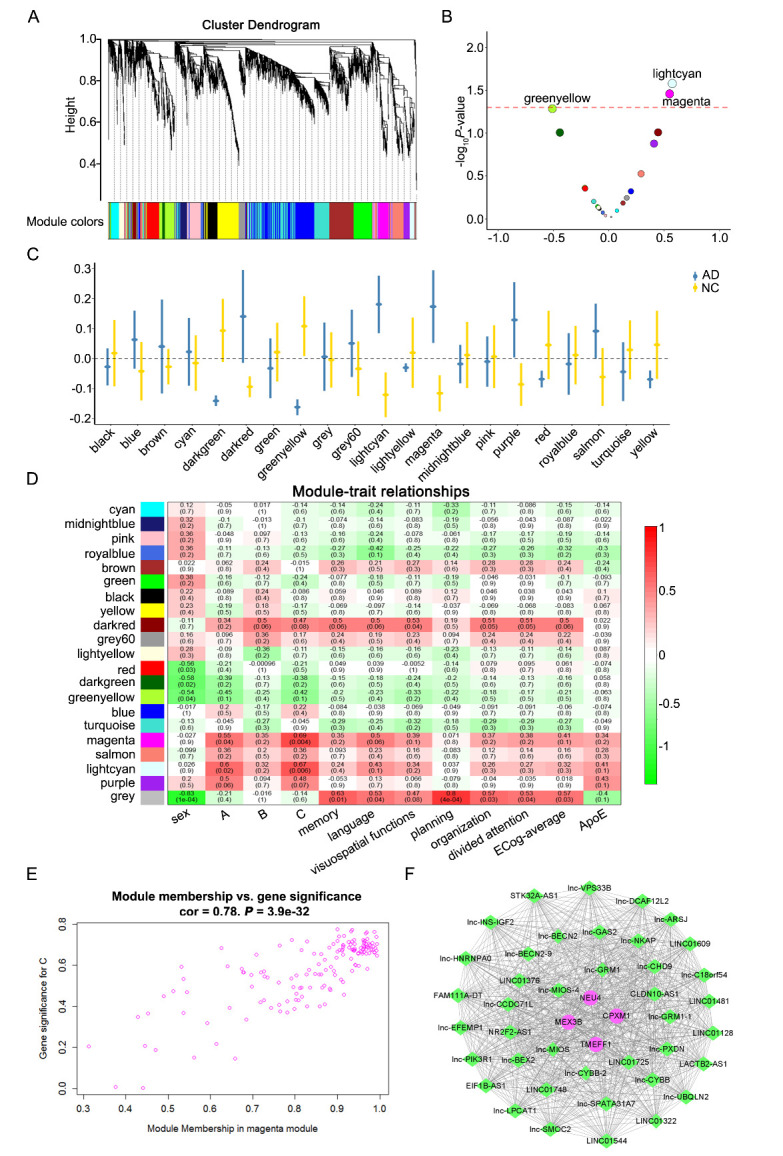
Construction of critical co-expression modules by weighted gene co-expression network analysis (WGCNA). (A) Results of WGCNA and clustering dendrograms. (B) Pearson correlation analysis of WGCNA modules assesses module correlation with AD diagnosis. (C) Module eigengenes associated with AD. (D) Pearson correlation analysis of module-trait relationships of AD based on the long RNA expression data. (E) Correlation of magenta module membership and gene significance. (F) The top 40 interactions of the genes in the magenta module.

To clarify the significant gene co-expression network involved in EVs, we performed WGCNA, as well as clustering the transcriptomes of AD and NC EVs. All of the selected genes focused on 21 modules using a topological overlap matrix (TOM)-based dissimilarity measure based on the Dynamic Tree Cut algorithm (Fig. 5A). Two modules (light cyan and magenta) were significantly associated with AD (Fig. 5B and C). We summarized the gene co-expression using eigengenes and calculated the correlation of each eigengene with clinical traits, such as sex, ABC scores [incorporating amyloid-β deposits (A), neurofibrillary tangles (NFTs) (B), and neuritic plaques (C)], ApoE genotype and the measurement of Everyday Cognition (ECog) scales including memory, language, planning, visuospatial functions, organization, and divided attention sub-items (Fig. 5D). Detailed information about the ECog scales of each donor is given in Supplementary Table 9. The module-trait relationship plot showed that the co-expression magenta module was most significantly positively associated with neuritic plaques (R = 0.69, *P* = 0.004). The co-expression light cyan module was most evidently positively associated with amyloid-β deposits (R = 0.6, *P* = 0.02). After comparing the relationships between module membership and gene significance, we considered the magenta module to be the key module associated with neuritic plaques (cor = 0.78, *P* = 3.9e-32; Fig. 5E). Thus, the magenta module was further analyzed. The magenta module contained 150 genes, including four mRNAs, five circRNAs, and 141 lncRNAs (Supplementary Table 10). The top 40 interactions of the genes in the magenta module are shown in Fig. 5F. The majority of the genes in this network have not been reported in AD research.

### RT-qPCR validation

To validate the reliability of our RNA-Seq data, we selected three key DEmRNAs (NCOR2, ERBB3, and NOTCH1), three key DElncRNAs (NEAT1, KCNQ1OT1, and FGD5-AS1), and three key DEcircRNAs (hsa_circ_0087493, hsa_circ_0102923, and hsa_circ_0089902) for RT-qPCR analysis on AD-EVs and NC-EVs. The qPCR results tallied with the RNA-Seq data (Supplementary Fig. 3), indicating that our RNA-Seq data were reliable.

### EVs alter microglial cytokine expression, phagocytosis, and calcium signaling

Based on the WGCNA results, we suspected that human brain tissue EVs may affect the formation of amyloid-β deposits and neuritic plaques. Moreover, in the functional analysis results for DElncRNAs, phagocytosis-related terms were enriched. Microglia are a type of phagocytic cell and are believed to be responsible for the regulation of amyloid-β deposits and neuritic plaque formation [30]. Thus, we hypothesized that EVs affect the function of microglia, which was followed by an investigation of the functional effects of EVs on microglia *in vitro*.

**Figure 6.**
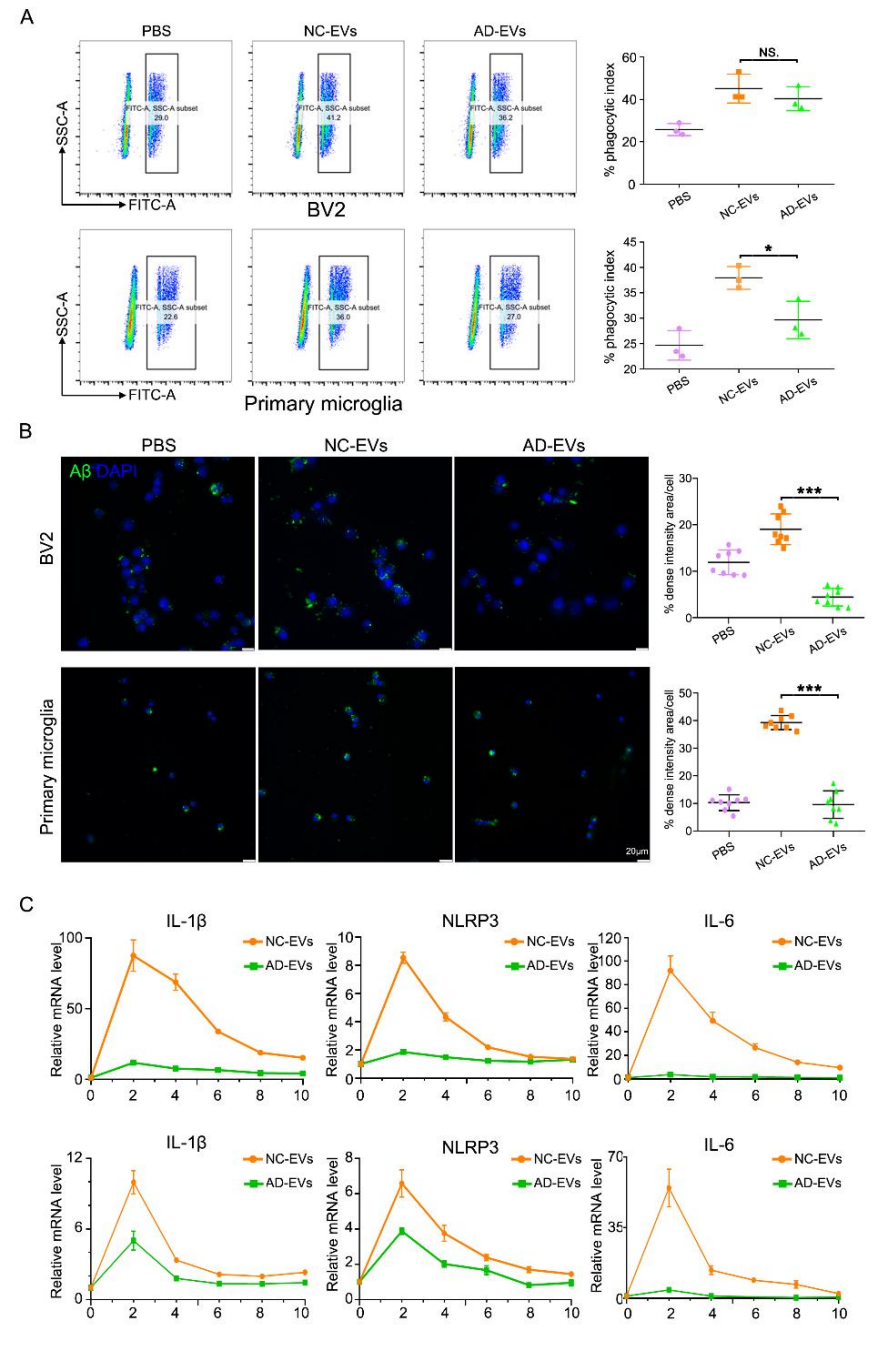
EVs alter microglial cytokine expression, phagocytosis, and calcium signaling. (A) Phagocytosis of fluorescent beads in BV2 cells (N = 3) and primary microglia cell (N = 3) after incubation with AD-EVs or NC-EVs, determined by flow cytometry. *P*-values were measured using a non-parametric test (*, *P* < 0.05). (B) Carboxyfluorescein (FAM)-Aβ levels in BV2 (N = 8) or primary microglia cells (N = 8) after incubation with AD-EVs or NC-EVs, determined by confocal microscopy. *P*-values were determined using one-way analysis of variance (***, *P* < 0.001). (C) Interleukin (IL)-1β, NLRP3, and IL-6 expression as determined by quantitative real-time-PCR in BV2 (N = 9) and primary microglia cells (N = 9) after incubation with AD-EVs or NC-EVs. *P*-values were measured using a T-test. Upper: BV2 cells; lower: primary microglia.

The pooled EVs were labeled with PKH26 dye and incubated with microglia for 24 h. Evaluation by image analysis revealed that EVs were internalized in the cells (Supplementary Fig. 2A). We used flow cytometry to assess the phagocytic ability of BV2 and primary microglia cells toward fluorescent beads. NC-EVs were more effective than AD-EVs in promoting the phagocytosis of fluorescent beads by primary microglia. Although there was no statistical difference for BV2 cells, they displayed a similar trend to that for primary microglia (Fig. 6A). To further confirm the regulatory effect of EVs on microglial phagocytosis, we determined whether EVs could modulate phagocytic uptake of Aβ. We found that compared with AD-EVs, NC-EVs showed significantly promoted Aβ uptake in BV2 cells and primary microglia (Fig. 6B). Moreover, we observed that EVs increased expression of proinflammatory cytokines such as interleukin (IL)-1β, IL-6, and NLRP3. The NC-EV-induced effects on cytokine production were stronger than those of AD-EVs in BV2 cells and primary microglia (Fig. 6C).

In our functional and pathway enrichment analysis, metal ion and calcium ion-related terms were enriched. Microglial function, including phagocytosis and cytokine synthesis, is orchestrated through highly coupled signaling pathways that depend on calcium [31]. Thus, next, we determined the effects of EVs on the intracellular calcium level in BV2 cells. At 2 h post-incubation with EVs, we found that NC-EV treatment led to an elevation of basal calcium in BV2 cells, whereas no obvious change was observed in the AD-EV treatment group (Supplementary Fig. 2B). This result is consistent with our finding that, compared with NC-EVs, the calcium signaling pathway was downregulated in AD-EVs.

## DISCUSSION

In the last 10 years, the discovery of protein and nucleic acid carriers associated with EVs has identified a role for EVs in intercellular communication. Few studies have investigated the dynamic changes in long RNA expression within EVs. In this study, we comprehensively detected the expression profiles of mRNAs, lncRNAs, and circRNAs in EVs from postmortem brain tissue of AD and NC cases. The key mRNAs, lncRNAs, and circRNAs for the pathogenesis of AD are listed in Supplementary Table 11.

Function enrichment analysis showed that the DEmRNAs were mainly involved in axon guidance, axonogenesis, ion channel activity, and calcium ion transporter activity. In addition, GSEA analysis identified several pathways relevant to AD, including axon guidance, calcium signaling, and inflammatory mediator regulation of TRP channels. Axon guidance is one of the necessary processes for neural circuit formation [32]. In fact, it has been reported that AD is caused by the disintegration of neural circuits [33]. In this study, we found that most of the mRNAs associated with axon guidance were downregulated in AD-EVs compared with NC-EVs. We speculate that AD-EVs may be involved in the disintegration process of neural circuits. Future functional and mechanistic studies are warranted to determine the role of AD-EVs in neural circuit disintegration. Cellular calcium homeostasis plays a key regulatory role in neuronal growth, action potential properties, and synaptic plasticity [34]. The accumulated evidence indicates that calcium signaling disruption is ubiquitously involved in AD pathologies [35]. Consistently, we discovered that the calcium signaling pathway was downregulated in AD-EVs compared with NC-EVs. Moreover, Hoffmann *et al*. reported that lipopolysaccharide used as a tool to activate cultured microglia could lead to a chronic elevation of the basal calcium ion level [36]. Interestingly, we found that NC-EVs were more capable of upregulating the basal calcium ion level and cytokine expression in microglia than AD-EVs. It therefore appears that calcium signals may be involved in mediating the EV-induced activation of microglia. TRP channels are non-selective calcium ion-permeable channels. Recent evidence indicates that the dysfunction of TRP channels is a missing link between calcium signaling disruption and neuronal loss in AD [37]. However, there is limited experimental data that directly demonstrates the pathological roles of TRP channels in microglia.

Network analysis revealed several hub genes, including CREBBP, RELA, and PDGFRB. A study integrated 20 transcriptome datasets and concluded that CREBBP was a key regulatory molecule in AD [22]. Our data support this claim. But, so far, the function of CREBBP in AD has seldom been studied. RELA, a subunit of NF-κB that regulates neuroinflammation, has been well-established [38]. We found that RELA was downregulated in AD-EVs compared with NC-EVs. NC-EVs more strongly promoted the expression of inflammatory factors in microglia than AD-EVs. RELA may be involved in mediation of the regulation of the inflammatory response of microglia by EVs. PDGFRB is a pericyte marker and deficient PDGFRB signaling is a prominent feature of the blood-brain barrier breakdown described in Alzheimer’s disease [39]. There was no pathological information related to the blood-brain barrier for our included AD cases. Further research is required to determine whether the downregulation of PDGFRB in AD-EVs is correlated with the destruction of the blood-brain barrier.

LncRNAs are presumed to participate in a handful of human diseases including AD [9]. The expression pattern of lncRNAs in human brain tissue EVs remains largely unexplored. We first compared differential lncRNA expression profiles of human brain tissue EVs in individuals with AD and NCs. Subsequently, we performed GO enrichment and KEGG analyses of coding genes near to the DElncRNAs. We thus identified genes that participate in AD-related terms, including metal ion transport, calcium ion homeostasis, neuron spine, and neuronal cell body. These results are similar to the findings for the enrichment terms for the DEmRNAs. The ceRNA mechanisms theory has mainly been studied in the context of cancer research [40]. We constructed a lncRNA-miRNA-mRNA network to identify ceRNA interactions, further extracting the hub network including NEAT1, miR-24, miR-17, and KCNQ1OT1. In this network, lncRNA-NEAT1 had the most interactions. lncRNA-NEAT1, a lncRNA with various functions, has been implicated in AD pathogenesis [41]. Wang *et al*. reported that downregulation of lncRNA-NEAT1 decreases Aβ clearance in neuroglial cells [42]. In parallel, our data revealed that lncRNA-NEAT1 was downregulated in AD-EVs compared with NC-EVs, and the phagocytic ability of microglia decreased after treatment with AD-EVs. Such evidence points to a conclusion that lncRNA-NEAT1 may be critical to regulating the process of Aβ clearance in AD.

Additionally, the hub gene miR-24, which can bind with lncRNA-NEAT1, was found to regulate cognitive impairment and is considered to be a prognostic marker of AD [43, 44]. However, the functional role of the lncRNA-NEAT1/miR-24 regulated-axis has not been reported in AD. miR-17 is another microRNA that binds to lncRNA-NEAT1. One study reported that miR-17 was elevated in human AD microglia whilst inhibiting elevated miR-17 in mouse microglia improved Aβ degradation [45]. It is plausible that miR-17 may mediate the regulatory effect of lncRNA-NEAT1 on Aβ clearance in microglia. Liu *et al*. found that KCNQ1OT1 enhanced microglial inflammation [28]. This is consistent with our data that KCNQ1OT1 was downregulated in AD-EVs compared with NC-EVs, and that the cytokine expression in microglia was decreased after treatment with AD-EVs.

CircRNAs are significantly enriched in the human brain [46]. The most important function of circRNAs is to act as miRNA sponges. To cite one notable example, the circRNA CDR1as has been considered a key risk factor correlated with AD. CDR1as sponges quench the normal function of miR-7 in the human brain. CDR1as can lead to a decrease in miR-7 expression, which upregulates the activity of UBE2A, a crucial AD target [46]. In this study, we systematically investigated circRNA profiles in human brain-derived EVs. Functional analysis of parent genes of DEcircRNAs showed that many neuro-related terms, including neurogenesis and synapse organization, were enriched among. Furthermore, GTPase activity related terms were also enriched. Rab GTPases are key regulators of membrane trafficking and are associated with neurodegeneration [47]. Thus, there is a possibility that circRNAs in EVs affect AD pathology by regulating membrane trafficking. Based on the ‘sponge’ theory, we predicted that DEcircRNAs targeted miRNAs, and, thus, constructed circRNA-associated-ceRNA networks. GO analyses showed that the pathological process of AD may be regulated by these networks in different ways, including effects on axon guidance, axonogenesis, ion transmembrane transport, and calcium channel activity.

This corresponds to the enrichment results for DEmRNAs and DElncRNAs, and we suggest that brain tissue-derived EVs may regulate the pathological process of AD by affecting these factors.

By performing WGCNA, we identified two modules related to AD traits, including amyloid-β deposits and neuritic plaques. As a result, we suggest that EVs may regulate the formation of amyloid-β deposits and neuritic plaques. Over and above this, we found that NC-EVs were more effective than AD-EVs in promoting cytokine expression and phagocytosis, and in upregulating calcium signaling, in microglia. The most commonly accepted view is that in AD, Aβ is the primary driver of the activation of microglia. Aβ-activated microglia migrate to plaques and phagocytose Aβ in the early stage of AD [48]. However, prolonged activation of microglia results in a decrease of Aβ clearance and sustained production of proinflammatory cytokines, which begins to damage neurons [49, 50]. In this study, an ‘ABC’ dementia score of ‘H’ or ‘I’ was an inclusion criterion for the AD group, which means that these patients were already in the late stage of AD. Based on our data, we hypothesize that AD-EVs may play a role in a feedback loop to avoid excessive activation of microglia in the late stage of AD. The functional mechanism of AD-EVs in microglia needs further research.

Our research has some limitations. Because human brain tissues are not easy to access, we used a total of 18 human brain tissue samples in this study. We performed a power calculation, and the power value was computed as 0.395. Thus, one limitation of this study is the relatively low power due to the small sample size. Further studies with larger sample sizes are required to confirm the findings of this study. In addition, because the available amount of brain tissue-derived EVs is very low, we cannot distinguish EVs secreted by different cells. Regardless, we elucidated the transcriptomic characteristics of brain tissue-derived EVs in NC and AD cases. Our findings expand knowledge of the regulatory roles of EVs in AD pathogenesis.

## Supplementary Materials

The Supplementary data can be found online at: www.aginganddisease.org/EN/10.14336/AD.2022.0607.
