# Supplementary material for: Long RNA Profiles of Human Brain Extracellular Vesicles Provide New Insights into the Pathogenesis of Alzheimer’s Disease
Source: Aging Dis. 2023 Feb 1;14(1):229–44. doi: 10.14336/AD.2022.0607 (PMC9937700; doi:10.14336/AD.2022.0607)
Supplement: Supplementary file 1 — The Supplementary data can be found online at: www.aginganddisease.org/EN/10.14336/AD.2022.0607. [file AD-14-1-229-s.pdf]

SUPPLEMENTARY DATA

Long RNA Profiles of Human Brain Extracellular Vesicles Provide New Insights into the Pathogenesis of Alzheimer's Disease

Dan Luo[1#], Haotian Liu[2#], Hanyou Liu[2], Wei Wu[1], Hanyang Zhu[2], Wei Ge[2,*], Chao Ma[1,*]

© 2022. Luo D et al. Published online at http://www.aginganddisease.org/EN/10.14336/AD.2022.0607

SUPPLEMENTARY DATA

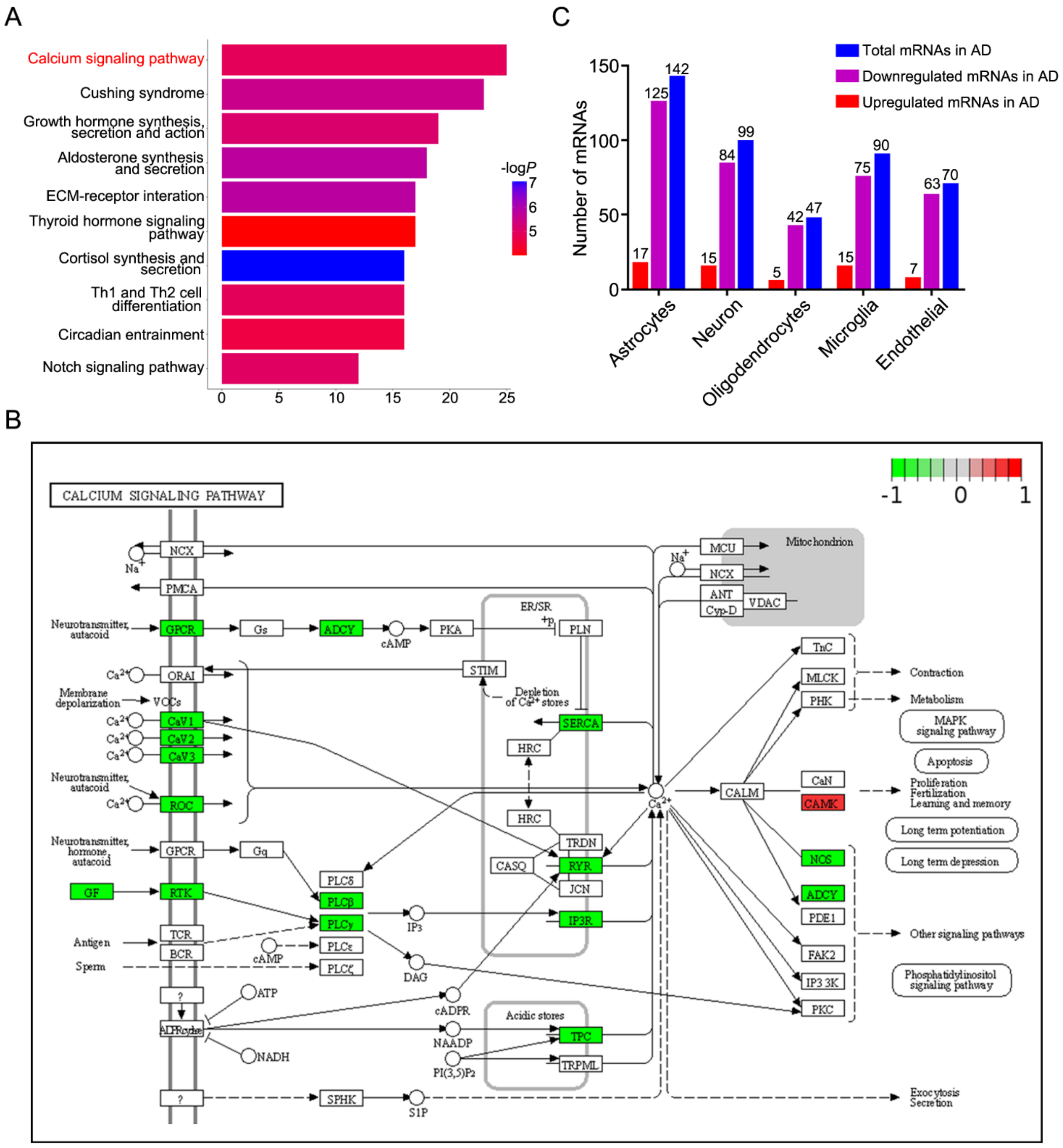

Supplementary Figure 1. (A) Signaling pathway analysis of mRNAs differentially expressed in AD-EVs compared with NC-EVs. **(B)** Mapping of DEmRNAs to the calcium signaling pathway. **(C)** Comparison of cell type-specific mRNAs in AD-EVs and NC-EVs. A red bar indicates higher expression in AD-EVs. A pink bar indicates higher expression in NC-EVs. The blue bar indicates total DEmRNAs.

© 2022. Luo D et al. Published online at http://www.aginganddisease.org/EN/10.14336/AD.2022.0607

SUPPLEMENTARY DATA

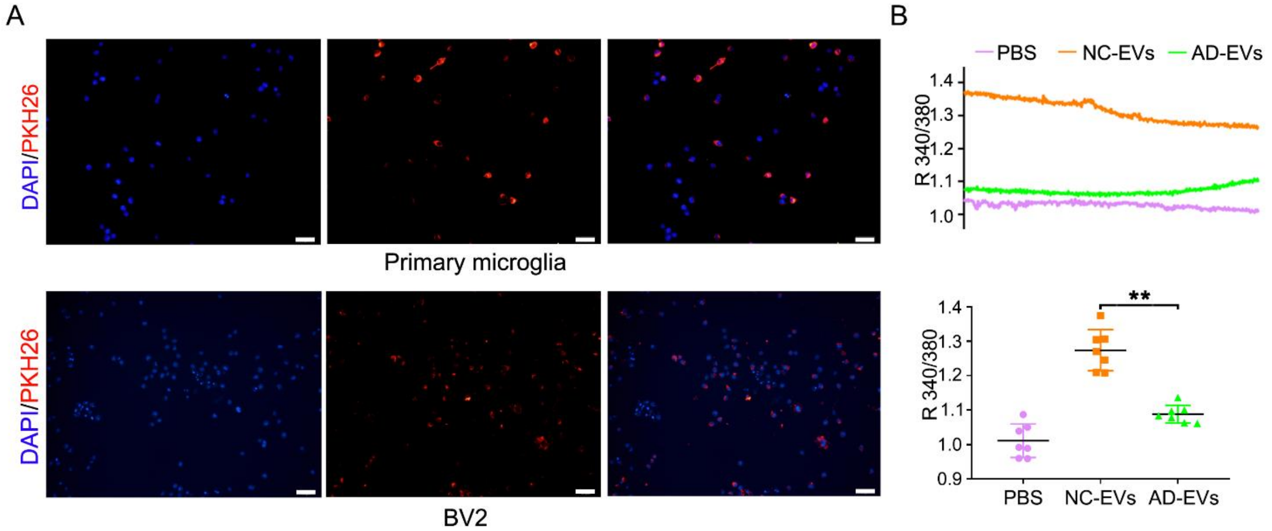

Supplementary Figure 2. (A) Pooled EVs were labeled with PKH26 dye and internalized in microglial cells. **(B)** BV2 cell calcium signaling (N = 7) was compared following treatment with AD-EVs and NC-EVs. One-way ANOVA was used to test statistical differences (**, $P < 0.01$).

© 2022. Luo D et al. Published online at http://www.aginganddisease.org/EN/10.14336/AD.2022.0607

SUPPLEMENTARY DATA

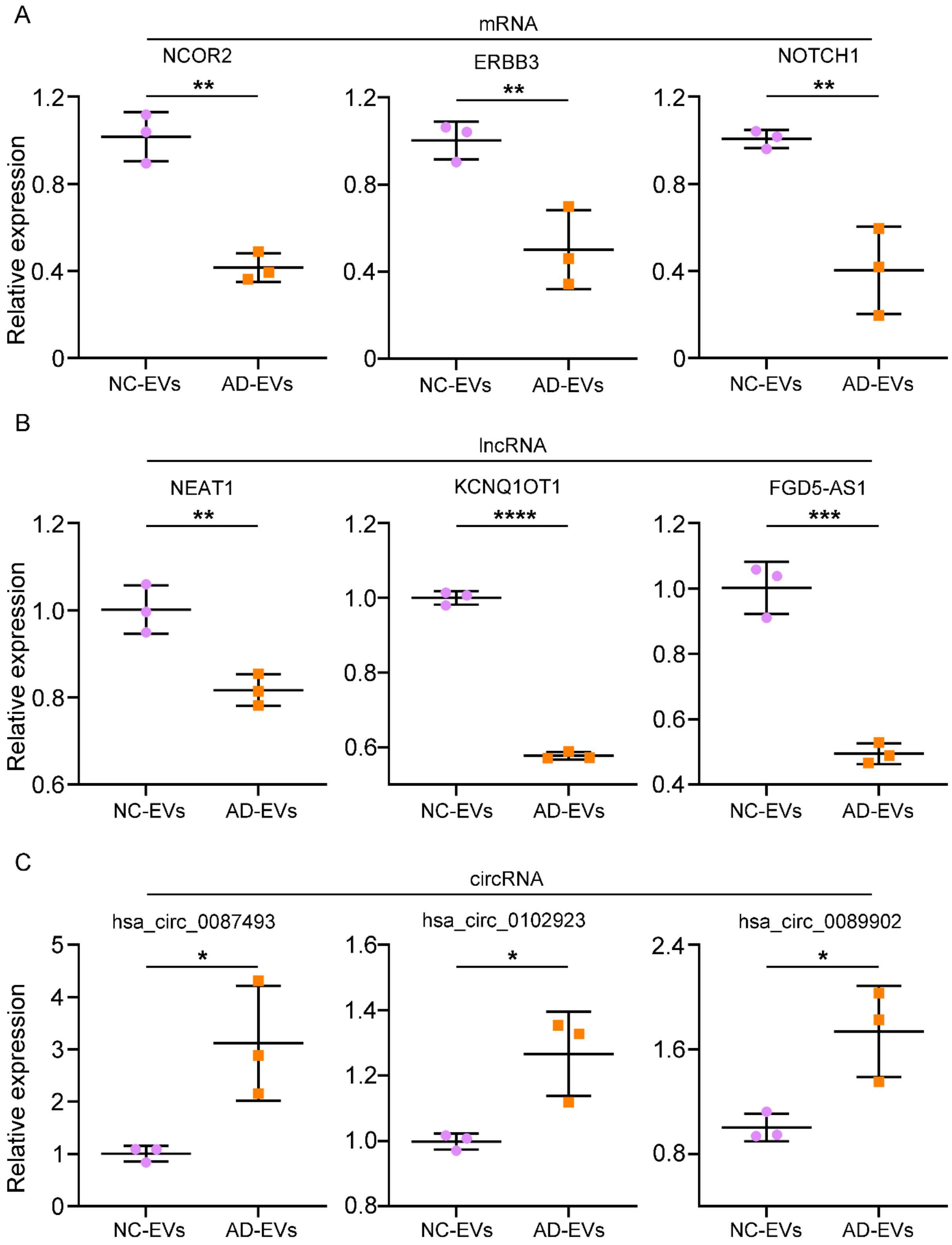

Supplementary Figure 3. The expression of three randomly selected key DEmRNAs (N = 9) **(A)**, three randomly selected key DElncRNAs (N = 9) **(B)**, and three randomly selected key DEcircRNAs (N = 9) **(C)** from the RNA-Seq dataset validated by qPCR. *P*-values were measured using T-tests (*****, *P* < 0.05; ******, *P* < 0.01; *******, *P* < 0.001; ********, *P* < 0.0001).

© 2022. Luo D et al. Published online at http://www.aginganddisease.org/EN/10.14336/AD.2022.0607

SUPPLEMENTARY DATA

Supplementary Table 1. Primers used for qRT-PCR.

Gene type	Gene symbol/ circBase ID	Forward/Reverse	Primer Sequence (5'-3')
mRNA	GAPDH	F	GTCTCCTCTGACTTCAACAGCG
		R	ACCACCCTGTTGCTGTAGCCAA
	IL-1β	F	TGGACCTTCCAGGATGAGGACA
		R	GTTCATCTCGGAGCCTGTAGTG
	NLRP3	F	TCACAACTCGCCCAAGGAGGAA
		R	AAGAGACCACGGCAGAAGCTAG
	IL-6	F	TACCACTTCACAAGTCGGAGGC
		R	CTGCAAGTGCATCATCGTTGTTC
	NCOR2	F	CCACGTCATCTACGAAGGCAAG
		R	CCTCCATCATGTCATAGGTGCG
	ERBB3	F	CTATGAGGCGATACTTGGAACGG
		R	GCACAGTTCCAAAGACACCCGA
	NOTCH1	F	GGTGAACTGCTCTGAGGAGATC
		R	GGATTGCAGTCGTCCACGTTGA
lncRNA	KCNQ1OT1	F	TGCAGAAGACAGGACACTGG
		R	CTTTGGTGGGAAAGGACAGA
	NEAT1	F	CCTAGCATGTTTGACAGGCG
		R	TGCCACCTGGAAAATAAAGCG
	FGD5-AS1	F	TGCTGCCCATCTGATCCACTACA
		R	CGCAGTCAGGTGTTGTCGTGGAG
circRNA	U6	F	CGCTTCGGCAGCACATATAC
		R	AAATATGGAACGCTTCACGA
	hsa_circ_0087493	F	TTACAGACCGGTGGATCCTG
		R	TGGTGTGACTCGAAGTTGGA
	hsa_circ_0102923	F	CTCCTGGGACTTTAGCACGT
		R	TTGGTCCAGAAACAGCAAGC
	hsa_circ_0089902	F	CTAAGAGGAAGAGAAGCAGAGC
		R	CGAACTTGATACGCTGAGGG

Supplementary Table 2. Characterization of EVs isolated from AD and NC brain tissues.

Sample ID	EVs' RNA concentration (ng/μl)	28S/18S (rRNA ratio)	RIN	EVs passed ISEV criteria
NC 1	144	0	2.1	Y
NC 2	94	0	2.7	Y
NC 3	21.8	0	/	Y
NC 4	97.6	0	2.6	Y
NC 5	104	0	2.7	Y

© 2022. Luo D et al. Published online at http://www.aginganddisease.org/EN/10.14336/AD.2022.0607

NC 6	14.3	1	2.5	Y
NC 7	75.8	0	2.7	Y
NC 8	104	0	2.7	Y
NC 9	19	0	2	Y
NC 10	14.3	0	2.5	Y
AD 1	15.8	0.2	2.7	Y
AD 2	15.8	0	1.1	Y
AD 3	37.2	0	2.4	Y
AD 4	15.1	0	2.4	Y
AD 5	52.4	0	2.6	Y
AD 6	67.6	0	2.8	Y
AD 7	13	0	2.4	Y
AD 8	96.8	0	2.7	Y

Supplementary Table 3. Differentially expressed mRNAs in AD and NC EVs from the post-mortem frontal cortex.

SYMBOL	ENTREZID	Fold Change (FC)	log_2FC	*P*-Value
HLA-DRB5	3127	4.403935483	2.138793334	0.0201624
CHI3L2	1117	4.175591017	2.061980412	0.0024189
CHI3L1	1116	3.788855152	1.921761987	0.0458164
SCG2	7857	2.955950711	1.563622214	0.0285952
SOD2	6648	2.930000992	1.550901153	0.0082466
PGAM2	5224	2.461350343	1.299450022	0.0460797
MAP1LC3C	440738	2.414592375	1.271779658	0.0069509
LINC01503	100506119	2.412737405	1.270670906	0.0467203
GAP43	2596	2.386259725	1.254751077	0.0139555
CRABP1	1381	2.342154247	1.227836091	0.0273774
GBP2	2634	2.280361889	1.189262795	0.0308177
SLC39A4	55630	2.271730006	1.183791381	0.0226616
BCYRN1	618	2.250615363	1.170319517	0.0028056
CITED1	4435	2.193056745	1.132943142	0.0407376
CNTN6	27255	2.159793533	1.110893403	0.0352364
AKR1C1	1645	2.146567076	1.102031255	0.0037063
ADH1B	125	2.135037022	1.094261087	0.0294276
PLIN2	123	2.131951907	1.092174894	0.0274063
TMEM220	388335	2.126771801	1.088665243	0.0003045
KRT19	3880	2.124271616	1.086968246	0.0201415
SRPX2	27286	2.09964449	1.070145072	0.0439975
SLC7A7	9056	2.086048673	1.06077282	0.0355942
GPD1	2819	2.083328961	1.058890661	0.0274404
PDPN	10630	2.082178677	1.058093875	0.0148707
MGST1	4257	2.070067056	1.049677502	0.0205901
NCF2	4688	1.988266564	0.99151119	0.0169021

© 2022. Luo D et al. Published online at http://www.aginganddisease.org/EN/10.14336/AD.2022.0607

RGS10	6001	1.981004935	0.986232474	0.0240659
SAT1	6303	1.979946369	0.985461352	0.0175101
ZNF208	7757	1.974054194	0.981161597	0.0225137
FMOD	2331	1.958858245	0.970012999	0.0491588
VIM	7431	1.949447073	0.963064987	0.0046878
DYRK3	8444	1.946541021	0.960912748	0.0290387
RGR	5995	1.946268196	0.960710528	0.0233798
LYL1	4066	1.923734282	0.943909539	0.0483591
LINC00924	145820	1.886768386	0.915917333	0.0286354
MXD1	4084	1.879560468	0.910395329	0.0174363
CFI	3426	1.856964006	0.892945851	0.0374154
KCNK2	3776	1.855446479	0.891766387	0.0116822
INTS4P2	644619	1.848400683	0.886277528	0.0012873
FNDC9	408263	1.838696924	0.878683697	0.0246146
RN7SK	125050	1.830548174	0.872275741	1.16E-05
HPGDS	27306	1.821218503	0.864904022	0.0266971
NSG1	27065	1.821150743	0.864850344	0.00144
LRRC75B	388886	1.819047165	0.86318295	0.0253878
GCHFR	2644	1.79881802	0.847049241	0.0235287
PDLIM1	9124	1.781603231	0.83317608	0.0455278
TMEM45A	55076	1.766355303	0.82077557	0.0463542
PAPPA2	60676	1.750807312	0.808020314	0.0020565
ADM	133	1.747175584	0.805024601	0.0472749
S100A6	6277	1.743610277	0.802077612	0.0355382
PACRG	135138	1.730080023	0.79083877	0.0165162
MDP1	145553	1.727600163	0.788769357	0.0022169
EMP3	2014	1.707294363	0.771711822	0.0260743
RCAN1	1827	1.688173821	0.755463457	0.0488238
NUPR1	26471	1.687585155	0.754960302	0.0114252
MIR100HG	399959	1.686761802	0.754256257	0.0387733
ZNF773	374928	1.683058684	0.75108548	0.0381184
ZNF286B	729288	1.678392556	0.747080184	0.0096179
AGBL4	84871	1.671900229	0.741488756	0.0409434
GEM	2669	1.668328666	0.738403532	0.035442
SNCAIP	9627	1.658759024	0.730104315	0.0142488
LIN7B	64130	1.6524947	0.724645645	0.026602
ZFP69B	65243	1.65231763	0.724491047	0.0267273
ZFAND2A	90637	1.650404498	0.722819658	0.0197597
SNHG1	23642	1.64829451	0.72097404	0.0465926
PBLD	64081	1.643888739	0.717112659	0.0133384
DNAH7	56171	1.643704705	0.716951139	0.0108969
FAM122C	159091	1.643302893	0.716598422	0.0293862
GAPDH	2597	1.643210864	0.716517625	0.0269272
GLIPR2	152007	1.640994859	0.714570719	0.0378488
GFAP	2670	1.629250503	0.70420844	0.0116298
NOX4	50507	1.627629206	0.702772073	0.0464288

SUPPLEMENTARY DATA

COX10-AS1	100874058	1.625391335	0.700787108	0.0494564
CLU	1191	1.619013226	0.695114771	0.00736
BLVRB	645	1.616935548	0.693262174	0.0021065
NDUFB1	4707	1.61643333	0.692814004	0.0034368
ITM2C	81618	1.616014809	0.692440419	0.0006134
SAT2	112483	1.615054212	0.691582592	0.0014587
CLHC1	130162	1.61487255	0.691420308	0.0162799
IL1R1	3554	1.608309112	0.685544714	0.033379
MMACHC	25974	1.604514739	0.682137042	0.044736
TMEM99	147184	1.591295694	0.670201941	0.0051162
FHIT	2272	1.590116587	0.669132547	0.0263542
RMND1	55005	1.585011779	0.664493562	0.0079737
C2orf74	339804	1.578062333	0.658154193	0.0285678
MAP2K6	5608	1.566490616	0.647536127	0.0174353
FABP7	2173	1.566473239	0.647520123	0.0037724
ATOX1	475	1.556989493	0.638759208	0.0110749
GYPC	2995	1.552674527	0.634755443	0.0419094
SPAG7	9552	1.552278505	0.634387424	0.0175558
EIF1B	10289	1.540630506	0.623520897	0.0464873
IMPDH2	3615	1.534941934	0.61818408	0.0332341
NT5C	30833	1.533770217	0.617082361	0.0478453
HAUS4	54930	1.533176609	0.616523893	0.0057171
LGALS1	3956	1.533159505	0.616507798	0.019123
YIPF1	54432	1.532135054	0.615543473	0.0371154
CAMK1	8536	1.529615758	0.613169292	0.0238423
MPV17	4358	1.529446552	0.613009691	0.0035589
DBI	1622	1.529081025	0.612664856	0.0385157
THOC3	84321	1.527419816	0.611096646	0.0305975
BHMT2	23743	1.526476645	0.610205516	0.0132282
FAHD1	81889	1.522897376	0.606818726	0.006704
MAOB	4129	1.521343033	0.605345489	0.0253323
SOCS2	8835	1.520945759	0.604968704	0.0257955
CD99	4267	1.520758244	0.604790825	0.043336
CITED2	10370	1.514141197	0.598499746	0.0487165
PID1	55022	1.511920707	0.59638248	0.0005261
AQP4	361	1.509130121	0.593717204	0.0250808
ATP1B2	482	1.508885175	0.593483022	0.049921
SMIM8	57150	1.507215286	0.591885502	0.0176229
PIFO	128344	1.504456201	0.589242106	0.0314401
TMEM147	10430	1.501892992	0.586782026	0.0164122
LRMP	4033	1.500291828	0.585243153	0.0398645
WTIP	126374	0.665687259	-0.587083538	0.0479914
RGS5	8490	0.665343846	-0.587827985	0.0116809
POLR1A	25885	0.665284362	-0.587956972	0.0362248
VPS33B	26276	0.665190757	-0.588159973	0.0106936
SH3PXD2B	285590	0.664981186	-0.588614572	0.0331609

© 2022. Luo D et al. Published online at http://www.aginganddisease.org/EN/10.14336/AD.2022.0607

SUPPLEMENTARY DATA

AKAP1	8165	0.664968005	-0.588643169	0.0028984
SOX12	6666	0.664938891	-0.588706334	0.0436479
MDN1	23195	0.664749227	-0.589117901	0.0165996
GPATCH8	23131	0.66385368	-0.591062802	0.0050403
RAVER1	125950	0.663038707	-0.592835	0.0341901
KIAA0754	643314	0.66243474	-0.59414976	0.0141792
SLC2A1	6513	0.662360743	-0.594310926	0.0408605
PIGG	54872	0.662096777	-0.594885988	0.0288578
PLCG1	5335	0.66194917	-0.595207655	0.0403461
SEC24C	9632	0.660521623	-0.598322304	0.0172258
LEMD2	221496	0.660467347	-0.598440858	0.0089627
RELA	5970	0.659405646	-0.600761856	0.0189265
ZFP36L2	678	0.659020423	-0.60160492	0.0228221
ANKRD11	29123	0.658564181	-0.602604048	0.0395524
VPS37B	79720	0.658444051	-0.602867237	0.0043566
HERC2	8924	0.658347888	-0.603077951	0.0179097
MYO18A	399687	0.658211738	-0.603376341	0.0423912
MTA1	9112	0.657548039	-0.604831796	0.0124704
INTS3	65123	0.656772726	-0.606533877	0.0187661
TTC28	23331	0.656490816	-0.607153267	0.0033488
FMNL3	91010	0.656386923	-0.607381599	0.0086627
SIPA1L3	23094	0.656318119	-0.607532833	0.0381877
HDAC6	10013	0.656031957	-0.608162002	0.0133692
AARS2	57505	0.655881438	-0.608493049	0.0447492
NELFA	7469	0.655101791	-0.610209001	0.0348242
TANC2	26115	0.654978965	-0.61047952	0.0202344
ZNF142	7701	0.654088205	-0.612442895	0.0298497
NKTR	4820	0.653912962	-0.612829474	0.0118282
TTI1	9675	0.65388228	-0.612897167	0.0320768
GCN1	10985	0.653758405	-0.613170506	0.0451085
PLCG2	5336	0.653656734	-0.613394887	0.0376666
NDST1	3340	0.653305024	-0.614171361	0.0206432
GAK	2580	0.652590882	-0.615749265	0.0489524
TM9SF4	9777	0.651941688	-0.617185163	0.029445
MEN1	4221	0.651774067	-0.617556144	0.0382565
SPEN	23013	0.651285969	-0.618636948	0.0106037
B3GNT2	10678	0.651246883	-0.618723532	0.0478907
IMPDH1	3614	0.650889885	-0.619514601	0.0435587
NBPF12	149013	0.650546555	-0.620275791	0.0022331
RREB1	6239	0.650388971	-0.620625302	0.0046942
RRP9	9136	0.65026519	-0.6208999	0.036557
SYMPK	8189	0.650155806	-0.621142602	0.0236256
LNX2	222484	0.650005932	-0.621475211	0.0164561
SFI1	9814	0.64759734	-0.626831036	0.0285965
CREBBP	1387	0.647054684	-0.628040452	0.0017659
KLHL26	55295	0.646731264	-0.628761741	0.0397291

© 2022. Luo D et al. Published online at http://www.aginganddisease.org/EN/10.14336/AD.2022.0607

WASH2P	375260	0.646648218	-0.628947007	0.0293684
ASXL1	171023	0.646592917	-0.629070391	0.0347636
WDR24	84219	0.646097556	-0.630176077	0.0451621
ZNF703	80139	0.645975181	-0.630449359	0.0107376
DIS3L2	129563	0.644841678	-0.632983102	0.0257209
GRID1	2894	0.644036592	-0.634785435	0.0299732
IFT172	26160	0.64390811	-0.635073275	0.0241915
DDX27	55661	0.643727408	-0.635478198	0.0444436
KDM4B	23030	0.64358312	-0.635801607	0.0177797
RBM19	9904	0.643573494	-0.635823185	0.0358278
BRF1	2972	0.643497673	-0.635993164	0.028373
ZNF526	116115	0.643334036	-0.636360078	0.0367152
IER2	9592	0.64326826	-0.63650759	0.0129645
COL5A2	1290	0.6428692	-0.637402863	0.0305261
TP73-AS1	57212	0.642223792	-0.638851982	0.0253045
LAMA3	3909	0.642146144	-0.639026421	0.0307709
ANKS1A	23294	0.64098289	-0.641642248	0.0154251
FUT4	2526	0.640166561	-0.643480776	0.0278268
ULK3	25989	0.638574094	-0.647074069	0.0319138
RRBP1	6238	0.637273685	-0.650015007	0.0172368
RNPEPL1	57140	0.637144577	-0.650307318	0.0163967
SUFU	51684	0.636758543	-0.651181684	0.0168483
SOX13	9580	0.635740318	-0.65349051	0.0113741
C7orf26	79034	0.635626504	-0.653748812	0.0499628
LZTR1	8216	0.63470429	-0.655843502	0.0396185
PLXND1	23129	0.634494245	-0.656321017	0.0226903
DVL2	1856	0.63423598	-0.656908373	0.0334522
GOLGA2P7	388152	0.633288574	-0.659065044	0.0151145
CHMP6	79643	0.632938365	-0.659863076	0.0340459
C10orf90	118611	0.632453028	-0.660969759	0.0012681
PER2	8864	0.632013508	-0.661972702	0.005237
TNFRSF14	8764	0.631976278	-0.662057688	0.0302463
SLC22A23	63027	0.631426078	-0.66331425	0.0455449
SNX33	257364	0.631056413	-0.664159116	0.0165746
TSPAN14	81619	0.630923261	-0.664463553	0.0217673
SLC37A1	54020	0.630373728	-0.665720687	0.0191709
ARID1A	8289	0.63000999	-0.66655339	0.0080977
SEMA4B	10509	0.628196996	-0.670711051	0.0265026
MOV10	4343	0.627419843	-0.672496938	0.0498024
TSPAN9	10867	0.62700626	-0.673448249	0.0424855
SAMD14	201191	0.626828055	-0.673858342	0.0191951
RPTOR	57521	0.626560077	-0.674475248	0.036345
FBRS	64319	0.626218093	-0.675262903	0.0233888
PRRC2A	7916	0.626199722	-0.675305226	0.0263882
PAQR5	54852	0.624979343	-0.678119588	0.0371581
EBF4	57593	0.624781697	-0.678575905	0.044138

© 2022. Luo D et al. Published online at http://www.aginganddisease.org/EN/10.14336/AD.2022.0607

SUPPLEMENTARY DATA

RBCK1	10616	0.624359156	-0.679551931	0.0200248
C16orf58	64755	0.624217922	-0.679878316	0.0383879
PCSK6	5046	0.622775502	-0.683215901	0.0446346
SLC10A3	8273	0.621293468	-0.686653209	0.0458833
CAPN10	11132	0.621128854	-0.687035506	0.0386799
TNR	7143	0.620529567	-0.688428141	0.0036029
RNF31	55072	0.620499408	-0.688498261	0.0102678
AGPAT2	10555	0.620083304	-0.68946605	0.044631
ZZEF1	23140	0.619563492	-0.69067596	0.0031171
ZNF587	84914	0.619546863	-0.690714683	0.0209288
CHERP	10523	0.618622967	-0.6928677	0.0467655
EVC	2121	0.617858586	-0.69465142	0.0037597
MYH9	4627	0.617847354	-0.694677646	0.0276543
LSS	4047	0.61761303	-0.695224904	0.0135073
C19orf47	126526	0.617067916	-0.69649881	0.0064964
RNF44	22838	0.616940493	-0.696796753	0.0334772
ANKRD33B	651746	0.616859042	-0.696987236	0.0179207
MED24	9862	0.616451277	-0.697941222	0.0276307
ZBTB16	7704	0.615813646	-0.699434258	0.0377868
POM121C	100101267	0.615236275	-0.700787526	0.0199852
ARHGEF10L	55160	0.613525951	-0.704803727	0.0115891
ECE1	1889	0.613490014	-0.704888234	0.0219499
HYDIN	54768	0.613427845	-0.705034439	0.0089382
DNMT3A	1788	0.612959955	-0.70613527	0.0260135
GRAMD1A	57655	0.612570758	-0.707051596	0.0299043
PCNT	5116	0.61235543	-0.707558815	0.0133734
TRMT1	55621	0.611612251	-0.709310789	0.0419822
ZC3H4	23211	0.61135686	-0.709913342	0.0074062
ZNF318	24149	0.611354572	-0.709918741	0.0136137
URGCP	55665	0.61062623	-0.711638532	0.0401107
TTLL3	26140	0.610439311	-0.712080223	0.0097732
KDM5C	8242	0.609987139	-0.71314927	0.0314098
MYOCD	93649	0.609629088	-0.713996353	0.0415199
PIK3CD	5293	0.608617771	-0.716391635	0.0129241
NINL	22981	0.607725348	-0.718508628	0.0441194
ATP8B2	57198	0.606763624	-0.720793497	0.0046943
ZNRF3	84133	0.605665905	-0.723405896	0.0016907
FOSL2	2355	0.604908496	-0.725211172	0.0382337
ZFHX3	463	0.604220452	-0.726853077	0.0076513
EFS	10278	0.604014273	-0.727345454	0.0106324
JUP	3728	0.603776799	-0.727912775	0.0378788
RASGEF1C	255426	0.603539538	-0.72847981	0.0371452
DFFB	1677	0.603356924	-0.728916395	0.0324853
PLOD1	5351	0.602131351	-0.73184986	0.0105907
RNF166	115992	0.601552826	-0.733236661	0.0238937
NFATC1	4772	0.600404921	-0.735992293	0.0242588

© 2022. Luo D et al. Published online at http://www.aginganddisease.org/EN/10.14336/AD.2022.0607

STAT6	6778	0.600301233	-0.736241464	0.0138844
FBN1	2200	0.599884884	-0.737242417	0.0041352
ATP7B	540	0.599831256	-0.737371394	0.0213335
SHROOM4	57477	0.599135861	-0.739044907	0.0152374
SIPA1	6494	0.599118829	-0.73908592	0.0337064
SMOC1	64093	0.598532927	-0.74049748	0.0083968
PRDM16	63976	0.598393581	-0.740833395	0.0141135
RANBP10	57610	0.598031897	-0.741705659	0.0069226
FKRP	79147	0.59768164	-0.74255087	0.0471389
DAPK2	23604	0.597632418	-0.742669686	0.0096375
DRP2	1821	0.597459693	-0.743086709	0.021839
POM121	9883	0.597406616	-0.74321488	0.0038158
CRTC1	23373	0.596827991	-0.744612897	0.0396259
HIP1R	9026	0.596674925	-0.744982945	0.0440652
RRP1	8568	0.596366691	-0.745728414	0.0172454
TVP23A	780776	0.595949906	-0.746737029	0.0415884
RBM33	155435	0.595844619	-0.746991934	0.0032481
ATP11A	23250	0.59549725	-0.74783325	0.0247249
BRD4	23476	0.594508511	-0.750230631	0.0222014
LOC100507412	100507412	0.5943649	-0.750579175	0.0248084
AGPAT4	56895	0.594353215	-0.750607539	0.0160594
KIF7	374654	0.59310004	-0.753652626	0.0278628
DGKD	8527	0.59237972	-0.755405843	0.0087407
SYNPO	11346	0.592004565	-0.756319795	0.0455112
BAHD1	22893	0.591986175	-0.75636461	0.0017762
RHOBTB2	23221	0.59195609	-0.756437932	0.0292252
FZD5	7855	0.591223994	-0.758223273	0.0394328
BEGAIN	57596	0.590573612	-0.759811201	0.026313
PWP2	5822	0.589231056	-0.763094625	0.0430835
ATF6B	1388	0.589101845	-0.763411024	0.0315894
RRN3P3	100131998	0.588653425	-0.764509611	0.0319199
FOXK1	221937	0.588353153	-0.765245717	0.0159767
MDC1	9656	0.587728021	-0.766779412	0.0003644
POLD1	5424	0.587298547	-0.767834027	0.0316277
THBS3	7059	0.586922207	-0.768758798	0.0490995
IFFO1	25900	0.58638183	-0.770087694	0.0448616
COX19	90639	0.585886947	-0.771305786	0.0444115
TEP1	7011	0.585823186	-0.771462802	0.0119345
HYAL2	8692	0.585793406	-0.77153614	0.0262575
RNF213	57674	0.585657317	-0.77187134	0.005482
PIGM	93183	0.58550355	-0.772250177	0.0040539
HECTD4	283450	0.584805516	-0.773971175	0.0273976
ESYT1	23344	0.584001419	-0.775956219	0.0059952
BCAM	4059	0.581615346	-0.78186276	0.0369339
DSCAM	1826	0.580715399	-0.784096805	0.0051403
TMEM184B	25829	0.580512369	-0.784601287	0.0264075

© 2022. Luo D et al. Published online at http://www.aginganddisease.org/EN/10.14336/AD.2022.0607

TMEM39B	55116	0.580003092	-0.785867504	0.0196562
CLUH	23277	0.57989253	-0.786142541	0.0181721
OTX1	5013	0.579445912	-0.787254094	0.0335229
TBX2	6909	0.578993757	-0.788380302	0.0495278
UNC80	285175	0.578899887	-0.78861422	0.0412122
ZMIZ2	83637	0.577022939	-0.793299421	0.0375041
GYS1	2997	0.576717311	-0.794063768	0.0077498
ITGA4	3676	0.575920473	-0.796058485	0.0383992
CAMTA2	23125	0.575334143	-0.797528006	0.033529
FAM78A	286336	0.575169453	-0.797941037	0.0335814
RGMA	56963	0.575045566	-0.798251816	0.0331548
MSTO1	55154	0.574988218	-0.798395699	0.0311085
SLCO3A1	28232	0.574865083	-0.79870469	0.0424707
SYN3	8224	0.574631651	-0.799290635	0.040092
SALL3	27164	0.574514297	-0.799585299	0.0396103
CTC1	80169	0.573953924	-0.80099317	0.0336889
LRP1	4035	0.573906736	-0.801111787	0.0428842
PRRC2B	84726	0.573828133	-0.801309394	0.0230484
PICK1	9463	0.573030729	-0.803315588	0.0218104
CD276	80381	0.57292602	-0.803579235	0.0310474
MAPKBP1	23005	0.57273186	-0.804068234	0.0120599
UBA7	7318	0.572066519	-0.805745183	0.0329157
DLGAP3	58512	0.571873735	-0.806231447	0.0367544
PLD6	201164	0.571521748	-0.807119696	0.0361569
NASP	4678	0.570938262	-0.808593345	0.0235812
SIDT2	51092	0.570909237	-0.808666689	0.0162845
PNPLA6	10908	0.570826349	-0.808876166	0.0447962
ERF	2077	0.570822458	-0.808885999	0.0150577
DDX56	54606	0.56956778	-0.812060557	0.0111281
SLC38A7	55238	0.568857588	-0.813860571	0.018958
SLC8B1	80024	0.568705996	-0.81424508	0.0369757
TSC2	7249	0.568666013	-0.814346513	0.0430968
ACSF3	197322	0.567409376	-0.817538106	0.0156377
GPIHBP1	338328	0.567209702	-0.818045884	0.0498661
PHF8	23133	0.566662343	-0.819438763	4.72E-05
SSC5D	284297	0.566271049	-0.820435321	0.026099
NAT10	55226	0.565699243	-0.821892855	0.0015874
ARL10	285598	0.565490028	-0.82242651	0.0321436
CD14	929	0.565114756	-0.823384233	0.037654
FBRSL1	57666	0.564722944	-0.824384847	0.0131239
DOC2B	8447	0.564722474	-0.824386048	0.0057472
EGFL7	51162	0.564377847	-0.825266735	0.0127625
MED12	9968	0.564157935	-0.825828997	0.009554
TTF2	8458	0.564016828	-0.826189888	0.0354379
RNF165	494470	0.563643229	-0.82714583	0.046862
ADCY6	112	0.563131637	-0.828455891	0.016337

© 2022. Luo D et al. Published online at http://www.aginganddisease.org/EN/10.14336/AD.2022.0607

SUPPLEMENTARY DATA

TYSND1	219743	0.562997123	-0.828800544	0.0493738
POLR2A	5430	0.562620701	-0.829765458	0.0058268
ALK	238	0.562469532	-0.830153146	0.0475488
GPSM1	26086	0.559912389	-0.836726991	0.0094011
TTC31	64427	0.559362754	-0.838143903	0.0345986
SLC31A2	1318	0.559253227	-0.838426418	0.0206467
PODXL	5420	0.558594661	-0.840126309	0.0062107
PCYOX1L	78991	0.558518183	-0.840323845	0.0365014
CACNA1C	775	0.558450437	-0.840498848	0.0285166
ITPRIP	85450	0.558021091	-0.841608444	0.0239109
MAP3K11	4296	0.557703756	-0.842429106	0.0081152
FZD7	8324	0.557374578	-0.843280893	0.0136756
DPH1	1801	0.557148907	-0.843865132	0.0152119
TEAD3	7005	0.55688786	-0.844541252	0.0111672
FBXO10	26267	0.556805117	-0.844755625	0.0241989
LINC01347	731275	0.556699193	-0.845030103	0.007006
FLNA	2316	0.556359596	-0.845910444	0.0115442
TNKS1BP1	85456	0.555783558	-0.847404939	0.0082882
COL12A1	1303	0.555396467	-0.848410095	0.0473715
ATP1A1-AS1	84852	0.555248943	-0.848793354	0.0196222
OGFR	11054	0.555177554	-0.848978854	0.0453556
SOX1	6656	0.555115762	-0.849139437	0.0313495
EPHB2	2048	0.553635525	-0.852991576	0.0458814
SEC16A	9919	0.553499126	-0.853347055	0.0084239
RECQL5	9400	0.55333515	-0.853774523	0.0176074
NCLN	56926	0.553102248	-0.85438189	0.0275191
CPB2-AS1	100509894	0.552151389	-0.856864216	0.0088982
ZNF316	100131017	0.551129758	-0.859536067	0.0118746
ADGRL1	22859	0.550930095	-0.860058822	0.0386598
BCAR1	9564	0.549933036	-0.86267214	0.0074125
CSF1	1435	0.549719956	-0.863231242	0.0367453
SLC43A2	124935	0.548919235	-0.8653342	0.0403742
ALPK3	57538	0.548710169	-0.865883782	0.0495432
UNKL	64718	0.548053317	-0.867611843	0.0270732
IRS2	8660	0.547682795	-0.868587535	0.0381145
SNRNP70	6625	0.545605187	-0.874070735	0.0298948
CCNE1	898	0.54539154	-0.874635774	0.0317468
SHC2	25759	0.545348645	-0.874749247	0.018232
JRK	8629	0.545185197	-0.875181704	0.0362592
B4GALNT4	338707	0.545154989	-0.875261645	0.0236405
ARFGAP1	55738	0.544728105	-0.87639179	0.0256112
CUL9	23113	0.544095797	-0.878067411	0.0274787
PHRF1	57661	0.543833556	-0.878762924	0.0326886
C1QTNF1	114897	0.542840623	-0.881399408	0.0244757
SEMA4G	57715	0.542591793	-0.882060869	0.0073565
FAM227A	646851	0.542398728	-0.882574299	0.0408132

© 2022. Luo D et al. Published online at http://www.aginganddisease.org/EN/10.14336/AD.2022.0607

MYO9B	4650	0.542003335	-0.883626366	0.0048529
CECR2	27443	0.54190499	-0.883888164	0.0028789
ELMSAN1	91748	0.541855578	-0.884019717	0.0020469
SPNS2	124976	0.54045342	-0.887757812	0.0363281
DHX34	9704	0.539647745	-0.8899101	0.0166924
SCARB1	949	0.53959611	-0.890048146	0.0010853
NUMA1	4926	0.539402541	-0.890565778	0.0235313
SFSWAP	6433	0.539355997	-0.89069027	0.0013733
MNT	4335	0.539157689	-0.891220811	0.0109902
MIDN	90007	0.538652146	-0.892574195	0.0055699
ADCY9	115	0.538371278	-0.89332665	0.0064308
CELF3	11189	0.537961796	-0.894424374	0.0290791
JAG1	182	0.53787644	-0.894653296	0.0019042
COL6A1	1291	0.537283659	-0.896244134	0.0469874
MARVELD1	83742	0.53713154	-0.896652657	0.0175869
GTF2IP20	441124	0.537006525	-0.896988477	0.0257065
PLEC	5339	0.536099262	-0.899427945	0.0278676
SLC52A2	79581	0.536007457	-0.899675024	0.0390356
DENND4B	9909	0.534618701	-0.90341779	0.0141022
MICALL1	85377	0.534383628	-0.904052285	0.0084089
SHANK3	85358	0.534342896	-0.904162255	0.0377987
SEMA6A	57556	0.533655697	-0.906018848	0.0018026
ADCY7	113	0.533640909	-0.906058827	0.0162367
BACH2	60468	0.533453356	-0.906565965	0.0102757
FAT4	79633	0.533325811	-0.906910944	0.0025573
SARM1	23098	0.533151693	-0.907382026	0.0241926
BMP8A	353500	0.533147917	-0.907392245	0.0212532
CIC	23152	0.531839088	-0.910938281	0.0392157
SHROOM1	134549	0.531724406	-0.911249406	0.0368521
TTC7A	57217	0.53099489	-0.913230118	0.0030429
RP9	6100	0.530848082	-0.913629046	0.0253829
FRAS1	80144	0.530628976	-0.914224635	0.0205855
SPNS1	83985	0.530371752	-0.914924155	0.0115395
GIGYF1	64599	0.529942875	-0.916091241	0.0120373
CDC42EP2	10435	0.529600354	-0.91702401	0.0399984
TNRC6C	57690	0.529238666	-0.918009627	0.0002563
TMEM200B	399474	0.528986708	-0.918696624	0.0194804
CDK18	5129	0.528897385	-0.918940252	0.0137031
ROBO4	54538	0.52859822	-0.919756527	0.0353679
RIN1	9610	0.528461069	-0.920130899	0.0050493
MAP3K14	9020	0.526703589	-0.924936806	0.009534
NEU3	10825	0.526242874	-0.926199301	0.0064833
STK10	6793	0.525688582	-0.927719694	0.007235
PCDHB16	57717	0.525278109	-0.928846634	0.0055056
LRFN4	78999	0.525050675	-0.929471424	0.0193961
ZBED4	9889	0.524799763	-0.930161027	0.0033961

© 2022. Luo D et al. Published online at http://www.aginganddisease.org/EN/10.14336/AD.2022.0607

RALGDS	5900	0.524287678	-0.931569457	0.0174743
KSR2	283455	0.524161227	-0.931917456	0.0372218
OSBP2	23762	0.524064219	-0.932184484	0.0230261
LMF2	91289	0.523783938	-0.932956276	0.0327189
MGRN1	23295	0.523152414	-0.934696776	0.0374812
TMEM106A	113277	0.523001406	-0.935113269	0.0180965
TOM1L2	146691	0.522496561	-0.936506553	0.008431
DENND3	22898	0.522490287	-0.936523876	0.0134941
FSTL3	10272	0.52223162	-0.937238284	0.0068578
SKIV2L	6499	0.52218307	-0.93737241	0.0169711
KIF21B	23046	0.522155426	-0.937448788	0.0149748
ARHGAP27	201176	0.522004982	-0.93786452	0.0307792
CABLES2	81928	0.521781092	-0.93848343	0.0029651
SH2D3C	10044	0.521665049	-0.938804318	0.0134454
PVR	5817	0.521484022	-0.939305045	0.0106006
USP35	57558	0.520980727	-0.940698093	0.0296998
RAPGEF3	10411	0.520844852	-0.941074404	0.0249758
COL1A1	1277	0.520388334	-0.942339474	0.0216943
LAMB2	3913	0.51955151	-0.944661305	0.024103
SYNGR1	9145	0.519133231	-0.945823255	0.0436698
AGAP4	119016	0.519061035	-0.946023904	0.0069425
HOOK2	29911	0.51877761	-0.94681188	0.0472599
LRRC32	2615	0.51864576	-0.947178593	0.0103972
ZNF70	7621	0.518404332	-0.94785032	0.0115659
PDGFRB	5159	0.517775389	-0.949601703	0.0327089
CABIN1	23523	0.516798345	-0.952326645	0.0115159
CARD10	29775	0.51666474	-0.952699666	0.0237724
ITGA7	3679	0.516254696	-0.953845096	0.0290011
TPCN1	53373	0.516233453	-0.95390446	0.0032836
SMPD4	55627	0.515633078	-0.95558328	0.0088948
GOLGA6L9	440295	0.514290515	-0.959344548	0.0238002
ZNF862	643641	0.513674856	-0.961072637	0.0459003
PLAT	5327	0.513662719	-0.961106726	0.0035703
PCDHGA2	56113	0.513492386	-0.96158521	0.0304932
PTPRF	5792	0.513472501	-0.96164108	0.0078774
RAB40C	57799	0.512887097	-0.963286817	0.0306074
SETD1A	9739	0.512413431	-0.964619803	0.025288
PNPLA2	57104	0.512127477	-0.96542513	0.0388472
TRERF1	55809	0.511981474	-0.965836487	0.0320213
MAP3K6	9064	0.511230058	-0.967955433	0.0293902
GPC1	2817	0.511045544	-0.968476226	0.0097408
SLC29A3	55315	0.510867433	-0.968979126	0.0252856
IGLON5	402665	0.510866697	-0.968981204	0.0089016
USP36	57602	0.510172479	-0.970943019	0.0007023
GGT5	2687	0.50952415	-0.972777567	0.0198555
DENND6B	414918	0.509252566	-0.97354675	0.0151229

© 2022. Luo D et al. Published online at http://www.aginganddisease.org/EN/10.14336/AD.2022.0607

SUPPLEMENTARY DATA

MBD6	114785	0.509216285	-0.973649537	0.0065371
SORCS2	57537	0.50893186	-0.974455587	0.0024158
SYNGAP1	8831	0.50890791	-0.97452348	0.010707
BCL9L	283149	0.508374954	-0.976035139	0.0175448
SLC7A5	8140	0.50761382	-0.978196747	0.0053064
NOP2	4839	0.50756123	-0.978346222	0.0012792
NYAP1	222950	0.506705753	-0.980779885	0.0439575
JAKMIP3	282973	0.506576721	-0.981147312	0.027066
MAP3K9	4293	0.506576001	-0.981149363	0.0351069
MAPK13	5603	0.506441721	-0.981531836	0.0047428
PALM3	342979	0.506000345	-0.982789726	0.0265988
NAV1	89796	0.505719179	-0.983591602	0.0010292
CLCN7	1186	0.505367904	-0.984594054	0.0381853
FAM20C	56975	0.50530527	-0.984772869	0.0190264
BCL3	602	0.50458838	-0.986821112	0.0161116
NADSYN1	55191	0.50377919	-0.989136566	0.0050172
PAN2	9924	0.503424509	-0.99015264	0.0156283
LYNX1	66004	0.503065625	-0.991181482	0.0343019
ITGA5	3678	0.502830122	-0.991857018	0.0470352
KIAA1549	57670	0.502793739	-0.991961411	0.0003944
N4BP3	23138	0.502017588	-0.994190186	0.0143675
SLC25A29	123096	0.501920273	-0.994469876	0.0371807
PLXNA2	5362	0.501900032	-0.994528056	0.0061765
SLC12A7	10723	0.501672581	-0.995182006	0.0034081
IGHMBP2	3508	0.501613284	-0.995352539	0.0128097
LOC652276	652276	0.501516552	-0.995630778	0.0126702
PLOD3	8985	0.500370399	-0.99893165	0.0091096
NOD1	10392	0.500368401	-0.998937412	0.0299019
CNOT3	4849	0.500243569	-0.999297379	0.0087635
MXRA5	25878	0.499586765	-1.001192838	0.0420869
CEP164	22897	0.498697332	-1.003763609	0.0082106
POMT2	29954	0.497676883	-1.006718718	0.0299436
LMF1	64788	0.497461511	-1.007343187	0.022274
ACKR3	57007	0.497376536	-1.007589644	0.0065427
LRRK1	79705	0.497012969	-1.008644596	0.0447873
RUNX3	864	0.4969763	-1.00875104	0.0233448
UBR4	23352	0.496933772	-1.008874503	0.0014051
GRIN2B	2904	0.496534669	-1.010033642	0.0263868
POLR2J4	84820	0.496125313	-1.011223527	0.0150355
RHOT2	89941	0.495042591	-1.014375442	0.0201561
MIR4697HG	283174	0.495023486	-1.014431121	0.040909
CCDC144CP	348254	0.494723006	-1.015307103	0.0334423
CDH3	1001	0.494450428	-1.016102206	0.0068699
LIMD2	80774	0.493392124	-1.019193409	0.0177074
PCDHB7	56129	0.493139064	-1.019933555	0.0155919
BRSK2	9024	0.492811569	-1.02089197	0.0204162

© 2022. Luo D et al. Published online at http://www.aginganddisease.org/EN/10.14336/AD.2022.0607

KLHL29	114818	0.492791015	-1.020952143	0.0444502
NEB	4703	0.49249351	-1.021823381	0.0082894
PAQR4	124222	0.491679913	-1.024208677	0.0040016
TAZ	6901	0.491367785	-1.025124819	0.0436317
ULK1	8408	0.491180358	-1.025675224	0.0353852
FLRT1	23769	0.490305918	-1.02824592	0.0397073
MYO7A	4647	0.489818738	-1.029680129	0.0106382
JAG2	3714	0.489583649	-1.03037272	0.0301403
TRPM4	54795	0.489538677	-1.030505248	0.0154821
LOXL2	4017	0.488852713	-1.032528235	0.0242667
QSOX1	5768	0.48826351	-1.034268131	0.0212926
IQSEC2	23096	0.487832965	-1.035540844	0.01771
AKNA	80709	0.486624676	-1.039118616	0.0147227
WASH7P	653635	0.485759209	-1.041686748	0.0080199
NBEAL2	23218	0.48535742	-1.042880548	0.0234833
PLXNB2	23654	0.485296267	-1.043062332	0.0083015
ZNF500	26048	0.485189334	-1.043380259	0.0157183
RBM14	10432	0.485155908	-1.043479654	0.0044898
RAP1GAP2	23108	0.484573646	-1.045212151	0.0122928
PCDH1	5097	0.484302491	-1.046019673	0.0066711
FAM222A	84915	0.483933492	-1.047119307	0.0144241
TJAP1	93643	0.483600244	-1.048113122	0.0031616
TPCN2	219931	0.483465375	-1.048515525	0.0170672
ALG12	79087	0.482959606	-1.050025566	0.0413059
ZNF324B	388569	0.482241709	-1.05217166	0.0030226
TMEM201	199953	0.481547473	-1.054250062	0.0043326
NOTCH3	4854	0.481319205	-1.054934105	0.0388629
ADCY1	107	0.481236843	-1.055180996	0.0370839
ENGASE	64772	0.481083408	-1.05564105	0.0136442
FAM83H	286077	0.480217699	-1.058239518	0.0422465
NAV2	89797	0.478154991	-1.064449761	0.0001616
SCN2B	6327	0.478068184	-1.064711698	0.0414262
KIAA0895L	653319	0.477988415	-1.064952442	0.0238844
CSPG4	1464	0.477910644	-1.065187196	0.0109425
MYBBP1A	10514	0.477750739	-1.06566999	0.0112643
LINC00265	349114	0.477710479	-1.06579157	0.0175593
CAPN15	6650	0.477549023	-1.066279254	0.0222334
PCDHGA4	56111	0.477526786	-1.066346435	0.0445632
KCNQ4	9132	0.47707144	-1.067722774	0.0437547
DTX2	113878	0.477035879	-1.067830315	0.0053073
NLGN2	57555	0.47699858	-1.067943125	0.0201161
STEAP3	55240	0.475869947	-1.071360748	0.0247871
UAP1L1	91373	0.475746785	-1.071734187	0.0139344
MED25	81857	0.4756278	-1.072095053	0.010379
VWA3A	146177	0.475616798	-1.072128426	0.0281632
XYLT1	64131	0.474977835	-1.074067905	0.000162

© 2022. Luo D et al. Published online at http://www.aginganddisease.org/EN/10.14336/AD.2022.0607

ANKRD52	283373	0.474641903	-1.075088622	0.0026462
BMP1	649	0.474569294	-1.075309339	0.0005193
CLN6	54982	0.473689249	-1.077987165	0.0133646
MDGA1	266727	0.473406228	-1.078849408	0.0185085
CEP170B	283638	0.472918148	-1.080337589	0.0307241
HERC2P2	400322	0.47288775	-1.080430326	0.0081519
INF2	64423	0.471703641	-1.084047359	0.0184785
RPL32P3	132241	0.471470545	-1.084760455	0.0052798
CROCC	9696	0.470760002	-1.086936346	0.0406265
VARS2	57176	0.47030513	-1.088331026	0.0156339
COLGALT1	79709	0.469644518	-1.090358926	0.0057029
ATG2A	23130	0.469563352	-1.09060828	0.0248478
SDK1	221935	0.469415893	-1.091061407	0.0078257
SLC6A12	6539	0.468812887	-1.092915866	0.0220125
TMEM161A	54929	0.468513425	-1.093837708	0.0287604
ZDHHC23	254887	0.46810896	-1.095083715	0.006397
ZNF276	92822	0.468082769	-1.095164437	0.0075836
SDHAP2	727956	0.467695321	-1.096359099	0.0022618
SEMA6C	10500	0.467202121	-1.097881272	0.0448928
AATK	9625	0.466869715	-1.098908089	0.0455922
EMC3-AS1	442075	0.466840074	-1.098999687	0.0204236
SH3RF3	344558	0.466620753	-1.099677622	0.0019583
PRIMA1	145270	0.466620551	-1.099678246	0.0235575
HID1	283987	0.466443762	-1.100224945	0.0456465
PCDHB6	56130	0.466417279	-1.10030686	0.012915
H6PD	9563	0.465898227	-1.101913256	0.0043713
MTHFR	4524	0.46586526	-1.102015342	0.0095569
POFUT2	23275	0.46538767	-1.103495107	0.0060079
PROSER3	148137	0.465330488	-1.103672381	0.0243666
SLC9A1	6548	0.463843359	-1.10829041	0.0190038
SLC16A3	9123	0.463688856	-1.10877104	0.0085177
LRP5L	91355	0.46362974	-1.108954983	0.0375189
HAUS5	23354	0.463147842	-1.110455303	0.0247165
SLC43A1	8501	0.462937999	-1.111109106	0.0071521
CNNM4	26504	0.462899357	-1.111229535	0.0015728
TPPP	11076	0.462521478	-1.112407735	0.0338465
MAML1	9794	0.462274965	-1.113176861	0.0004445
CAND2	23066	0.461958315	-1.114165421	0.0246509
PPIL2	23759	0.461343925	-1.116085437	0.00131
PAPLN	89932	0.461219607	-1.116474251	0.0162561
VWCE	220001	0.461108952	-1.116820421	0.0077625
ZFYVE28	57732	0.461082747	-1.116902412	0.0229868
SEMA3F	6405	0.460698411	-1.118105472	0.0066315
CPAMD8	27151	0.460199536	-1.119668567	0.019995
TRPV1	7442	0.459452294	-1.122013023	0.0170313
DMWD	1762	0.458913803	-1.123704895	0.0033843

© 2022. Luo D et al. Published online at http://www.aginganddisease.org/EN/10.14336/AD.2022.0607

DCHS1	8642	0.4586969	-1.124386937	0.0119748
ABCA3	21	0.458317204	-1.125581654	0.0206487
SPAG5	10615	0.456528516	-1.131223117	0.0070042
ARHGEF1	9138	0.455989476	-1.132927568	0.0271535
PRAM1	84106	0.455359526	-1.134922031	0.0404066
PHLDB1	23187	0.454750789	-1.136851953	0.0076636
LRRC14	9684	0.454256585	-1.138420668	0.0055338
FCHSD1	89848	0.453229646	-1.141685863	0.0165828
TRRAP	8295	0.453160427	-1.141906214	0.0003291
RAPGEFL1	51195	0.453027589	-1.142329184	0.0094402
SNED1	25992	0.451730778	-1.146464881	0.0184909
GRIN2C	2905	0.451029527	-1.14870621	0.0014341
PDZD7	79955	0.450544463	-1.150258606	0.0458678
TTBK1	84630	0.449688444	-1.153002285	0.0289826
ITGA3	3675	0.449537311	-1.153487233	0.0040534
LOC101927550	101927550	0.449118875	-1.154830739	0.0448227
BCORL1	63035	0.448582805	-1.156553773	0.0115138
CPSF1	29894	0.448362489	-1.15726251	0.0135027
MROH6	642475	0.447708066	-1.159369786	0.0462108
ERBB3	2065	0.447503716	-1.160028432	0.0001624
TRIM62	55223	0.447214554	-1.160960956	0.0039995
MAN2A2	4122	0.44637001	-1.163687994	0.0269919
PITPNM2	57605	0.446367781	-1.163695197	0.0032947
KCNQ2	3785	0.445735381	-1.165740613	0.0243287
CCDC57	284001	0.44569983	-1.165855685	0.0092207
RHOF	54509	0.445480024	-1.166567355	0.0225962
HR	55806	0.443758097	-1.17215465	0.0299896
SLC12A9	56996	0.443512791	-1.172952382	0.026485
PLEKHH3	79990	0.443473859	-1.17307903	0.0338892
SOGA1	140710	0.443247309	-1.173816221	0.0049534
RHBDF2	79651	0.443160977	-1.174097246	0.0486928
STAG3L2	442582	0.44288475	-1.174996772	0.0106559
CCDC120	90060	0.441511534	-1.179476968	0.0017237
POU2F2	5452	0.441494373	-1.179533043	0.0393476
MROH1	727957	0.441386611	-1.179885229	0.029191
AP5Z1	9907	0.441039186	-1.181021252	0.0041587
ZNF646	9726	0.440740046	-1.182000106	0.0116404
NOS3	4846	0.440643072	-1.182317574	0.0038515
OSBPL7	114881	0.440549396	-1.182624307	0.0451469
SLC6A6	6533	0.440431619	-1.183010048	0.0044029
TMC6	11322	0.43917343	-1.187137321	0.0153243
VASN	114990	0.4390699	-1.187477459	0.0124746
EPHA2	1969	0.438488952	-1.189387603	0.0370295
KIFC2	90990	0.437170182	-1.193733093	0.0253294
SLC5A6	8884	0.436588235	-1.19565484	0.0018522
FCHO1	23149	0.436042276	-1.197460079	0.018221

© 2022. Luo D et al. Published online at http://www.aginganddisease.org/EN/10.14336/AD.2022.0607

SUPPLEMENTARY DATA

DLL1	28514	0.435893785	-1.197951462	0.0146423
FAM160A2	84067	0.43529577	-1.199932095	0.0076308
MYO19	80179	0.435108627	-1.200552472	0.0054049
CLUHP3	100132341	0.434639897	-1.202107484	0.0379914
CACNA1H	8912	0.4342454	-1.203417528	0.0302261
PCDH15	65217	0.434175782	-1.203648841	7.73E-05
CCDC142	84865	0.434016531	-1.204178102	0.0106173
NDST2	8509	0.432994431	-1.207579625	0.0024302
GUSBP11	91316	0.43262332	-1.208816661	0.0234115
ATP2A3	489	0.432168559	-1.210333976	0.0261051
CAD	790	0.431884206	-1.211283538	0.0033821
LHFPL4	375323	0.430607525	-1.215554563	0.0167716
LDLR	3949	0.430419199	-1.216185664	0.007955
C1orf159	54991	0.430199383	-1.216922639	0.0254881
RTN4RL2	349667	0.429925717	-1.217840683	0.0495288
GUCA1B	2979	0.429397766	-1.21961341	0.0245791
MTUS2	23281	0.429393775	-1.219626819	0.0037874
CCDC88C	440193	0.428793594	-1.221644742	0.010601
SLC38A10	124565	0.428449257	-1.222803744	0.0092821
TTYH2	94015	0.428437168	-1.222844452	0.0222464
NLRP1	22861	0.427680124	-1.225395933	0.044508
ZFP36	7538	0.427668187	-1.2254362	0.0207176
ZNF512B	57473	0.427321925	-1.226604755	0.0079496
LTBP3	4054	0.427302228	-1.226671256	0.0260922
DGKQ	1609	0.426426847	-1.229629824	0.046536
PKD1L1	168507	0.426086959	-1.230780198	0.0277373
CTU1	90353	0.425919531	-1.231347206	0.0332176
ANKRD24	170961	0.425715381	-1.232038881	0.0266054
EP400	57634	0.425415132	-1.233056744	0.0002837
ANKRD13B	124930	0.425168793	-1.233892385	0.0246826
IL4R	3566	0.42472951	-1.235383745	0.000507
SLCO4A1	28231	0.424536213	-1.236040472	0.0040285
PCDHB8	56128	0.424343859	-1.236694295	0.0162576
PLEKHG2	64857	0.423806535	-1.238522262	0.0051751
CSAD	51380	0.423780089	-1.23861229	0.0248925
P2RX7	5027	0.423382349	-1.239966969	0.0038315
KCNH3	23416	0.423010512	-1.241234581	0.0380072
DMPK	1760	0.4224971	-1.242986657	0.0038714
PKN3	29941	0.421752434	-1.245531702	0.0046023
ANKRD23	200539	0.421438837	-1.246604824	0.0389907
SLC29A4	222962	0.421417243	-1.246678748	0.0017794
PTPN23	25930	0.421035797	-1.247985198	0.0088501
CA4	762	0.42101983	-1.248039909	0.018617
ABCB8	11194	0.420490255	-1.249855727	0.013477
CLASRP	11129	0.420480482	-1.249889261	0.0035147
PIGQ	9091	0.420477476	-1.249899576	0.0103209

© 2022. Luo D et al. Published online at http://www.aginganddisease.org/EN/10.14336/AD.2022.0607

CCNL2	81669	0.419204674	-1.254273293	0.001851
ADGRA2	25960	0.419114472	-1.254583755	0.0034542
PER1	5187	0.41895026	-1.255149126	0.0091656
LRRC56	115399	0.418405077	-1.257027736	0.0456617
INTS1	26173	0.417142464	-1.261387912	0.0236193
ZDHHC8	29801	0.417103246	-1.261523556	0.0056174
GMIP	51291	0.41704341	-1.261730532	0.0033407
TRAF1	7185	0.417008741	-1.261850469	0.0003299
NCOR2	9612	0.41660891	-1.263234402	0.0046851
HSPG2	3339	0.41648474	-1.263664459	0.0074578
KSR1	8844	0.41591845	-1.265627412	0.0060164
C21orf58	54058	0.41471468	-1.26980898	0.0156098
TNXB	7148	0.414614535	-1.270157403	0.0175217
TMEM120B	144404	0.414120763	-1.271876558	0.0033164
PLCB2	5330	0.413517222	-1.27398068	0.0007025
CNTN2	6900	0.413228202	-1.274989375	0.0316783
PEAR1	375033	0.412637594	-1.27705283	0.0153564
EPCAM	4072	0.412230472	-1.278476944	0.0287266
PLXNB1	5364	0.411191975	-1.282115986	0.0177196
LINC00115	79854	0.411132	-1.282326428	0.047454
SLX4	84464	0.410377317	-1.284977106	0.0031239
MRC2	9902	0.410306011	-1.285227805	0.0034434
SHPK	23729	0.410224619	-1.285514019	0.0148557
ARID3A	1820	0.410023833	-1.286220324	0.030553
KIF26A	26153	0.409878784	-1.286730781	0.0028637
MORN1	79906	0.409741122	-1.287215405	0.0054294
FLJ42627	645644	0.409139887	-1.289333902	0.0015769
PCDHB12	56124	0.408903963	-1.290166051	0.0170535
MAG	4099	0.408650343	-1.291061148	0.0190863
ANKMY1	51281	0.407501492	-1.295122754	0.0021545
SMTN	6525	0.407263276	-1.295966366	0.0003854
COL11A2	1302	0.407138327	-1.296409054	0.0437961
ADGRA1	84435	0.406871395	-1.297355238	0.031142
GREM1	26585	0.406555719	-1.298475005	0.0186138
ARHGAP39	80728	0.406276734	-1.299465347	0.0213879
KRTAP5-AS1	338651	0.405171845	-1.303394167	0.0103386
PDE4C	5143	0.403996806	-1.307584206	0.0434796
PCDHGB5	56101	0.403049731	-1.310970235	0.0033722
COL5A1	1289	0.401951723	-1.314905862	0.0300485
GALNT6	11226	0.401631164	-1.316056876	0.0125543
TET3	200424	0.400441141	-1.320337894	0.0046472
PRKXP1	441733	0.400298138	-1.320853188	0.0218592
HDAC7	51564	0.400228191	-1.321105305	0.0019505
PGAP3	93210	0.399864999	-1.322415092	0.0031014
DAGLA	747	0.399422196	-1.324013587	0.0075157
TYK2	7297	0.399135053	-1.325051111	0.0023147

© 2022. Luo D et al. Published online at http://www.aginganddisease.org/EN/10.14336/AD.2022.0607

SUPPLEMENTARY DATA

OPLAH	26873	0.398510854	-1.327309075	0.0386727
SPON2	10417	0.39818109	-1.328503386	0.0015463
TINCR	257000	0.39728603	-1.33175003	0.0461767
KIAA1755	85449	0.3970986	-1.332430821	0.0065368
ADCY4	196883	0.397061007	-1.332567406	0.001385
NEAT1	283131	0.397024755	-1.33269913	0.0135998
MICAL1	64780	0.396622295	-1.334162319	0.0372614
RDH13	112724	0.396094576	-1.336083149	0.0262611
STK36	27148	0.395345399	-1.338814461	0.0189532
HIF3A	64344	0.395196241	-1.339358871	0.0002203
AFG3L1P	172	0.394356453	-1.342427845	0.0011349
LOC283922	283922	0.3942376	-1.342862718	0.0053922
LOC150776	150776	0.394058346	-1.343518839	0.0024511
WDFY4	57705	0.393968951	-1.343846159	0.0107499
PPFIA4	8497	0.393599611	-1.345199301	0.034661
PLIN4	729359	0.392610637	-1.348828834	0.0065838
ARAP3	64411	0.392220916	-1.350261622	0.0112845
DOCK6	57572	0.392210224	-1.350300952	0.0018018
CFAP46	54777	0.391750557	-1.351992769	0.0295902
TBC1D2	55357	0.391736379	-1.352044983	0.0033394
ADAM19	8728	0.391628752	-1.352441408	0.00019
BSN	8927	0.39141986	-1.353211138	0.0212313
PCDHB9	56127	0.391061331	-1.354533208	0.0126719
MYH3	4621	0.38894654	-1.362356222	0.0392038
CARNS1	57571	0.387430027	-1.367992325	0.0419215
FAAP100	80233	0.386825002	-1.370247049	0.0094537
PHF21B	112885	0.386741314	-1.370559206	0.0002624
HELZ2	85441	0.385963764	-1.373462686	0.001487
NOTCH1	4851	0.385813829	-1.374023239	0.0016089
SETD1B	23067	0.385734755	-1.374318957	0.0069883
SYVN1	84447	0.385562516	-1.374963295	0.0074446
POLE	5426	0.385270373	-1.376056849	0.0182128
LLGL2	3993	0.384617739	-1.378502792	0.0097541
SHANK1	50944	0.384117457	-1.380380563	0.01611
TUBGCP6	85378	0.383593256	-1.38235074	0.0093451
NDOR1	27158	0.383225528	-1.383734428	0.0254676
TIGD5	84948	0.382534193	-1.386339386	0.0035244
KMT2E-AS1	100216545	0.382358341	-1.387002749	0.0072107
ZNF865	100507290	0.381688092	-1.389533919	0.0079584
AP5B1	91056	0.381434578	-1.390492461	0.0018224
MEGF6	1953	0.381254613	-1.391173303	0.0005729
FAM160B2	64760	0.381155489	-1.391548442	0.0028761
GPR179	440435	0.380809957	-1.392856892	0.0248825
PCDHGA9	56107	0.380696077	-1.393288391	0.0044872
NOTCH4	4855	0.380288158	-1.39483508	0.0010752
MLXIP	22877	0.379964737	-1.396062562	0.0025593

© 2022. Luo D et al. Published online at http://www.aginganddisease.org/EN/10.14336/AD.2022.0607

SLC7A5P2	387254	0.379903467	-1.396295215	0.0188549
JAK3	3718	0.379762023	-1.396832453	0.0030407
KDM6B	23135	0.379238458	-1.398822823	0.0012999
SLC52A3	113278	0.379166305	-1.399097331	0.0066348
GPRC5A	9052	0.378375005	-1.402111307	0.0325071
TMEM235	283999	0.378090438	-1.403196729	0.0355823
MAST1	22983	0.376876376	-1.407836732	0.0184317
SYT7	9066	0.376715175	-1.408453945	0.0152826
NACAD	23148	0.376693568	-1.408536694	0.0126228
STAB1	23166	0.375796133	-1.411977874	0.0276325
PLEKHH1	57475	0.374676951	-1.416280863	0.0083573
TNRC6C-AS1	100131096	0.374282011	-1.417802386	0.0444678
PRR12	57479	0.374106184	-1.418480282	0.0155793
ADGRB1	575	0.373802289	-1.419652693	0.0195874
ATP13A1	57130	0.37278489	-1.423584709	0.0120179
TMEM175	84286	0.371243211	-1.429563452	0.0079595
ARHGAP33	115703	0.370867547	-1.431024067	0.0113038
LOC100130744	100130744	0.370435038	-1.432707531	0.0008717
SDHAP1	255812	0.370182198	-1.433692576	0.0001094
TMEM104	54868	0.369756347	-1.435353184	0.0004858
TRIM46	80128	0.369334615	-1.436999614	0.0473742
GRIK3	2899	0.369319671	-1.437057988	0.0089521
SEC14L5	9717	0.369298172	-1.437141974	0.0099046
PCDHGA12	26025	0.367977089	-1.442312151	0.0001752
KMT2B	9757	0.367907858	-1.442583605	0.005017
CHST3	9469	0.367527206	-1.444077048	2.27E-05
CNGB1	1258	0.367526339	-1.44408045	0.0231626
PRRG3	79057	0.367505045	-1.44416404	0.0394046
SPTBN5	51332	0.3672932	-1.444995909	0.0423879
LOC155060	155060	0.367088721	-1.445799306	0.0402205
TCIRG1	10312	0.366591351	-1.447755347	0.0104788
WDR81	124997	0.366107053	-1.449662526	0.0056766
PCSK7	9159	0.36577116	-1.450986767	0.0002993
RAD9A	5883	0.364750667	-1.45501748	0.011756
ZNF783	100289678	0.364042206	-1.457822374	0.0057707
SREBF1	6720	0.363983695	-1.458054272	0.0031371
CASZ1	54897	0.363923469	-1.458293002	0.0001737
CCNF	899	0.363835869	-1.458640316	0.0004964
CHD5	26038	0.36371644	-1.459113958	0.0274709
DNHD1	144132	0.36364778	-1.459386326	0.0050232
MUC12	10071	0.362345275	-1.464563013	0.0470271
SH3D21	79729	0.361390752	-1.468368507	0.0311742
CROCCP3	114819	0.360795339	-1.470747395	0.0242945
KCNC3	3748	0.359548336	-1.475742362	0.0317843
LPIN3	64900	0.357954961	-1.48215002	0.0007899
ADAMTS10	81794	0.357796132	-1.482790304	0.0022328

© 2022. Luo D et al. Published online at http://www.aginganddisease.org/EN/10.14336/AD.2022.0607

ACOT11	26027	0.357354755	-1.48457111	0.0010565
ATAD3B	83858	0.356348602	-1.488638831	0.0057631
HSF4	3299	0.355837672	-1.49070884	0.0142054
ANO8	57719	0.355083609	-1.49376933	0.0332137
GGT1	2678	0.352951763	-1.502457066	0.0067694
RHBDF1	64285	0.351760547	-1.507334412	0.0003533
LOC727751	727751	0.351735968	-1.507435225	0.0463962
NYNRIN	57523	0.351549053	-1.508202087	0.0041294
TNRC18	84629	0.35147346	-1.508512338	0.0097617
PLXNA4	91584	0.351419501	-1.508733844	0.0119374
ADAMTS13	11093	0.3513775	-1.508906282	0.0146772
ANKRD18A	253650	0.351234514	-1.509493477	0.0006748
LRFN3	79414	0.350557927	-1.512275237	0.0053753
SPEG	10290	0.349336362	-1.517311275	0.0110484
THBS1	7057	0.349224124	-1.517774874	0.0048429
LINC00639	283547	0.349105066	-1.518266804	0.0072766
AHDC1	27245	0.348167108	-1.52214818	0.0037031
PLXNA3	55558	0.347601529	-1.524493665	0.0159591
LOC440300	440300	0.345872274	-1.531688728	0.0283088
UNC5A	90249	0.345806468	-1.531963243	0.0353702
BRICD5	283870	0.34539047	-1.533699819	0.0490166
SRCAP	10847	0.344946624	-1.535554954	0.000501
LOC101928323	101928323	0.344579783	-1.537090035	0.0094514
RRP7BP	91695	0.344498186	-1.53743171	0.0118131
PPRC1	23082	0.343438916	-1.541874572	0.0005124
LINC00965	349196	0.3431854	-1.542939916	0.0028155
UBAP1L	390595	0.343023069	-1.543622493	0.0215841
MAU2	23383	0.342883779	-1.54420844	0.0004498
CELSR2	1952	0.342850337	-1.544349153	0.0358941
SLC30A3	7781	0.342432339	-1.546109142	0.0226661
TMEM86B	255043	0.342276216	-1.54676705	0.016952
MICAL3	57553	0.342014969	-1.547868627	0.0032555
LPCAT4	254531	0.339167921	-1.559928372	0.0076877
ZGPAT	84619	0.339109326	-1.560177634	0.0086743
HIC2	23119	0.338361223	-1.563363855	0.0032985
ZNF324	25799	0.338336033	-1.563471262	0.0013902
DGCR9	25787	0.337109814	-1.568709469	0.0496712
GRID2IP	392862	0.337067735	-1.568889558	0.0332457
ZSWIM8	23053	0.336950381	-1.569391939	0.0014734
PCDHGB3	56102	0.336534843	-1.571172212	0.0179297
PXDN	7837	0.336200277	-1.572607182	0.0010925
CACNA1B	774	0.336049483	-1.573254412	0.0121845
ICOSLG	23308	0.335831612	-1.574190059	0.0075842
ZNF341	84905	0.335683382	-1.574826977	0.0007968
ZFP41	286128	0.335520434	-1.575527461	0.001527
COL16A1	1307	0.334949854	-1.577982973	0.0072368

© 2022. Luo D et al. Published online at http://www.aginganddisease.org/EN/10.14336/AD.2022.0607

HIVEP3	59269	0.33269277	-1.587737582	0.0006224
PLXNB3	5365	0.332614018	-1.588079121	0.0200229
PRR36	80164	0.331861521	-1.591346736	0.0173603
CACNA1A	773	0.329590867	-1.601251828	0.0127063
SLC19A1	6573	0.329195571	-1.60298317	0.0133641
RAI1	10743	0.326873559	-1.613195412	0.0065914
GBAP1	2630	0.326749543	-1.613742877	0.0360624
PKD1P6	353511	0.326737351	-1.613796709	0.0012808
LRCH4	4034	0.325585216	-1.618892903	0.006494
PCDHGA6	56109	0.325494085	-1.61929677	0.0394449
NSUN5P1	155400	0.32522901	-1.620472145	0.0037434
CPLX3	594855	0.323969305	-1.626070968	0.0141455
DBF4B	80174	0.323910014	-1.626335023	0.0002943
COL7A1	1294	0.323183249	-1.629575671	0.0154474
TAF1C	9013	0.322774609	-1.631401002	0.0231148
ITPR3	3710	0.322222378	-1.633871406	0.0003241
PCDHGC4	56098	0.32039618	-1.642071149	0.0222172
RGS11	8786	0.319763171	-1.644924309	0.0062623
EPHA10	284656	0.319754329	-1.644964203	0.0136419
SLC35E4	339665	0.318912547	-1.648767237	0.0410468
NR4A1	3164	0.317797995	-1.653818074	0.0084459
OBSCN	84033	0.317124605	-1.656878277	0.0032345
SGSH	6448	0.316031611	-1.661859223	0.0080508
CES4A	283848	0.315324127	-1.665092532	0.0122028
AGRN	375790	0.315097735	-1.666128711	0.0056835
TTYH3	80727	0.314285985	-1.669850155	0.0036303
EXD3	54932	0.313755341	-1.672288079	0.0087868
MYRF	745	0.312948586	-1.676002439	0.0113187
DSG2	1829	0.312889532	-1.676274704	0.0265157
SRRM2	23524	0.31191518	-1.680774331	0.0011797
SLC5A11	115584	0.310581385	-1.686956734	0.0488201
KCNC1	3746	0.309984667	-1.689731241	0.0158301
HRH2	3274	0.309973826	-1.689781693	0.0043486
SNAPC4	6621	0.309521511	-1.691888419	0.0047559
PLXNA1	5361	0.308183815	-1.698136998	0.0026252
UNC13D	201294	0.306766651	-1.704786441	0.0047812
TMEM63C	57156	0.306274849	-1.707101196	0.0443251
SBNO2	22904	0.30595261	-1.708619889	9.18E-05
GOLGA8A	23015	0.305914356	-1.708800281	0.0012987
ARHGEF16	27237	0.30561781	-1.710199475	0.0319554
WNK2	65268	0.305407991	-1.711190285	0.0116233
FGF17	8822	0.304996072	-1.713137432	0.0302016
ALS2CL	259173	0.304551333	-1.715242675	0.0176922
CACNA1G	8913	0.304377226	-1.716067679	0.0109667
FER1L4	80307	0.303948837	-1.718099594	0.0015118
KIRREL3	84623	0.302601544	-1.724508744	0.0368581

© 2022. Luo D et al. Published online at http://www.aginganddisease.org/EN/10.14336/AD.2022.0607

KAT2A	2648	0.301957796	-1.727581176	0.0051162
RGL3	57139	0.301549152	-1.729534918	0.0092528
ZNF692	55657	0.301528494	-1.729633755	0.0029542
TRIM7	81786	0.30117424	-1.731329715	0.0086157
APC2	10297	0.297129263	-1.750837399	0.0124994
BAHCC1	57597	0.296999749	-1.751466383	0.0081056
LOC100289473	100289473	0.296690651	-1.752968626	0.0006636
MICALL2	79778	0.295912905	-1.75675548	0.0053277
ZNF469	84627	0.295196102	-1.760254424	0.0033045
SZT2	23334	0.294499168	-1.763664538	0.0055972
AP1G2	8906	0.293543735	-1.768352629	0.0041963
TRANK1	9881	0.293398866	-1.769064801	0.0004436
SHISA7	729956	0.291734499	-1.777272093	0.0044989
ZDHHC11	79844	0.290688755	-1.78245283	0.0040828
STAG3L5P-PVRIG2P-PILRB	101752399	0.290101391	-1.785370881	0.0015111
GOLGA6L5P	374650	0.289955762	-1.786095288	0.0293501
LOC644285	644285	0.289234965	-1.789686126	0.0162959
SLC29A2	3177	0.289023286	-1.790742365	0.0012932
NPIPB3	23117	0.28875626	-1.792075871	0.002366
GLUD1P3	2749	0.288697431	-1.792369827	0.0070073
ELFN2	114794	0.287243878	-1.799651951	0.0049368
PKD1P1	339044	0.285820998	-1.806816186	0.0089664
TRPM2	7226	0.284076572	-1.815648239	0.0101622
GOLGA6L4	643707	0.284005001	-1.816011761	0.0006055
PIGR	5284	0.283230903	-1.81994941	0.0278644
COL27A1	85301	0.281997984	-1.826243246	0.0042243
CELSR3	1951	0.281807038	-1.827220454	0.0099205
CLDN15	24146	0.280549776	-1.833671333	0.0105404
ABCC10	89845	0.280469038	-1.834086578	0.0040017
MUC2	4583	0.279306194	-1.84008053	0.0461743
SLC26A6	65010	0.27844288	-1.844546691	0.0022564
ABCA7	10347	0.278058881	-1.846537678	0.0269402
FAM95C	100289137	0.277963471	-1.847032793	0.0094124
PNPLA7	375775	0.277577626	-1.849036812	0.0152689
NEU4	129807	0.276611435	-1.854067297	0.0127068
TTN	7273	0.276504332	-1.854626011	0.0008443
SH3BP5-AS1	100505696	0.276502109	-1.854637612	3.98E-05
SRRM3	222183	0.274043908	-1.86752103	0.0155433
PIEZO1	9780	0.27360526	-1.869832131	0.000506
C15orf39	56905	0.272634105	-1.874962048	0.0005861
SEC31B	25956	0.271571361	-1.88059675	0.0111281
ABCA2	20	0.27103442	-1.883452017	0.0117105
NFASC	23114	0.270993298	-1.883670922	0.0028753
KCNT1	57582	0.268357817	-1.897770184	0.012676
NUP210	23225	0.266396121	-1.90835502	1.19E-05
ADAMTS4	9507	0.266261253	-1.909085596	0.0023938

RYR1	6261	0.265345119	-1.914058085	0.0041843
CACNA1I	8911	0.264851129	-1.916746435	0.01951
LINC00342	150759	0.264569646	-1.918280544	0.0013441
CPT1B	1375	0.262659364	-1.928735076	0.0320233
MUC20	200958	0.260526121	-1.940500069	0.0017777
NLRC5	84166	0.259903687	-1.943950997	2.24E-06
ONECUT2	9480	0.257581993	-1.956896354	0.0050291
LINC00599	157627	0.256737011	-1.961636806	0.0135605
COL9A1	1297	0.256361619	-1.963747811	0.0044051
CERCAM	51148	0.256207557	-1.964615066	0.0063895
SLC45A3	85414	0.255655005	-1.967729827	0.0014949
DSCAML1	57453	0.254945317	-1.971740258	0.0021105
PI4KAP1	728233	0.254570312	-1.973863911	0.0088342
CECR7	100130418	0.254353564	-1.975092786	0.0001946
L3MBTL1	26013	0.254009805	-1.977043907	0.0058416
LOC101926935	101926935	0.253010332	-1.982731793	0.001791
LCNL1	401562	0.252678549	-1.984624902	0.0367994
KMT2D	8085	0.252187338	-1.987432251	0.0012521
CSMD2	114784	0.250832801	-1.995202074	0.001322
MLXIPL	51085	0.245931636	-2.023670765	0.0215372
ASMTL-AS1	80161	0.24584009	-2.024207893	0.0412239
MYO15A	51168	0.24336149	-2.038827206	0.0343084
LOC729218	729218	0.239786437	-2.06017804	3.63E-05
NCKAP5L	57701	0.239341624	-2.062856776	0.0036862
EPPK1	83481	0.238871521	-2.065693235	0.0003543
SLC25A25-AS1	100289019	0.237854957	-2.071846004	0.0098717
MAMDC4	158056	0.237004464	-2.077013861	0.0304782
PLCH2	9651	0.236239298	-2.081679123	0.0041595
MYO15B	80022	0.23525692	-2.087690936	0.0036374
SMIM5	643008	0.234292552	-2.093617	0.0037835
ASIC3	9311	0.233987679	-2.095495529	0.0415711
LAMA5	3911	0.233308182	-2.099691191	0.0009989
DOT1L	84444	0.231936331	-2.108199272	9.86E-05
ABCC8	6833	0.231068269	-2.113608937	0.0033887
SPPL2B	56928	0.229989893	-2.120357631	0.0042337
LOC729737	729737	0.228926925	-2.127040942	0.0053826
ZFHX2	85446	0.228697961	-2.128484593	0.0106801
PDIA2	64714	0.227625551	-2.135265586	0.0135179
TMEM63A	9725	0.226866455	-2.140084793	0.0013966
DNAH1	25981	0.218599848	-2.1936357	0.0002882
MEG3	55384	0.217510356	-2.200844003	0.0055344
LOC105447645	105447645	0.21697637	-2.204390161	0.0154811
SSPO	23145	0.215639297	-2.213307984	0.0175812
STARD9	57519	0.215038873	-2.217330611	6.62E-05
PIDD1	55367	0.21055247	-2.247748297	0.0052231
IGSF9B	22997	0.208292301	-2.263318578	0.0036572

© 2022. Luo D et al. Published online at http://www.aginganddisease.org/EN/10.14336/AD.2022.0607

ITIH4	3700	0.206099059	-2.278590174	0.0015893
PKD1	5310	0.202940328	-2.300872511	0.0097394
MIRLET7BHG	400931	0.199349985	-2.326624598	0.0132324
TOR4A	54863	0.190897262	-2.389131685	0.0088515
COL20A1	57642	0.186924659	-2.41947119	0.008258
LENG8	114823	0.185794825	-2.42821778	0.0008805
PTCH2	8643	0.179532237	-2.477685179	0.0024347
GLI2	2736	0.179526543	-2.477730931	0.0008728
IGFN1	91156	0.173554087	-2.526542754	0.0015194
HSFX1	100506164	0.169370701	-2.561743767	0.0231353
STRC	161497	0.16827641	-2.571095153	0.0031845
MIAT	440823	0.160653102	-2.637979258	0.0012369
GOLGA6L3	100133220	0.16037359	-2.640491516	0.0181778
KCNQ1OT1	10984	0.158077985	-2.661291636	0.0003249
CD22	933	0.151370853	-2.723840658	0.0015955
LINC00174	285908	0.148706181	-2.749463479	0.0002144
ADAM33	80332	0.119449848	-3.065523081	0.0001777

Supplementary Table 4. Key GO terms enrichment of differentially expressed mRNAs.

ONTOLOGY	Description	*P*-value	*P*.adjust	qvalue	Count
BP	axon guidance	3.90E-11	5.59E-08	5.15E-08	42
BP	neuron projection guidance	4.37E-11	5.59E-08	5.15E-08	42
BP	semaphorin-plexin signaling pathway involved in axon guidance	2.07E-10	2.11E-07	1.95E-07	9
BP	axonogenesis	7.79E-09	4.97E-06	4.59E-06	53
BP	synapse organization	3.11E-08	1.76E-05	1.63E-05	48
BP	homophilic cell adhesion via plasma membrane adhesion molecules	1.23E-14	6.30E-11	5.80E-11	36
BP	cell-cell adhesion via plasma-membrane adhesion molecules	2.70E-13	6.90E-10	6.36E-10	45
BP	semaphorin-plexin signaling pathway involved in neuron projection guidance	6.44E-10	5.48E-07	5.06E-07	9
BP	semaphorin-plexin signaling pathway	1.70E-09	1.24E-06	1.14E-06	14
BP	extracellular matrix organization	3.74E-08	1.87E-05	1.73E-05	45
BP	extracellular structure organization	4.03E-08	1.87E-05	1.73E-05	45
BP	synapse assembly	1.76E-06	7.49E-04	6.91E-04	25
BP	collagen fibril organization	7.24E-06	2.84E-03	2.62E-03	12
BP	cell junction assembly	7.78E-06	2.84E-03	2.62E-03	42
BP	regulation of insulin secretion	1.39E-05	4.65E-03	4.29E-03	23
BP	negative regulation of cell adhesion	1.46E-05	4.65E-03	4.29E-03	32
BP	regulation of axonogenesis	2.17E-05	6.32E-03	5.83E-03	23
BP	ossification involved in bone maturation	2.23E-05	6.32E-03	5.83E-03	7
BP	negative regulation of cell projection organization	2.58E-05	6.94E-03	6.40E-03	23
BP	regulation of synaptic plasticity	3.33E-05	8.50E-03	7.83E-03	23
BP	negative regulation of neuron projection development	3.62E-05	8.81E-03	8.13E-03	20
BP	cAMP metabolic process	4.51E-05	1.04E-02	9.57E-03	7
BP	cAMP biosynthetic process	4.67E-05	1.04E-02	9.57E-03	5

© 2022. Luo D et al. Published online at http://www.aginganddisease.org/EN/10.14336/AD.2022.0607

BP	protein localization to extracellular region	5.15E-05	1.10E-02	1.01E-02	42
BP	regulation of protein secretion	5.81E-05	1.17E-02	1.08E-02	34
BP	negative regulation of neuron differentiation	5.98E-05	1.17E-02	1.08E-02	25
BP	bone maturation	6.22E-05	1.17E-02	1.08E-02	7
BP	regulation of cell morphogenesis involved in differentiation	6.40E-05	1.17E-02	1.08E-02	31
BP	insulin secretion	6.68E-05	1.18E-02	1.08E-02	24
BP	protein secretion	7.36E-05	1.25E-02	1.16E-02	41
BP	establishment of protein localization to extracellular region	7.72E-05	1.27E-02	1.17E-02	41
BP	organic acid transmembrane transport	8.68E-05	1.36E-02	1.26E-02	19
BP	regulation of axon extension involved in axon guidance	8.81E-05	1.36E-02	1.26E-02	8
BP	bone development	9.31E-05	1.40E-02	1.29E-02	23
BP	actin filament-based movement	1.04E-04	1.51E-02	1.40E-02	19
BP	negative regulation of smoothened signaling pathway	1.12E-04	1.58E-02	1.46E-02	8
BP	positive regulation of transcription of Notch receptor target	1.20E-04	1.66E-02	1.53E-02	6
BP	branched-chain amino acid transport	1.36E-04	1.82E-02	1.68E-02	5
BP	negative regulation of axon extension	1.42E-04	1.86E-02	1.72E-02	9
BP	import across plasma membrane	1.47E-04	1.87E-02	1.73E-02	19
BP	regulation of axon guidance	1.72E-04	2.09E-02	1.93E-02	9
BP	memory	1.72E-04	2.09E-02	1.93E-02	16
BP	regulation of peptide hormone secretion	1.78E-04	2.12E-02	1.95E-02	23
BP	animal organ maturation	1.91E-04	2.22E-02	2.05E-02	7
BP	regulation of epithelial to mesenchymal transition	2.12E-04	2.35E-02	2.17E-02	14
BP	bone trabecula morphogenesis	2.12E-04	2.35E-02	2.17E-02	5
BP	response to muramyl dipeptide	2.32E-04	2.52E-02	2.32E-02	6
BP	carboxylic acid transmembrane transport	2.41E-04	2.54E-02	2.34E-02	18
BP	endodermal cell differentiation	2.45E-04	2.54E-02	2.34E-02	9
BP	mesenchyme development	2.56E-04	2.54E-02	2.34E-02	28
BP	axon extension involved in axon guidance	2.63E-04	2.54E-02	2.34E-02	8
BP	neuron projection extension involved in neuron projection guidance	2.63E-04	2.54E-02	2.34E-02	8
BP	regulation of peptide secretion	2.67E-04	2.54E-02	2.34E-02	34
BP	ossification	2.68E-04	2.54E-02	2.34E-02	36
BP	positive regulation of Notch signaling pathway	2.87E-04	2.67E-02	2.46E-02	10
BP	epithelial cell development	3.06E-04	2.79E-02	2.57E-02	23
BP	left/right axis specification	3.17E-04	2.84E-02	2.62E-02	5
BP	Notch signaling pathway	3.47E-04	3.06E-02	2.82E-02	21
BP	regulation of cell morphogenesis	3.79E-04	3.27E-02	3.01E-02	41
BP	neuron projection regeneration	3.84E-04	3.27E-02	3.01E-02	10
BP	negative regulation of axonogenesis	3.93E-04	3.27E-02	3.01E-02	11
BP	nephron development	3.97E-04	3.27E-02	3.01E-02	17
BP	cell-substrate adhesion	4.07E-04	3.30E-02	3.04E-02	32
BP	modulation of chemical synaptic transmission	4.29E-04	3.42E-02	3.15E-02	38
BP	regulation of trans-synaptic signaling	4.47E-04	3.51E-02	3.24E-02	38

© 2022. Luo D et al. Published online at http://www.aginganddisease.org/EN/10.14336/AD.2022.0607

BP	negative regulation of nervous system development	5.44E-04	4.21E-02	3.88E-02	29
BP	connective tissue development	6.36E-04	4.80E-02	4.43E-02	25
BP	dendrite self-avoidance	6.39E-04	4.80E-02	4.43E-02	5
BP	regulation of smoothened signaling pathway	6.65E-04	4.90E-02	4.52E-02	12
BP	regulation of GTPase activity	6.72E-04	4.90E-02	4.52E-02	39
CC	neuron to neuron synapse	1.12E-05	8.04E-04	6.94E-04	37
CC	postsynaptic density	5.54E-05	2.46E-03	2.12E-03	33
CC	cation channel complex	1.17E-04	3.36E-03	2.90E-03	24
CC	asymmetric synapse	7.82E-05	2.86E-03	2.46E-03	33
CC	semaphorin receptor complex	3.30E-09	1.90E-06	1.64E-06	8
CC	collagen-containing extracellular matrix	1.36E-07	3.93E-05	3.39E-05	46
CC	apical part of cell	5.17E-07	9.93E-05	8.56E-05	45
CC	endoplasmic reticulum lumen	1.26E-06	1.81E-04	1.56E-04	35
CC	apical plasma membrane	7.24E-06	6.23E-04	5.37E-04	37
CC	fibrillar collagen trimer	7.57E-06	6.23E-04	5.37E-04	6
CC	banded collagen fibril	7.57E-06	6.23E-04	5.37E-04	6
CC	basolateral plasma membrane	1.38E-05	8.82E-04	7.61E-04	28
CC	complex of collagen trimers	3.17E-05	1.83E-03	1.58E-03	7
CC	cell projection membrane	3.56E-05	1.87E-03	1.61E-03	34
CC	transmembrane transporter complex	5.39E-05	2.46E-03	2.12E-03	32
CC	transporter complex	8.60E-05	2.86E-03	2.46E-03	32
CC	lysosomal membrane	8.86E-05	2.86E-03	2.46E-03	35
CC	lytic vacuole membrane	8.86E-05	2.86E-03	2.46E-03	35
CC	postsynaptic specialization	9.32E-05	2.86E-03	2.46E-03	34
CC	anchored component of plasma membrane	9.42E-05	2.86E-03	2.46E-03	11
CC	lateral plasma membrane	1.99E-04	5.45E-03	4.70E-03	11
CC	vacuolar membrane	2.54E-04	6.66E-03	5.74E-03	37
CC	cell-cell junction	2.87E-04	7.20E-03	6.21E-03	41
CC	synaptic membrane	3.47E-04	8.32E-03	7.17E-03	34
CC	lamellipodium membrane	4.08E-04	9.10E-03	7.85E-03	6
CC	ion channel complex	4.11E-04	9.10E-03	7.85E-03	28
CC	collagen trimer	7.29E-04	1.55E-02	1.34E-02	12
CC	microvillus membrane	1.07E-03	2.13E-02	1.84E-02	6
CC	sodium channel complex	1.07E-03	2.13E-02	1.84E-02	6
CC	postsynaptic density membrane	1.20E-03	2.31E-02	1.99E-02	12
CC	cortical cytoskeleton	1.37E-03	2.54E-02	2.19E-02	13
CC	postsynaptic membrane	1.60E-03	2.88E-02	2.49E-02	25
CC	glutamatergic synapse	1.81E-03	3.15E-02	2.72E-02	30
CC	aggresome	2.01E-03	3.40E-02	2.94E-02	7
CC	cell body membrane	2.81E-03	4.62E-02	3.99E-02	6
CC	histone deacetylase complex	3.10E-03	4.95E-02	4.27E-02	10
CC	basement membrane	3.18E-03	4.95E-02	4.27E-02	12
MF	metal ion transmembrane transporter activity	5.61E-07	2.47E-04	2.20E-04	46
MF	ion channel activity	1.99E-05	2.12E-03	1.89E-03	42

MF	calcium ion transmembrane transporter activity	3.08E-05	2.14E-03	1.90E-03	19
MF	gated channel activity	4.36E-06	7.86E-04	6.99E-04	37
MF	semaphorin receptor activity	1.38E-08	1.25E-05	1.11E-05	8
MF	extracellular matrix structural constituent	8.22E-07	2.47E-04	2.20E-04	25
MF	ligand-gated calcium channel activity	2.38E-06	5.36E-04	4.77E-04	9
MF	divalent inorganic cation transmembrane transporter activity	1.51E-05	2.12E-03	1.89E-03	22
MF	cation channel activity	2.15E-05	2.12E-03	1.89E-03	35
MF	extracellular matrix structural constituent conferring tensile strength	2.20E-05	2.12E-03	1.89E-03	10
MF	Notch binding	2.35E-05	2.12E-03	1.89E-03	8
MF	cell adhesion mediator activity	2.95E-05	2.14E-03	1.90E-03	12
MF	cell-cell adhesion mediator activity	3.00E-05	2.14E-03	1.90E-03	11
MF	channel activity	5.30E-05	2.93E-03	2.60E-03	44
MF	passive transmembrane transporter activity	5.57E-05	2.93E-03	2.60E-03	44
MF	transcription coactivator activity	5.61E-05	2.93E-03	2.60E-03	29
MF	voltage-gated cation channel activity	5.69E-05	2.93E-03	2.60E-03	19
MF	adenylate cyclase activity	5.84E-05	2.93E-03	2.60E-03	5
MF	calcium channel activity	6.47E-05	3.07E-03	2.73E-03	17
MF	calcium-release channel activity	1.08E-04	4.88E-03	4.34E-03	6
MF	calmodulin binding	1.37E-04	5.88E-03	5.23E-03	23
MF	voltage-gated calcium channel activity	2.43E-04	9.96E-03	8.86E-03	9
MF	amyloid-beta binding	3.59E-04	1.40E-02	1.25E-02	12
MF	growth factor binding	3.74E-04	1.40E-02	1.25E-02	17
MF	intracellular ligand-gated ion channel activity	4.11E-04	1.42E-02	1.27E-02	7
MF	laminin binding	4.11E-04	1.42E-02	1.27E-02	7
MF	active transmembrane transporter activity	4.30E-04	1.44E-02	1.28E-02	32
MF	ATPase activity	5.25E-04	1.69E-02	1.50E-02	37
MF	semaphorin receptor binding	6.85E-04	2.12E-02	1.88E-02	6
MF	voltage-gated ion channel activity	7.28E-04	2.12E-02	1.88E-02	21
MF	voltage-gated channel activity	7.28E-04	2.12E-02	1.88E-02	21
MF	ligand-gated cation channel activity	8.31E-04	2.34E-02	2.08E-02	14
MF	sodium channel activity	1.22E-03	3.33E-02	2.96E-02	8
MF	sodium ion transmembrane transporter activity	1.33E-03	3.54E-02	3.15E-02	17
MF	platelet-derived growth factor binding	1.45E-03	3.75E-02	3.33E-02	4

Supplementary Table 5. Differentially expressed lncRNAs in AD and NC EVs from the post-mortem frontal cortex.

geneID	Fold Change (FC)	log_2FC	*P*-Value
lnc-STOM	69.39204605	6.116698401	0.028891466
OTUD6B-AS1	46.14639608	5.528146076	0.040167162
lnc-USP6	15.18505264	3.924580004	0.02638773
WDFY3-AS2	12.54388714	3.648912579	0.049000463
lnc-HSPA12A	5.292140632	2.4038514	0.013976298
MIR29B2CHG	5.225321163	2.385519712	0.005044766

SUPPLEMENTARY DATA

lnc-CHI3L1	5.108895807	2.353011513	0.033893029
lnc-TCP1	4.402695888	2.138387195	0.013965943
GAS5	3.621915774	1.856752997	0.019907696
LUCAT1	3.361866044	1.74926224	0.022858289
lnc-PAWR	3.321442213	1.731809814	0.015020735
TMEM161B-AS1	3.236548619	1.694456176	0.02571418
lnc-GLDN	3.006323848	1.588000428	0.042790774
lnc-KDM4C	3.00390443	1.586838914	0.009830289
lnc-COX7C	2.879977582	1.526057582	0.041700897
MIR100HG	2.876292654	1.524210473	0.00516069
lnc-PCSK1	2.85515449	1.513568811	0.016840554
lnc-URGCP-MRPS24	2.742888417	1.455695933	0.010789109
MIR99AHG	2.73645032	1.452305665	0.030444255
lnc-ZIC4	2.690782788	1.428025935	0.042810918
lnc-LYPLAL1	2.68685355	1.425917688	0.014283474
AFF1-AS1	2.653371244	1.407826543	0.040225929
lnc-RPL37	2.539523916	1.34455806	0.011141076
lnc-EPCAM	2.533865044	1.341339687	0.006150258
lnc-TBC1D2B	2.517274748	1.331862688	0.041900694
lnc-TIMM8B	2.463275749	1.300578138	0.033305623
PITPNA-AS1	2.411674554	1.270035234	0.029526614
lnc-FLRT3	2.399951293	1.263005126	0.022086089
lnc-RGMB	2.396212428	1.260755811	0.042981908
SNHG6	2.396191028	1.260742926	0.00953732
lnc-FBXO11	2.38377688	1.253249207	0.021663389
lnc-NEDD8-MDP1	2.353639811	1.234893555	0.009527316
ID2-AS1	2.34591945	1.230153478	0.02110832
lnc-RPL37	2.339241636	1.226040895	0.02289847
CD27-AS1	2.331988153	1.221560459	0.032388794
lnc-FAM110C	2.318619787	1.213266262	0.019795405
lnc-ATP6V1E2	2.243214649	1.165567676	0.0489572
lnc-SNX31	2.230298688	1.157236933	0.02684496
LINC00665	2.217608232	1.149004518	0.047366289
LINC00630	2.207761593	1.14258439	0.036653597
lnc-STAC	2.207277449	1.142267984	0.018826095
lnc-TP53TG3	2.185210601	1.127772327	0.044342366
lnc-ARHGAP24	2.175447592	1.121312262	0.008491692
lnc-ZNF527	2.147696714	1.102790278	0.04861095
lnc-RDM1	2.147294712	1.102520212	0.021048832
lnc-MYL7	2.143196744	1.099764294	0.039479898
lnc-TAF15	2.141984227	1.098947857	0.005705058
lnc-LRRTM4	2.138535039	1.096622846	0.006836938
lnc-C12orf74	2.096062455	1.067681705	0.048584657
lnc-TLK1	2.092372684	1.065139841	0.021923228
lnc-MMADHC	2.086150753	1.060843416	0.045049484
lnc-C18orf32	2.075553542	1.053496149	0.042799547

© 2022. Luo D et al. Published online at http://www.aginganddisease.org/EN/10.14336/AD.2022.0607

lnc-BRF1	2.072496747	1.051369837	0.048142426
lnc-ATP1B2	2.016949563	1.012175008	0.047448744
lnc-GALNT18	2.007263972	1.005230356	0.030268563
NNT-AS1	1.988179215	0.991447808	0.020166275
lnc-MNX1	1.985074995	0.989193512	0.034633341
lnc-BDH1	1.982309149	0.987181974	0.030356021
lnc-PLPP5	1.975228675	0.982019686	0.025714026
lnc-LRP12	1.969636899	0.977929694	0.045975562
lnc-DMC1	1.948101264	0.962068672	0.00506256
lnc-KANK4	1.938413768	0.954876557	0.043408802
lnc-PEX10	1.925778666	0.945441901	0.016861355
lnc-DDIT3	1.901829973	0.927388273	0.03670067
lnc-PDE4DIP	1.900593203	0.926449775	0.027607573
LINC01285	1.893746755	0.921243416	0.034913416
lnc-PAWR	1.893140268	0.920781308	0.044663395
lnc-POLDIP3	1.87273162	0.905144163	0.026684052
lnc-HAND1	1.858982537	0.894513218	0.040602303
lnc-RBM22	1.855293602	0.891647513	0.034937011
lnc-TTPAL	1.846831738	0.885052431	0.023392335
lnc-TCEA1	1.735494835	0.795347071	0.041692909
lnc-SCYL3	1.734715794	0.794699319	0.046021016
lnc-POLDIP2	1.729306173	0.79019332	0.047003822
lnc-AMMECR1L	1.587935986	0.667152755	0.046607095
lnc-ACTN2	0.589859494	-0.761556753	0.046993364
lnc-TTC36	0.578246654	-0.790243081	0.042544792
lnc-CCDC32	0.57211246	-0.805629329	0.039743545
lnc-HEATR4	0.570695994	-0.809205659	0.048662524
lnc-GTF2H2	0.567998131	-0.816041913	0.02030028
lnc-ANKRD20A4	0.560731553	-0.834617841	0.047638109
FAM66B	0.547808921	-0.868255334	0.040331766
lnc-H6PD	0.5423827	-0.882616932	0.049666179
lnc-NUDT9	0.541833946	-0.884077315	0.038764261
lnc-RGS8	0.537788318	-0.894889679	0.021588852
lnc-LRRC63	0.53371029	-0.905871268	0.029399857
STX18-AS1	0.531809662	-0.911018107	0.048988596
lnc-SULF1	0.51740179	-0.950643051	0.025081503
lnc-RANBP9	0.513475586	-0.961632411	0.047406428
lnc-SYNPO	0.512214977	-0.965178658	0.035862976
DDX11-AS1	0.512134049	-0.965406615	0.029485101
lnc-FOXA1	0.50862626	-0.975322144	0.039505214
lnc-CANX	0.505806764	-0.983341764	0.040552405
lnc-GOLGA6L22	0.504482204	-0.987124717	0.034242585
lnc-ZNF212	0.499705663	-1.000849526	0.029896706
lnc-SLC25A6	0.498634175	-1.003946332	0.035815081
lnc-SEC11A	0.495141223	-1.01408803	0.013811076
lnc-WDR73	0.495141223	-1.01408803	0.013811076

© 2022. Luo D et al. Published online at http://www.aginganddisease.org/EN/10.14336/AD.2022.0607

lnc-NOS1	0.494952154	-1.014639025	0.042210023
lnc-BRCA1	0.494445405	-1.016116862	0.044296624
LINC01105	0.492483248	-1.021853443	0.033227339
lnc-RPL19	0.492272906	-1.022469757	0.030548776
lnc-CCDC167	0.488012528	-1.03500991	0.044672688
lnc-GOLGA7B	0.485721789	-1.041797891	0.024014195
LINC00937	0.484337713	-1.045914753	0.032351236
LINC01000	0.482472894	-1.051480202	0.026335427
lnc-SETD7	0.481667199	-1.053891414	0.017653104
lnc-TXNDC15	0.480631248	-1.056997647	0.01924177
lnc-BID	0.477797577	-1.065528558	0.042494659
lnc-PITX1	0.474177395	-1.076501207	0.029785229
lnc-WFS1	0.473105057	-1.079767513	0.013559685
lnc-TF	0.471706104	-1.084039825	0.011197097
lnc-HIP1	0.468269813	-1.094588056	0.047876684
OIP5-AS1	0.466460359	-1.100173614	0.029236299
lnc-PSPC1	0.462103537	-1.113711963	0.010693082
lnc-FGL2	0.461917666	-1.114292373	0.028884604
lnc-ITPR3	0.461449358	-1.115755768	0.028027673
lnc-JADE1	0.454997101	-1.136070742	0.038952049
lnc-SPAG1	0.45346393	-1.140940297	0.035224047
lnc-OR4F29	0.453297104	-1.141471149	0.016355808
lnc-SLC6A18	0.452653786	-1.143520075	0.030222585
lnc-FNDC1	0.452604216	-1.143678072	0.016042509
lnc-TFAP4	0.448822473	-1.155783179	0.035023634
lnc-SAMD11	0.446582833	-1.1630003	0.031110801
lnc-RUBCN	0.445854393	-1.165355462	0.018705383
lnc-HMX1	0.444263916	-1.170511127	0.011415861
lnc-MAP2K3	0.440990096	-1.181181839	0.028752562
lnc-DIP2A	0.43944544	-1.186244038	0.022987047
lnc-DNASE1L1	0.437902388	-1.191318776	0.005980619
lnc-RBM19	0.436258321	-1.196745446	0.025516922
TSPEAR-AS2	0.435344901	-1.19976927	0.021229369
TSIX	0.434731369	-1.201803894	0.01393024
lnc-SLC7A1	0.432978448	-1.207632881	0.028331571
lnc-NPIPB4	0.428907603	-1.221261206	0.025394988
lnc-PTS	0.428751237	-1.221787262	0.043921217
lnc-MICALL2	0.428743655	-1.221812774	0.035642199
LINC-PINT	0.428423686	-1.22288985	0.037143518
lnc-DRICH1	0.425358354	-1.233249306	0.025503797
lnc-CDH5	0.425256499	-1.233594811	0.014857493
lnc-C1orf61	0.42440023	-1.236502656	0.011090542
lnc-STX5	0.422403756	-1.243305432	0.038593225
FAM66B	0.420805865	-1.248773282	0.007425969
lnc-SBDS	0.420417331	-1.250105951	0.020648237
lnc-PDPK1	0.418820302	-1.255596718	0.025530813

LINC01714	0.41785638	-1.25892093	0.045280557
lnc-TMUB2	0.417395294	-1.260513761	0.023206453
lnc-VPS33B	0.416934618	-1.262106932	0.044785944
lnc-CRYM	0.413976396	-1.272379584	0.01093552
lnc-NEIL3	0.413092068	-1.275464737	0.012149016
lnc-DNASE1L1	0.412973939	-1.275877354	0.012324288
lnc-AHDC1	0.406872056	-1.297352894	0.033475692
lnc-TACR1	0.404799356	-1.3047211	0.010068985
lnc-CRYBA4	0.404153476	-1.30702484	0.023736902
lnc-DPF3	0.40191433	-1.315040079	0.018136326
lnc-CANT1	0.40163872	-1.316029735	0.048695089
lnc-TNK2	0.401012419	-1.318281177	0.024130512
lnc-MPO	0.393998172	-1.343739158	0.037121889
lnc-SAMD11	0.391441076	-1.35313294	0.013868976
lnc-DNAL4	0.388932652	-1.362407735	0.042036283
lnc-SH3D21	0.385789541	-1.374114063	0.03748518
MIR3681HG	0.38530234	-1.375937148	0.049016719
SRP14-AS1	0.384708678	-1.378161723	0.008255445
lnc-RAB28	0.384478212	-1.379026249	0.008839089
lnc-ZNF717	0.382158222	-1.387758023	0.030725408
lnc-MRPL20	0.379616188	-1.397386582	0.002487448
lnc-SLC22A5	0.378970629	-1.399842055	0.010673957
lnc-LAT	0.377659499	-1.40484202	0.017707163
lnc-PDYN	0.376314796	-1.409988081	0.038223044
lnc-BTD	0.375167567	-1.414392982	0.028518292
lnc-OR4F16	0.371887777	-1.427060765	0.02182106
lnc-NCOA4	0.367827691	-1.442898	0.009561774
lnc-ZNF717	0.367677766	-1.443486156	0.03114621
lnc-SYF2	0.366664964	-1.447465676	0.009850684
NEAT1	0.366587026	-1.447772368	0.01354888
lnc-HAPLN3	0.363760413	-1.458939547	0.007609879
LINC00982	0.363423872	-1.460274906	0.009090563
lnc-MYO15B	0.361115792	-1.469466581	0.018754499
lnc-GPR65	0.361109297	-1.46949253	0.018469785
lnc-MYO1F	0.360893077	-1.470356628	0.03187249
lnc-C1orf61	0.360363679	-1.472474485	0.031222572
lnc-SRGAP2C	0.35998742	-1.473981602	0.01642673
lnc-BTBD10	0.359298859	-1.476743739	0.031239593
lnc-ANKRD20A2	0.357094066	-1.485623936	0.027309367
lnc-AKAP1	0.356954806	-1.486186667	0.005675848
lnc-CCDC51	0.355969801	-1.49017324	0.011454507
lnc-UBE3A	0.355803818	-1.490846105	0.016197572
lnc-ST8SIA4	0.355603086	-1.491660252	0.012821495
lnc-MYO16	0.354064443	-1.497916127	0.010381139
lnc-TSPAN14	0.352687189	-1.503538924	0.008598101
lnc-LMF1	0.352022747	-1.506259438	0.049162985

© 2022. Luo D et al. Published online at http://www.aginganddisease.org/EN/10.14336/AD.2022.0607

lnc-SAMD11	0.347928524	-1.523137134	0.031556714
lnc-CLEC18B	0.346056562	-1.530920233	0.049446064
lnc-PLXNA4	0.345862366	-1.531730056	0.012792774
lnc-PITX1	0.345281614	-1.534154582	0.030544424
MIR29B2CHG	0.344303762	-1.538246148	0.009996994
lnc-WARS	0.343350082	-1.542247785	0.042776036
lnc-C17orf62	0.339967941	-1.556529387	0.002835641
lnc-RAB43	0.33829518	-1.563645474	0.019991131
lnc-MTMR10	0.336442019	-1.571570195	0.005191289
lnc-PSEN2	0.335861867	-1.574060088	0.007741691
lnc-OLFM4	0.334411039	-1.580305625	0.020967888
lnc-TIMP3	0.334167731	-1.581355669	0.015824717
lnc-C2orf81	0.332158541	-1.590056084	0.007195665
lnc-GOLGA6L6	0.329794354	-1.600361395	0.00470624
lnc-FOXD4L5	0.328338516	-1.606744097	0.031849264
lnc-STX17	0.327468057	-1.610573908	0.01813161
lnc-ZNF705E	0.32456339	-1.623427818	0.027460328
LINC01771	0.318533219	-1.65048426	0.010501088
lnc-MUC4	0.317969419	-1.653040074	0.041828733
SLC2A1-AS1	0.317840525	-1.653625013	0.005235294
LINC02449	0.31560408	-1.663812241	0.002295479
lnc-POLDIP3	0.315060707	-1.666298255	0.011154766
lnc-PRR32	0.314515482	-1.66879706	0.036303748
lnc-FAM72B	0.309364153	-1.692622059	0.030323523
lnc-CHRFAM7A	0.30785866	-1.699659943	0.004803581
MIRLET7BHG	0.305395492	-1.711249327	0.017302947
lnc-CCDC92	0.301712153	-1.728755285	0.01623415
lnc-ZNF727	0.295587143	-1.758344574	0.023532284
lnc-CCDC92	0.295294958	-1.759771371	0.002347182
lnc-NPIPA7	0.295253934	-1.75997181	0.027219701
lnc-ZNF33B	0.295035216	-1.761040927	0.006321867
lnc-MICALL2	0.294639981	-1.762974888	0.043672399
lnc-PFKFB3	0.29256558	-1.773168047	0.003272125
lnc-AKAP8	0.292476514	-1.773607316	0.005434558
lnc-KCNQ2	0.291983105	-1.776043204	0.016635424
lnc-MACC1	0.290946794	-1.781172744	0.006525904
lnc-PDCD2L	0.290063585	-1.785558904	0.008692107
LINC00174	0.289479189	-1.788468458	0.012024671
lnc-ZDHHC11B	0.289420351	-1.788761724	0.010136807
lnc-HIST1H4J	0.289180392	-1.789958359	0.000253931
LINC01089	0.289135017	-1.79018475	0.012790642
lnc-FAM72B	0.288954315	-1.791086682	0.012978823
lnc-ADGRB2	0.288467518	-1.793519215	0.033987073
lnc-PDYN	0.288377982	-1.793967079	0.011655487
lnc-METTL14	0.286359528	-1.804100489	0.005210806
lnc-STK39	0.284529627	-1.813349211	0.004554882

lnc-SLITRK5	0.282608354	-1.823123982	0.000887988
CARMN	0.276681205	-1.853703452	0.005998503
lnc-PRIM2	0.276496233	-1.854668271	0.006257973
lnc-SCARB1	0.27463429	-1.864416326	0.026645103
lnc-CRACR2A	0.272714559	-1.874536373	0.010648569
lnc-CRYBA4	0.269417642	-1.892083771	0.012440978
lnc-MUC15	0.268923365	-1.894732988	0.034342217
lnc-MYBPC2	0.267004406	-1.905064545	0.017352021
lnc-NR1D1	0.26694048	-1.905409998	0.016896186
lnc-GGCT	0.265755774	-1.911827059	0.037215341
lnc-ZNF705E	0.25509137	-1.970914002	0.024461889
lnc-TNNT1	0.254951042	-1.97170786	0.035899069
lnc-ZNF33B	0.253376554	-1.980645065	0.001144917
lnc-ZNF33B	0.253371633	-1.980673084	0.032065679
lnc-SH3BP5L	0.252404538	-1.986190246	0.012021597
lnc-PCDH8	0.251972462	-1.988662024	0.021922724
lnc-FSCN2	0.251001031	-1.994234803	0.002047964
lnc-SSPO	0.249243081	-2.004374637	0.007560096
lnc-NMU	0.248112377	-2.010934388	0.028076134
lnc-TBC1D3H	0.246326472	-2.021356414	0.012149521
lnc-MYF5	0.245529584	-2.02603123	0.001156388
lnc-ZNF146	0.245037623	-2.028924816	0.004651006
FTX	0.244268925	-2.033457752	0.003144579
lnc-NTHL1	0.240413823	-2.056408247	0.002933184
LINC00599	0.237859185	-2.071820361	0.038627745
lnc-ADORA1	0.23703682	-2.076816917	0.042328755
CD27-AS1	0.233980635	-2.095538963	0.024733487
lnc-GOLGA6L22	0.233185528	-2.100449841	0.004914354
GATA2-AS1	0.231733702	-2.109460217	0.038420879
lnc-ZNF680	0.231505944	-2.110878858	0.007412901
lnc-MROH7	0.228828798	-2.12765947	0.013993238
CYP1B1-AS1	0.225535379	-2.148574336	0.006432082
lnc-DLK1	0.224439269	-2.155602979	0.040774757
lnc-KCNS2	0.223932994	-2.158860989	0.014533844
lnc-TP53BP2	0.223752925	-2.160021552	0.001061329
lnc-AMZ2	0.223070957	-2.164425399	0.020368677
lnc-RNASE1	0.222867855	-2.165739546	0.000283692
NRSN2-AS1	0.222440511	-2.168508536	0.036150207
lnc-CD5	0.221695215	-2.173350461	0.002371652
lnc-DLK1	0.220819894	-2.17905794	0.043328112
lnc-LINC02210-CRHR1	0.218031515	-2.197391413	0.002318282
FAM95B1	0.214121157	-2.223500741	0.039682611
lnc-BPIFC	0.212163446	-2.236751981	0.025381051
lnc-SSH3	0.211891879	-2.238599798	0.049140663
lnc-SPATA19	0.211007474	-2.244633992	0.022495093
lnc-DHRS7B	0.2085115	-2.261801142	0.000419115

© 2022. Luo D et al. Published online at http://www.aginganddisease.org/EN/10.14336/AD.2022.0607

lnc-NLGN2	0.206425058	-2.276309984	0.026998379
lnc-ZNF33B	0.205687278	-2.28147553	0.004308723
lnc-GPR158	0.204687137	-2.288507652	3.13973E-05
lnc-AKAP8	0.202867101	-2.301393173	0.028894313
lnc-SLC16A7	0.198765276	-2.330862351	0.004141892
lnc-ANKRD18B	0.196918953	-2.344326119	5.32501E-05
lnc-DLK1	0.19575004	-2.352915491	0.004658231
lnc-DNAJC5	0.19443488	-2.362641044	0.015118688
lnc-TNK2	0.18852641	-2.407161458	0.004319843
lnc-ANKRD20A4	0.188071514	-2.410646742	0.037427504
LINC02142	0.182329009	-2.455383977	0.008365697
LINC-PINT	0.179611942	-2.477044816	0.02937325
lnc-METTL14	0.175612695	-2.509530953	0.005705398
lnc-BMS1	0.17427961	-2.520524303	0.001772686
lnc-BMS1	0.172268178	-2.537271871	0.002793078
lnc-PILRB	0.171915955	-2.540224648	0.001783036
lnc-CASTOR3	0.159905844	-2.644705426	0.000717316
lnc-MUC20	0.15458925	-2.6934881	0.017812232
lnc-COBL	0.14940929	-2.742658243	0.004291227
lnc-CPSF7	0.148919183	-2.747398488	0.014991973
lnc-NOMO2	0.148210627	-2.754279198	0.021563895
NEAT1	0.145200304	-2.783883621	0.017809931
KCNQ1OT1	0.141311085	-2.82305345	0.000140036
lnc-PDCD2L	0.140783204	-2.82845287	0.014204528
LINC01106	0.131553946	-2.926273569	0.049427502
lnc-PDYN	0.123649923	-3.015666752	0.001364467
lnc-OR4F29	0.119618911	-3.063482606	0.000322456
MIR29B2CHG	0.110621125	-3.176301178	0.036539026
lnc-CAPN15	0.105468673	-3.245113555	0.011356326
lnc-CAPN15	0.085556027	-3.546986702	0.009569641
lnc-HSPA12A	0.085472438	-3.548396924	0.014339182
LINC01000	0.084492363	-3.56503525	0.000769379
LINC00599	0.074683431	-3.743067975	0.032726303
LINC00599	0.074683431	-3.743067975	0.032726303
FGD5-AS1	0.065703116	-3.927894398	0.018323751
LINC01963	0.044927741	-4.476249682	0.044596655
lnc-CRYBA4	0.031911499	-4.969779815	0.006453699
lnc-LPCAT1	0.028540799	-5.13083045	3.55135E-06

Supplementary Table S6. Functional analysis of *cis*-regulated genes of DElncRNAs.

Type	Description	*P*-value	-log10*P*	Count
BP	regulation of metal ion transport	0.00062	3.207622	13

© 2022. Luo D et al. Published online at http://www.aginganddisease.org/EN/10.14336/AD.2022.0607

BP	cellular calcium ion homeostasis	0.0065097	2.186438	12
BP	regulation of ion transmembrane transport	0.0097081	2.012864	12
BP	regulation of cation transmembrane transport	0.0019673	2.70613	11
BP	regulation of cytosolic calcium ion concentration	0.0080859	2.092272	10
BP	regulation of ion transmembrane transporter activity	0.0030698	2.512891	9
BP	neuron death	0.0467306	1.330399	8
BP	regulation of cation channel activity	0.0046752	2.330203	7
BP	regulation of potassium ion transport	0.0012949	2.887757	6
BP	regulation of synapse organization	0.0392546	1.40611	6
BP	neuron fate commitment	0.0064793	2.188474	4
BP	positive regulation of synapse assembly	0.0075906	2.119721	4
BP	activation of GTPase activity	0.0250638	1.600953	4
CC	presynapse	0.0125494	1.901377	12
CC	secretory granule lumen	0.0013843	2.858771	11
CC	neuronal cell body	0.0311963	1.505896	11
CC	apical part of cell	0.0146436	1.834351	10
CC	synaptic membrane	0.0300282	1.522471	10
CC	postsynaptic membrane	0.0135119	1.869283	9
CC	specific granule	0.0005761	3.239517	8
CC	neuron spine	0.0028011	2.552672	7
CC	presynaptic membrane	0.0114184	1.942394	6
CC	intrinsic component of synaptic membrane	0.0124212	1.905837	6
CC	RNA polymerase II transcription regulator complex	0.0131231	1.881962	6
CC	axon terminus	0.0126873	1.896631	5
CC	intrinsic component of postsynaptic membrane	0.0140067	1.853663	5
CC	neuron projection terminus	0.0226079	1.645739	5
CC	myosin complex	0.0260614	1.584002	3
CC	azurophil granule membrane	0.0298915	1.524452	3
CC	sarcoplasmic reticulum	0.0496851	1.303774	3
MF	actin binding	0.0119897	1.921192	11
MF	phosphatidylinositol binding	0.024307	1.614269	7
MF	molecular adaptor activity	0.0288189	1.540323	7
MF	protein-macromolecule adaptor activity	0.0337391	1.471867	6
MF	ion channel binding	0.0147483	1.831257	5
MF	NADP binding	0.0030665	2.513363	4
MF	SNAP receptor activity	0.0054232	2.265747	3
MF	metal cluster binding	0.0366299	1.436165	3
MF	LRR domain binding	0.0161997	1.790494	2
MF	apolipoprotein binding	0.0161997	1.790494	2
MF	NF-kappaB binding	0.0441826	1.354748	2
KEGG	SNARE interactions in vesicular transport	0.008253	2.083387	3
KEGG	Phagosome	0.0110339	1.957271	6
KEGG	Amyotrophic lateral sclerosis (ALS)	0.0247211	1.606932	3
KEGG	Protein export	0.0321955	1.492206	2
KEGG	Vitamin digestion and absorption	0.0348449	1.45786	2

SUPPLEMENTARY DATA

Supplementary Table 7. Differentially expressed circRNAs in AD and NC EVs from the post-mortem frontal cortex.

circbaseID	Gene	Fold Change (FC)	log_2FC	*P*-Value
hsa_circ_0031668	RALGAPA1	13.97065999	3.804328272	0.002481502
hsa_circ_0000660	MCTP2	10.11446129	3.338347577	0.002723266
hsa_circ_0116552	DDX17	8.75563265	3.130211426	0.011522232
hsa_circ_0002580	NR1D2	8.054504326	3.009795808	0.016112826
hsa_circ_0079624	MPP6	8.041879551	3.007532728	0.018957803
hsa_circ_0080955	CROT	7.865721595	2.975579123	0.009966483
hsa_circ_0025763	TMTC1	7.271031347	2.862160015	0.020472205
hsa_circ_0097101	SLC41A2	6.649865853	2.733325238	0.016987535
hsa_circ_0006166	RGPD2	6.623602376	2.727616068	0.00354068
hsa_circ_0031485	HECTD1	6.522364413	2.705395049	0.01063423
hsa_circ_0006832	DYRK1A	6.501912816	2.700864211	0.017740098
hsa_circ_0031460	HECTD1	6.446123798	2.688431896	0.047189438
hsa_circ_0046882	PPP4R1	6.278671503	2.650459333	0.031809477
hsa_circ_0008343	ARIH2	5.89670111	2.559908069	0.015718604
hsa_circ_0129130	DHX29	5.700810368	2.511167012	0.048612102
hsa_circ_0040039	SNTB2	5.620991273	2.490824575	0.049936361
hsa_circ_0089973	REPS2	5.612884042	2.488742254	0.027117363
hsa_circ_0092785	ATRNL1	5.602212043	2.48599659	0.011277651
hsa_circ_0033413	TECPR2	5.417371635	2.437593064	0.013771741
hsa_circ_0035947	DENND4A	5.401557429	2.433375439	0.043645871
hsa_circ_0097666	CCDC62	5.183143148	2.373827238	0.031200352
hsa_circ_0038487	EEF2K	5.151904567	2.365105869	0.03337664
hsa_circ_0055066	NFU1	4.984388133	2.317416415	0.043801733
hsa_circ_0003588	MKLN1	4.969473209	2.313092927	0.047158853
hsa_circ_0049291	SLC44A2	4.910107942	2.295754741	0.035235277
hsa_circ_0114422	ZNF644	4.9020651	2.293389644	0.040254882
hsa_circ_0000184	KCTD3	4.880015892	2.286885846	0.010845212
hsa_circ_0133631	CNTNAP2	4.865715413	2.282651942	0.029195088
hsa_circ_0095337	ARHGAP32	4.824117352	2.270265003	0.038947023
hsa_circ_0119529	FAM228B	4.812423781	2.266763692	0.045574882
hsa_circ_0116743	EFCAB6	4.589449729	2.198321186	0.04691769
hsa_circ_0006292	DHRSX	4.250491059	2.087629525	0.00952143
hsa_circ_0095579	PRMT3	4.172421579	2.060884934	0.014991884
hsa_circ_0003448	ZNF410	3.982681143	1.993739981	0.017425366
hsa_circ_0004342	AFDN	3.900286492	1.9635801	0.043352847
hsa_circ_0001092	CFLAR	3.876644271	1.954808356	0.02128861
hsa_circ_0002713	RANBP17	3.837514738	1.940172291	0.033850095
hsa_circ_0003716	SEC31A	3.664924609	1.873783521	0.041459173
hsa_circ_0006479	TEX2	3.550159725	1.827883934	0.03220456
hsa_circ_0140490	OPHN1	3.451476636	1.787213718	0.048333035
hsa_circ_0131667	not_annotated	3.389311204	1.76099211	0.047240649

© 2022. Luo D et al. Published online at http://www.aginganddisease.org/EN/10.14336/AD.2022.0607

SUPPLEMENTARY DATA

hsa_circ_0139233	NAA35	3.290939495	1.718499502	0.013056807
hsa_circ_0136539	ANK1	3.249296432	1.700127366	0.019607905
hsa_circ_0006931	TXLNG	3.169869237	1.664423328	0.028036897
hsa_circ_0087493	IARS1	3.09017861	1.627690227	0.005962084
hsa_circ_0136407	FUT10	2.953951197	1.562645991	0.036855644
hsa_circ_0004478	FGGY	2.74368318	1.4561139	0.021290306
hsa_circ_0000495	MYCBP2	2.7424823	1.455482309	0.036278476
hsa_circ_0116499	LARGE1	2.695514972	1.430560923	0.043606413
hsa_circ_0108584	ME2	2.627952783	1.393939354	0.028593312
hsa_circ_0063452	MRTFA	2.606443301	1.382082477	0.024595956
hsa_circ_0114307	PRKACB	2.580749567	1.367790151	0.025761288
hsa_circ_0003718	RANBP17	2.486856055	1.314323003	0.024291906
hsa_circ_0074372	ARHGAP26	2.467527378	1.303066092	0.022484828
hsa_circ_0002906	UBR2	2.399363872	1.262651964	0.049253254
hsa_circ_0004619	FAF1	2.269060098	1.18209482	0.024921902
hsa_circ_0000816	FOXK2	2.229133571	1.156483066	0.038131967
hsa_circ_0001538	PAIP2	2.131912582	1.092148282	0.047007029
hsa_circ_0004576	PRKCA	2.111422485	1.078215283	0.03524096
hsa_circ_0078966	C6orf136	2.10878751	1.07641373	0.041836973
hsa_circ_0005882	STK39	2.09129113	1.064393915	0.012632651
hsa_circ_0114304	PRKACB	2.01793721	1.012881284	0.030894111
hsa_circ_0006364	ZNF81	2.015884749	1.01141316	0.028858725
hsa_circ_0007827	PAFAH1B2	1.981981648	0.986943604	0.040662474
hsa_circ_0111925	RPS6KC1	1.835143934	0.875893221	0.044295381
hsa_circ_0101812	not_annotated	1.717985449	0.780717817	0.047095847
hsa_circ_0000344	RSF1	0.5009505	-0.99726004	0.025227832
hsa_circ_0003658	ZNF365	0.487330056	-1.037028892	0.048043919
hsa_circ_0007365	RPRD1B	0.476278891	-1.070121486	0.022773932
hsa_circ_0139671	not_annotated	0.472578662	-1.081373604	0.037637832
hsa_circ_0127522	MAN2A1	0.472360437	-1.08203996	0.029870574
hsa_circ_0084464	PRKDC	0.448879592	-1.155599588	0.046475486
hsa_circ_0005889	PDE8A	0.436965748	-1.194407899	0.026387681
hsa_circ_0112551	GPR137B	0.431081133	-1.213968672	0.036274126
hsa_circ_0138394	DENND4C	0.429941761	-1.217786846	0.033962135
hsa_circ_0070680	ZGRF1	0.408578342	-1.291315363	0.017613881
hsa_circ_0009029	ZNF462	0.389594474	-1.359954881	0.013951598
hsa_circ_0002457	ATXN2	0.378979386	-1.399808719	0.007801914
hsa_circ_0010117	SPEN	0.362763491	-1.462898826	0.011347135
hsa_circ_0067755	DHX36	0.335161726	-1.577070685	0.041684444
hsa_circ_0008953	SEPTIN10	0.325624707	-1.618717927	0.043966669
hsa_circ_0025853	DNM1L	0.309012066	-1.694264922	0.046656365
hsa_circ_0002894	SYNE1	0.295129493	-1.760579996	0.021570096
hsa_circ_0006611	PLEKHA5	0.293049291	-1.77078475	0.01886779
hsa_circ_0003272	AGAP1	0.291528895	-1.778289211	0.010077935
hsa_circ_0103578	ADAL	0.283122357	-1.820502417	0.044854561
hsa_circ_0007308	PDS5A	0.273173143	-1.872112445	0.001950725

© 2022. Luo D et al. Published online at http://www.aginganddisease.org/EN/10.14336/AD.2022.0607

hsa_circ_0132107	ADGRB3	0.272675816	-1.874741346	0.00709305
hsa_circ_0100439	COG6	0.270917478	-1.884074626	0.03624774
hsa_circ_0112501	GGPS1	0.255017	-1.971334672	0.048373171
hsa_circ_0008776	THSD1	0.237515253	-2.073907933	0.021980532
hsa_circ_0121601	STXBP5L	0.236850864	-2.077949162	0.040588893
hsa_circ_0004670	BAZ1B	0.23032856	-2.118234785	0.03291593
hsa_circ_0117141	C2orf76	0.205629653	-2.281879769	0.027123232
hsa_circ_0074370	ARHGAP26	0.196281092	-2.349006889	0.048974344
hsa_circ_0006844	GLT8D2	0.193255046	-2.371422009	0.049899044
hsa_circ_0009007	RABEPK	0.191661247	-2.383369436	0.037754487
hsa_circ_0105799	ZFP90	0.1906835	-2.390748085	0.039364861
hsa_circ_0009140	SCFD1	0.182350412	-2.455214636	0.006321931
hsa_circ_0126620	TMEM165	0.177986645	-2.490159097	0.013082962
hsa_circ_0123057	SENP2	0.174418808	-2.519372476	0.029998552
hsa_circ_0005625	CCT4	0.169615984	-2.55965596	0.023349876
hsa_circ_0108630	POLI	0.153976869	-2.699214451	0.036305793
hsa_circ_0135376	AZIN1	0.14074885	-2.82880496	0.036981964
hsa_circ_0030004	SMAD9	0.12536925	-2.995744563	0.03000479
hsa_circ_0102923	CPSF2	0.123255509	-3.020275964	0.007373241
hsa_circ_0050547	UBA2	0.121640935	-3.039299278	0.009048843
hsa_circ_0126506	LOC101928279	0.113267896	-3.142189083	0.017711751
hsa_circ_0100746	SUGT1	0.111102954	-3.170030923	0.01523035
hsa_circ_0140255	SYTL5	0.108649217	-3.202250313	0.016454128
hsa_circ_0002115	ZNF528	0.107497376	-3.21762665	0.015147558
hsa_circ_0014391	UBAP2L	0.096422424	-3.37448749	0.01215569
hsa_circ_0135423	RIMS2	0.093479636	-3.41920408	0.00778156
hsa_circ_0031977	DDHD1	0.091603629	-3.448451429	0.006149884
hsa_circ_0113947	WDR78	0.090668222	-3.463259191	0.011571228
hsa_circ_0056558	R3HDM1	0.084877256	-3.558478168	0.007695173
hsa_circ_0027244	R3HDM2	0.084418297	-3.566300472	0.008885087
hsa_circ_0089902	OFD1	0.08242491	-3.600775778	0.007455301
hsa_circ_0133169	PTPRZ1	0.051862074	-4.269176276	0.00244701

Supplementary Table 8. Functional analysis of *cis*-regulated genes of DEcircRNAs.

Type	Description	*P*-value	-log10*P*	Count
BP	regulation of GTPase activity	5.95172E-05	4.225357348	11
BP	positive regulation of nervous system development	8.48302E-05	4.071449646	8
BP	regulation of dendrite morphogenesis	0.000408111	3.389221591	4
BP	positive regulation of neurogenesis	0.000166753	3.77792591	7
BP	dendrite morphogenesis	0.000908844	3.041510667	5
BP	dendritic spine organization	0.000983577	3.007191512	4
BP	regulation of neurotransmitter secretion	0.001124781	2.94893207	4
BP	neuron projection organization	0.001448811	2.838988214	4

© 2022. Luo D et al. Published online at http://www.aginganddisease.org/EN/10.14336/AD.2022.0607

BP	postsynapse organization	0.001744966	2.758213039	5
BP	dendritic spine development	0.001903122	2.720533325	4
BP	regulation of nervous system development	0.001938003	2.712645633	8
BP	regulation of neurotransmitter transport	0.001975198	2.70438943	4
BP	regulation of neurogenesis	0.002676094	2.57249867	7
BP	regulation of synaptic vesicle exocytosis	0.003194915	2.495540662	3
BP	regulation of small GTPase mediated signal transduction	0.00738927	2.131398449	6
BP	vesicle-mediated transport in synapse	0.020451166	1.689281921	4
BP	synapse organization	0.023389755	1.630974326	6
CC	axolemma	0.001792039	2.74665265	2
CC	cytoplasmic stress granule	0.006975235	2.156441149	3
CC	cytoplasmic ribonucleoprotein granule	0.008350549	2.078284953	5
CC	ribonucleoprotein granule	0.009893682	2.00464206	5
CC	nuclear pore	0.0105805	1.975493793	3
CC	nuclear envelope	0.011814982	1.927566942	7
CC	trans-Golgi network membrane	0.014272931	1.845486847	3
CC	transport vesicle	0.020619289	1.685726316	6
CC	transport vesicle membrane	0.024452923	1.611669226	4
CC	Golgi cisterna	0.025777466	1.588759774	3
CC	chromosomal region	0.037341783	1.427804948	5
CC	neuron projection membrane	0.039515787	1.403229366	2
CC	main axon	0.043228799	1.364226826	2
CC	trans-Golgi network	0.045319677	1.343713192	4
CC	Golgi stack	0.047703678	1.321448131	3
MF	GTPase regulator activity	1.62998E-05	4.787817493	12
MF	GTPase activator activity	3.19736E-05	4.495208736	9
MF	histone kinase activity	0.003767675	2.423926564	2
MF	RNA helicase activity	0.010077513	1.996646639	3
MF	syntaxin binding	0.010077513	1.996646639	3
MF	protein serine/threonine kinase activity	0.012415473	1.906036728	7
MF	helicase activity	0.013088212	1.883119674	4
MF	phospholipid binding	0.015760969	1.802417084	7
MF	carboxy-lyase activity	0.017396542	1.759537059	2
MF	ubiquitin-like protein conjugating enzyme binding	0.021344352	1.670717021	2
MF	transcription coregulator activity	0.021375322	1.670087342	7
MF	translation regulator activity, nucleic acid binding	0.024747836	1.606462771	3
MF	SNARE binding	0.028439756	1.546074139	3
MF	non-membrane spanning protein tyrosine kinase activity	0.02904635	1.536908433	2
MF	carbon-carbon lyase activity	0.035123894	1.454397344	2
MF	magnesium ion binding	0.036707669	1.435243189	4
MF	GTPase binding	0.039945922	1.398527547	4
MF	translation regulator activity	0.045946122	1.337751139	3
KEGG	Wnt signaling pathway	0.005098692	2.292541219	4
KEGG	Insulin secretion	0.006240584	2.20477477	3
KEGG	Morphine addiction	0.007297823	2.136806696	3

KEGG	Vibrio cholerae infection	0.020229278	1.694019623	2
KEGG	Endocrine and other factor-regulated calcium reabsorption	0.022569739	1.646473455	2
KEGG	Oxytocin signaling pathway	0.029218645	1.534339931	3
KEGG	Cortisol synthesis and secretion	0.032968725	1.481897842	2
KEGG	Necroptosis	0.033806277	1.471002653	3
KEGG	Long-term potentiation	0.034854518	1.457740923	2
KEGG	Amphetamine addiction	0.036781647	1.434368825	2
KEGG	Adherens junction	0.038749287	1.411736283	2
KEGG	Thyroid hormone synthesis	0.041774921	1.379084365	2
KEGG	Gastric acid secretion	0.042802843	1.368527388	2

Supplementary Table 9. ECog scales of all donors for WGCNA.

Sample ID	memory	language	visuospatial functions	planning	organize	divided attention	Ecog-average
NC 1	NA	NA	NA	NA	NA	NA	NA
NC 2	NA	NA	NA	NA	NA	NA	NA
NC 3	15	14	12	37	8	4	1.5
NC 4	8	9	7	5	6	4	1
NC 5	8	9	7	5	6	4	1
NC 6	8	9	7	5	6	4	1
NC 7	8	9	7	5	6	4	1
NC 8	8	9	7	5	6	4	1
NC 9	8	9	7	5	6	4	1
NC 10	8	9	7	5	6	4	1
AD 1	8	18	14	5	6	4	1.4
AD 2	34	19	58	27	6	4	2
AD 3	8	12	23	5	6	4	1.1
AD 4	29	26	39	39	36	11	2.9
AD 5	32	32	28	20	24	16	4
AD 6	8	9	7	5	6	4	1
AD 7	32	36	28	20	24	16	4
AD 8	31	35	28	20	24	16	3.9

Supplementary Table 10. Hierarchical clustering of the magenta and lightcyan modules.

geneSymbol	moduleColor
CPXM1	Magenta
TMEFF1	Magenta
MEX3B	Magenta
NEU4	Magenta
lnc-GAS2	Magenta
lnc-LPCAT1	Magenta
lnc-CCDC71L	Magenta
lnc-SPATA31A7	Magenta

CLDN10-AS1	Magenta
lnc-VPS33B	Magenta
lnc-INS-IGF2	Magenta
LINC01376	Magenta
LINC01128	Magenta
EIF1B-AS1	Magenta
LINC01481	Magenta
LACTB2-AS1	Magenta
STK32A-AS1	Magenta
lnc-DCAF12L2	Magenta
FAM111A-DT	Magenta
lnc-C18orf54	Magenta
lnc-BEX2	Magenta
lnc-UBQLN2	Magenta
lnc-CHD9	Magenta
LINC01609	Magenta
LINC01748	Magenta
lnc-MIOS	Magenta
lnc-GRM1	Magenta
lnc-BECN2	Magenta
LINC01725	Magenta
LINC01544	Magenta
lnc-PXDN	Magenta
lnc-NKAP	Magenta
lnc-ARSJ	Magenta
NR2F2-AS1	Magenta
lnc-HNRNPA0	Magenta
lnc-EFEMP1	Magenta
lnc-PIK3R1	Magenta
lnc-CYBB	Magenta
lnc-SMOC2	Magenta
LINC01322	Magenta
lnc-RASGRP3	Magenta
lnc-CCDC184	Magenta
lnc-NSF	Magenta
lnc-MRPS30	Magenta
lnc-AKR1C3	Magenta
lnc-ALDH1B1	Magenta
lnc-ARHGAP29	Magenta
lnc-FBXO33	Magenta
lnc-IL6	Magenta
lnc-PFKFB3	Magenta
lnc-GIMAP5	Magenta
ZBED3-AS1	Magenta
lnc-CCDC87	Magenta
lnc-TOX3	Magenta

© 2022. Luo D et al. Published online at http://www.aginganddisease.org/EN/10.14336/AD.2022.0607

lnc-ATP2B2	Magenta
lnc-IRS1	Magenta
lnc-TXNDC5	Magenta
lnc-PERP	Magenta
LINC02062	Magenta
APTR	Magenta
lnc-UBLCP1	Magenta
lnc-KHDRBS3	Magenta
lnc-TRMT9B	Magenta
lnc-RFX7	Magenta
lnc-ATAD2B	Magenta
lnc-RFX7	Magenta
lnc-AKIRIN1	Magenta
lnc-HSF2	Magenta
LINC02015	Magenta
lnc-SLC39A6	Magenta
SMC5-AS1	Magenta
lnc-ARX	Magenta
LINC-PINT	Magenta
lnc-NEFM	Magenta
lnc-RNASEH1	Magenta
lnc-FAM86B2	Magenta
LINC01473	Magenta
lnc-NIM1K	Magenta
lnc-GPA33	Magenta
lnc-RTTN	Magenta
lnc-AADAT	Magenta
lnc-TSN	Magenta
lnc-PCDH10	Magenta
lnc-CORO7-PAM16	Magenta
lnc-DNAH12	Magenta
lnc-ZFP42	Magenta
lnc-MEIS1	Magenta
lnc-ITGA2	Magenta
lnc-TRAF5	Magenta
lnc-DUSP22	Magenta
lnc-MTRF1	Magenta
lnc-ZNF100	Magenta
lnc-ZNF131	Magenta
lnc-F11	Magenta
lnc-ZNF385C	Magenta
lnc-CTNNA3	Magenta
lnc-CNOT2	Magenta
lnc-RHOU	Magenta
lnc-NXPH1	Magenta
lnc-GREM1	Magenta

© 2022. Luo D et al. Published online at http://www.aginganddisease.org/EN/10.14336/AD.2022.0607

lnc-SETD7	Magenta
lnc-RTP4	Magenta
lnc-VN1R2	Magenta
lnc-ARHGEF26	Magenta
LINC02485	Magenta
lnc-CEP128	Magenta
lnc-AMIGO2	Magenta
lnc-ABHD12	Magenta
MIR3936HG	Magenta
lnc-RASA1	Magenta
OLMALINC	Magenta
lnc-GUCA2B	Magenta
lnc-SERTM2	Magenta
lnc-HTR1B	Magenta
lnc-BCL11A	Magenta
lnc-NUTM2B	Magenta
lnc-FAM69C	Magenta
lnc-OR5A2	Magenta
lnc-ARSG	Magenta
lnc-ZNF747	Magenta
lnc-EIF4EBP3	Magenta
lnc-VPS11	Magenta
GAS6-DT	Magenta
lnc-PNO1	Magenta
lnc-PRSS27	Magenta
lnc-CWC15	Magenta
lnc-SLC25A28	Magenta
lnc-SKP1	Magenta
SOX1-OT	Magenta
lnc-TFEC	Magenta
lnc-DKK4	Magenta
lnc-GABRG1	Magenta
ZRANB2-AS2	Magenta
lnc-FBXO11	Magenta
lnc-ADGRA3	Magenta
lnc-HAND2	Magenta
lnc-APPL2	Magenta
lnc-AGO2	Magenta
lnc-SP110	Magenta
lnc-SORCS1	Magenta
lnc-CXorf51B	Magenta
lnc-HPGDS	Magenta
GPC5-AS1	Magenta
lnc-NAA38	Magenta
lnc-RPGR	Magenta
lnc-EIF2AK4	Magenta

© 2022. Luo D et al. Published online at http://www.aginganddisease.org/EN/10.14336/AD.2022.0607

hsa_circ_0137606	Magenta
hsa_circ_0106983	Magenta
hsa_circ_0021502	Magenta
hsa_circ_0007458	Magenta
hsa_circ_0002599	Magenta
lnc-KLHL14	Lightcyan
ARHGEF26-AS1	Lightcyan
LINC00504	Lightcyan
lnc-PABPC4L	Lightcyan
lnc-OTUD4	Lightcyan
lnc-MBTD1	Lightcyan
lnc-USP46	Lightcyan
lnc-HERC5	Lightcyan
lnc-GSDMC	Lightcyan
lnc-KLHL14	Lightcyan
SMILR	Lightcyan
lnc-LHFPL6	Lightcyan
LINC01814	Lightcyan
lnc-COMMD10	Lightcyan
lnc-KBTBD3	Lightcyan
lnc-ZCCHC17	Lightcyan
lnc-STMND1	Lightcyan
lnc-RPRD1A	Lightcyan
lnc-PTGDR	Lightcyan
lnc-FOXG1	Lightcyan
lnc-CDH18	Lightcyan
lnc-PRDM9	Lightcyan
lnc-PITRM1	Lightcyan
lnc-PROKR2	Lightcyan
lnc-ZNF708	Lightcyan
lnc-RTL9	Lightcyan
lnc-FOXD4L6	Lightcyan
lnc-BLID	Lightcyan
lnc-TMEM176B	Lightcyan
lnc-SORCS3	Lightcyan
PCAT1	Lightcyan
lnc-FRG2	Lightcyan
lnc-H2BFWT	Lightcyan
lnc-GLG1	Lightcyan
lnc-TRIM62	Lightcyan
LINC02252	Lightcyan
HCG23	Lightcyan
lnc-DAPP1	Lightcyan
lnc-PTGDR	Lightcyan
lnc-WRNIP1	Lightcyan
RABGAP1L-IT1	Lightcyan

© 2022. Luo D et al. Published online at http://www.aginganddisease.org/EN/10.14336/AD.2022.0607

TOR4A	Lightcyan
LINC01013	Lightcyan
lnc-CHRNA7	Lightcyan
lnc-ZNF546	Lightcyan
lnc-DYM	Lightcyan
lnc-CPOX	Lightcyan
lnc-TIMM9	Lightcyan
lnc-PRDM13	Lightcyan
lnc-FYB2	Lightcyan
lnc-ATG5	Lightcyan
lnc-VIP	Lightcyan
lnc-NUTM2B	Lightcyan
lnc-ZMYM3	Lightcyan
lnc-ANKRD20A2	Lightcyan
lnc-CD1B	Lightcyan
lnc-DHRS4L2	Lightcyan
NUTM2B-AS1	Lightcyan
lnc-ZNF585A	Lightcyan
lnc-DRICH1	Lightcyan
lnc-MTERF4	Lightcyan
lnc-IGSF9	Lightcyan
lnc-NDFIP2	Lightcyan
lnc-EMP2	Lightcyan
lnc-PTBP2	Lightcyan
hsa_circ_0003718	Lightcyan
lnc-HNRNPD	Lightcyan
lnc-XCL2	Lightcyan
lnc-ACTN2	Lightcyan
lnc-CDK19	Lightcyan
lnc-SETD6	Lightcyan
lnc-MEIS2	Lightcyan
lnc-MAB21L1	Lightcyan
LINC00461	Lightcyan
lnc-GUCY1A1	Lightcyan
lnc-ANKRD33B	Lightcyan
lnc-ADAM17	Lightcyan
FNTB	Lightcyan
lnc-PCDH20	Lightcyan
lnc-CCM2	Lightcyan
lnc-SVIP	Lightcyan
lnc-ANKFY1	Lightcyan
lnc-RC3H1	Lightcyan
lnc-CTAGE6	Lightcyan
lnc-ODF3L1	Lightcyan
lnc-GLRX	Lightcyan
lnc-TRAPPC11	Lightcyan

© 2022. Luo D et al. Published online at http://www.aginganddisease.org/EN/10.14336/AD.2022.0607

lnc-ART1	Lightcyan
lnc-GUCY1A2	Lightcyan
lnc-FZD4	Lightcyan
PLSCR3	Lightcyan
lnc-PLSCR3	Lightcyan
lnc-FNTA	Lightcyan
HBB	Lightcyan
lnc-RIC8A	Lightcyan
lnc-PPIL2	Lightcyan

Supplementary Table 11. Information on key DEmRNAs, DElncRNAs, and DEcircRNAs.

mRNAs

GeneID	Log_2FC	*P*-value
CREBBP	-0.63	0.0018
NCOR2	-1.26	0.0047
RELA	-0.6	0.0189
NOTCH1	-1.37	0.0016
JAK3	-1.4	0.003
PDGFRB	-0.95	0.0327
PLCG1	-0.6	0.0403
PIK3CD	-0.72	0.0129
ERBB3	-1.16	0.0002
TYK2	-1.33	0.0023

lncRNAs

GeneID	Log_2FC	*P*-value
KCNQ1OT1	-2.82	0.0001
NEAT1	-2.78	0.0178
OIP5-AS1	-1.1	0.0292
FGD5-AS1	-3.93	0.0183
GAS5	1.86	0.0199
CYP1B1-AS1	-2.14	0.0064
FTX	-2.03	0.0031
LINC00174	-1.79	0.012
WDFY3-AS2	3.65	0.049
FAM95B1	-2.22	0.0397

circRNAs

CircbaseID	Parent-gene	Log_2FC
hsa_circ_0031668	RALGAPA1	3.8
hsa_circ_0087493	IARS1	1.63
hsa_circ_0102923	CPSF2	3.02

© 2022. Luo D et al. Published online at http://www.aginganddisease.org/EN/10.14336/AD.2022.0607

hsa_circ_0089902	OFD1	3.6
hsa_circ_0135423	RIMS2	3.42
hsa_circ_0010117	SPEN	1.46
hsa_circ_0113947	WDR78	3.46
hsa_circ_0014391	UBAP2L	3.37
hsa_circ_0136539	ANK1	1.7
hsa_circ_0008776	THSD1	2.07

© 2022. Luo D et al. Published online at http://www.aginganddisease.org/EN/10.14336/AD.2022.0607
